# The dual role of CD70 in B‐cell lymphomagenesis

**DOI:** 10.1002/ctm2.1118

**Published:** 2022-12-05

**Authors:** Man Nie, Weicheng Ren, Xiaofei Ye, Mattias Berglund, Xianhuo Wang, Karin Fjordén, Likun Du, Yvonne Giannoula, Dexin Lei, Wenjia Su, Wei Li, Dongbing Liu, Johan Linderoth, Chengyi Jiang, Huijing Bao, Wenqi Jiang, Huiqiang Huang, Yong Hou, Shida Zhu, Gunilla Enblad, Mats Jerkeman, Kui Wu, Huilai Zhang, Rose‐Marie Amini, Zhi‐Ming Li, Qiang Pan‐Hammarström

**Affiliations:** ^1^ Department of Medical Oncology State Key Laboratory of Oncology in South China Collaborative Innovation Center for Cancer Medicine Sun Yat‐sen University Cancer Center Guangzhou China; ^2^ Department of Biosciences and Nutrition Karolinska Institutet Stockholm Sweden; ^3^ Department of Immunology Genetics and Pathology Uppsala University Uppsala Sweden; ^4^ Department of Lymphoma National Clinical Research Center of Cancer Key Laboratory of Cancer Prevention and Therapy Tianjin Medical University Cancer Institute and Hospital Tianjin China; ^5^ Department of Oncology Skåne University Hospital Lund Sweden; ^6^ BGI‐Shenzhen Shenzhen China; ^7^ Guangdong Provincial Key Laboratory of Human Disease Genomics Shenzhen Key Laboratory of Genomics BGI‐Shenzhen Shenzhen China; ^8^ Department of Hematology Jilin Cancer Hospital Changchun China

**Keywords:** CD70, genetic aberration, diffuse large B‐cell lymphoma, immune evasion, HBV infection

## Abstract

**Background:**

CD70 is a costimulatory molecule that is transiently expressed on a small set of activated lymphocytes and is involved in T‐cell‐mediated immunity. However, the role of CD70 in B‐cell malignancies remains controversial.

**Methods:**

We investigated the clinical relevance of *CD70* genetic alterations and its protein expression in two diffuse large B‐cell lymphoma (DLBCL) cohorts with different ethnic backgrounds. We also performed transcriptomic analysis to explore the role of CD70 alterations in tumour microenvironment. We further tested the blockade of CD70 in combination with PD‐L1 inhibitor in a murine lymphoma model.

**Results:**

We showed that *CD70* genetic aberrations occurred more frequently in the Chinese DLBCL cohort (56/233, 24.0%) than in the Swedish cohort (9/84, 10.8%), especially in those with concomitant hepatitis B virus (HBV) infection. The *CD70* genetic changes in DLBCL resulted in a reduction/loss of protein expression and/or CD27 binding, which might impair T cell priming and were independently associated with poor overall survival. Paradoxically, we observed that over‐expression of CD70 protein was also associated with a poor treatment response, as well as an advanced disease stage and EBV infection. More exhausted CD8^+^ T cells were furthermore identified in CD70 high‐expression DLBCLs. Finally, in a murine lymphoma model, we demonstrated that blocking the CD70/CD27 and/or PD1/PD‐L1 interactions could reduce CD70^+^ lymphoma growth in vivo, by directly impairing the tumour cell proliferation and rescuing the exhausted T cells.

**Conclusions:**

Our findings suggest that CD70 can play a role in either tumour suppression or oncogenesis in DLBCL, likely via distinct immune evasion mechanisms, that is, impairing T cell priming or inducing T cell exhaustion. Characterisation of specific dysfunction of CD70 in DLBCL may thus provide opportunities for the development of novel targeted immuno‐therapeutic strategies.

## Graphical abstract

1 CD70 mutations/deletions result in reduction/loss of CD70 expression and are enriched in patients with concomitant HBV infection.

2 Overexpression of CD70 is associated with EBV infection and a more immunosuppressive tumour microenvironment.

3 CD70 plays both tumour suppressive and oncogenic roles in diffuse large B‐cell lymphoma.

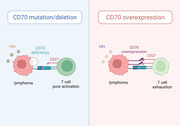

## 1 INTRODUCTION

Diffuse large B‐cell lymphoma (DLBCL) is the most common type of adult non‐Hodgkin lymphoma (NHL), accounting for approximately 30% of newly diagnosed cases every year. Although standard‐of‐care immunotherapy has greatly improved the clinical outcome, approximately one‐third of DLBCL patients still experience relapse or refractory disease.^1^ Gene expression profiling has led to the identification of at least two distinct subtypes, the germinal center B‐cell‐like (GCB) and activated B‐cell‐like (ABC) subtypes, with the latter being associated with poor patient survival.^2^ Recent studies employing next‐generation sequencing technologies have further identified a large number of genetic alterations in DLBCL^3^, ^4^, and integrative analyses of these genetic alterations have led to the classification of several molecular subtypes with different clinical outcomes.^5^, ^6^, ^7^ Most of the genetic changes identified in various molecular subtypes of DLBCL, however, remain poorly characterised, and understanding the functional and clinical relevance of these alterations is a key to the development of effective therapeutic targets for DLBCL.

CD70 is a costimulatory molecule that is transiently expressed on activated B and T lymphocytes and myeloid cells. Previous studies have suggested that CD70, through binding to its receptor CD27, is required for germinal centre formation, B‐cell activation, T‐cell expansion and survival, and NK‐cell function.^8^, ^9^ We and others have previously reported that loss‐of‐function germline mutations in the *CD70* gene lead to an autosomal recessive form of inborn errors of immunity, which is characterised by Epstein‒Barr virus (EBV)‐associated lymphoproliferative disease, Hodgkin's lymphoma, and/or hypogammaglobulinemia.^10^, ^11^, ^12^ We have further shown that memory T cells and cytotoxic T‐cell activity against EBV^+^ B cells were reduced in these patients due to CD70 deficiency, suggesting that the CD27–CD70 interaction plays a nonredundant role in T‐cell mediated immunity, especially for protection against viral infections such as EBV infection.^10^ Constitutive expression of CD70 in a transgenic mouse model has shown that the CD27–CD70 interaction can enhance the expansion and activity of antigen‐specific CD8^+^ T cells and protect these mice from otherwise lethal tumours.^13^. Taken together, CD70‐mediated T‐cell response may be involved in antitumour response, either through elimination of malignant cells or virus‐infected cells.

DLBCL cells, which are transformed from B cells that function as professional antigen‐presenting cells, can express both major histocompatibility complex (MHC) class I and class II molecules.^14^ Interaction of the MHC/tumour antigen epitope complex with the T‐cell receptor and costimulatory signals (including CD70/CD27) may lead to the activation of CD4^+^ and CD8^+^ T cells.^14^ Somatic mutations in *CD70*, like W75*, S84A and R138C, or copy number loss of *CD70*, have previously been identified in several DLBCL cohorts.^15^, ^16^, ^17^, ^18^ However, it remains unclear whether these genetic alterations can lead to the loss of CD70 function and furthermore influence the patient survival.

Ectopic or aberrant expression of CD70 has also been demonstrated in a wide spectrum of solid tumours and haematologic malignancies, including DLBCL, which could contribute to poor survival.^15^, ^19^ It has been suggested that CD70^+^ tumour cells can be associated with an increased proportion of Foxp3^+^CD4^+^CD25^−^ T cells^20^ or tumour‐associated macrophages (TAMs)^21^, thereby creating an immunosuppressive tumour microenvironment. Moreover, CD70/CD27 coexpression has been detected in acute myeloid leukaemia (AML) blasts and stem/progenitor cells, and its expression activates the stem cell gene expression program.^22^ Targeting/blocking CD70/CD27 signalling thus represents an attractive therapeutic strategy for multiple types of cancers.^23^, ^24^

To further dissect the role of CD70 in B‐cell malignancies, which hypothetically could be either tumour suppressive or oncogenic, we conducted a comprehensive study to investigate the functional and clinical relevance of genetic alterations in the *CD70* gene and the protein expression pattern of CD70 in two independent cohorts of DLBCL patients with different ethnic backgrounds and viral infection statuses. We also performed transcriptomic analysis on bulk tumour tissues as well as single‐cell samples, to study the impact of *CD70* alterations in tumour microenvironment (TME). We further explored the therapeutic potential of blocking the CD70/CD27 interaction in combination with the immune checkpoint inhibitor anti‐PD‐L1 in a murine DLBCL model.

## 2 METHODS

### 2.1 Patient materials

**FIGURE 1:**
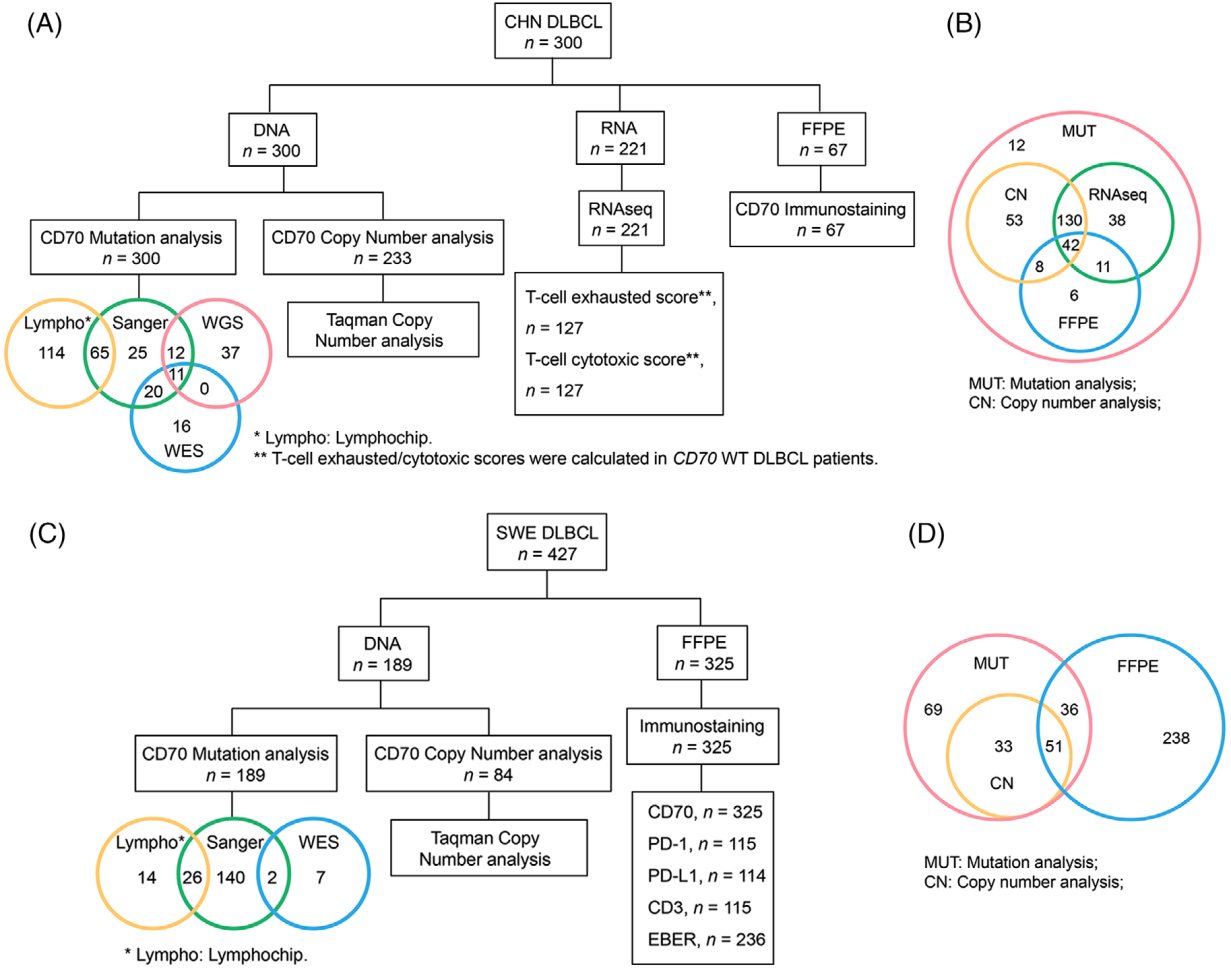
The workflow of the diffuse large B‐cell lymphoma (DLBCL) cohorts. The workflow of the Chinese (A,B) and Swedish (C,D) DLBCL cohorts. In *CD70* mutation analysis, some samples were examined by more than one sequencing method. Detailed information of each patient is shown in Tables S1 and S2 respectively.

Three‐hundred Chinese DLBCL patients and 427 Swedish DLBCL patients were included in this study. The inclusion criteria included the following: (a) pathologically confirmed DLBCL based on the WHO classification of haematopoietic and lymphoid tumours; (b) available tumour tissues and/or peripheral blood mononuclear cells (PBMCs); and (c) available clinical data. The exclusion criteria were as follows: (a) primary or acquired immunodeficiency diseases and (b) autoimmune diseases. Patients were classified into GCB and non‐GCB subtypes according to the Hans algorithm. The details of the Chinese and Swedish cohorts are presented in Tables S1 and S2, respectively. Briefly, for the Chinese DLBCL cohort, which has been partially described previously,^16^, ^17^ the patients were recruited between 2007 and 2015 from Sun Yat‐Sen University Cancer Center and Tianjin Medical University Cancer Institute and Hospital. A total of 300 frozen tumour biopsy specimens, 96 paired blood samples, and 67 matched formalin‐fixed paraffin‐embedded (FFPE) samples were available for this cohort for the detection of *CD70* mutations, copy number changes and/or protein expression (Figure 1A,B, Table S1). Of these, 115 were GCB and 180 were non‐GCB subtype, 248 were taken at diagnosis, and 52 were taken at relapse; 61 were HBsAg^+^ (Hepatitis B surface antigen) and 13 were EBER^+^ (EBV‐encoded small RNA in situ hybridisation); 132 and 73 patients were treated with R‐CHOP and CHOP regimen, respectively. For the Swedish DLBCL cohort, patients were recruited between 1982–2012 from Uppsala University and Skåne University Hospital. A total of 189 frozen tumour biopsies, 9 paired blood samples, 87 matched FFPE samples and 238 additional FFPE samples were available for various analyses (Figure 1C,D, Table S2). Of these, 114 were the GCB subtype and 110 were the non‐GCB subtype. All Swedish patients were negative for HBsAg, and 22 patients were EBER^+^. Ninety‐seven patients were treated with the R‐CHOP regimen, and 246 were treated with the CHOP regimen. The study was approved by the institutional review boards at the Tianjin Medical University Cancer Institute, the Sun Yat‐Sen University Cancer Center, the Karolinska Institutet and the Uppsala University.

### 2.2 Whole genome and whole exome sequencing

Whole‐genome sequencing (WGS, *n* = 60) and whole‐exome sequencing (WES, *n* = 56) were performed on all paired tumour/control samples (altogether 105 paired samples, 11 paired samples were sequenced by both WES and WGS). Sequencing was performed in BGI‐Shenzhen using the platforms previously described.^17^ Tumour‐in‐normal contamination (DeTiN) was applied to estimate the amount of tumour cell contamination in the paired control PBMC samples.^25^. A TiN value less than 0.15 indicates no or mimical level of tumour cell contamination. All except one PBMC sample tested have a TiN value less than 0.15, suggesting negligible tumour cell contamination in our paired control samples. The CD70 sequencing data on the only PBMC sample with TiN value more than 0.15 were manually checked by Integrative Genomics Viewer. The criteria for the detection of somatic mutations were as follows: (a) coverage of at least 10 reads; (b) mutation allele frequency of at least 10% in the tumours; and (c) minor allele frequency of less than 1% in the paired controls. All reported variants passed visual inspection using the Integrative Genomics Viewer.

### 2.3 Targeted sequencing

The targeted sequencing panel lymphochip consists of the entire coding regions of 212 genes. A total of 179 Chinese DLBCL samples and 40 Swedish DLBCL samples were analysed using the lymphochip as described previously.^17^ One hundred nanograms of DNA from each sample were used for library preparation. The sequencing analysis and procedure for the identification and validation of potential somatic mutations have been described previously.^17^ The coding regions of *CD70* were also analysed by Sanger sequencing in 133 Chinese tumour samples and 168 Swedish tumour samples.

### 2.4 Transcriptome sequencing

Transcriptome sequencing was performed on RNA extracted from 221 Chinese DLBCL samples, 97 of which have been reported previously.^17^ The sequencing libraries were sequenced on the DNBSEQ or Illumina HiSeq 2000 platform. The number of transcripts per million was used to determine gene expression levels. The batch effect was removed by normalising the log2‐transformed transcripts per million values^26^. The normalised expression values were analysed by Qlucore Omics Explorer (Qlucore AB). The infiltration of immune cell types was predicted by using the online tool xCell^27^ and the malignant B‐cell states and ecotypes were predicted using the online tool EcoTyper.^28^ The T‐cell‐cytotoxic score and T‐cell‐exhausted score were calculated with the mean absolute deviation modified *Z* score (ZMAD)‐normalised mRNA value of all T‐cell cytotoxic signature genes (*CCL4*, *CST7*, *PRF1*, *GZMA*, *GZMB*, *IFNG* and *CCL3*) and T‐cell exhausted signature genes (*PDCD1, CD274*, *CTLA4*, *TIGIT*, *LAG3* and *HAVCR2*) in each sample, respectively, using the methods described previously.^29^

### 2.5 Quantification of CD70 copy number by qPCR

*CD70* copy number changes were detected by quantitative polymerase chain reaction (qPCR) using the TaqMan Copy Number Assay (Thermo Fisher) in 233 Chinese DLBCL samples and 84 Swedish DLBCL samples. The TaqMan^®^ Genotyping Master Mix (Thermo Fisher) was used for qPCR reactions and samples were performed on 7500 Fast Real‐Time PCR System (Applied Biosystems). The data were then imported and analysed in CopyCaller^®^ Software (Thermo Fisher). *CD70* data were normalised to RNase P and calibrated to the copy number of SU‐DHL10 cells, which have two copies of *CD70*.

### 2.6 CD70 protein expression and CD27 binding assay

*CD70* mutants were generated by site‐directed mutagenesis and cloned into a pcDNA3.1 mammalian expression vector (Life Technologies). The cells were lysed 48 h after transfection, and CD70 expression was detected by Western blotting using a rabbit anti‐human CD70 monoclonal antibody (orb213695, Biorbyt, recognising amino acids 76–115, Cambridge, UK). β‐Actin was served as a loading control (#4967; Cell Signaling, Danvers, MA). The CD27 binding assay was performed as previously described^10^ and followed the FACS Protocol (Binding) from Acrobiosystems. HEK293T cells were transfected with the CD70 wild‐type (WT) or mutant plasmids. After 24 h, the cells were harvested, resuspended in phosphate buffered saline solution (PBS)/0.5% fetal bovine serum (FBS) and incubated for 45 min on ice with 8 μl of biotinylated CD27‐mIg (AcroBiosystems) or 8 μl of biotinylated anti‐human CD5 mAb (Invitrogen). This step was followed by incubation for 30 min on ice with 100 μl (0.3 μg) of streptavidin‐PE (Invitrogen), which was detected by flow cytometry. Flow cytometry data were analysed with FlowJo software.

### 2.7 Immunohistochemical staining

Immunohistochemical (IHC) staining (CD70, PD‐L1 and CD3) and slide scanning were performed at the FoUU Clinical Pathology Service at Uppsala University Hospital. Sections were subjected to heat‐induced antigen retrieval for 20 min at 97°C in Tris EDTA buffer (CD70, PD‐L1 and CD3) or citrate buffer (PD‐1) (PT‐link, DAKO, Agilent, Santa Clara, CA). Specimens were incubated with primary antibodies specific for human CD70 (MAB2738, clone 301731, 1:100 dilution, R&D Systems), PD‐1 (ab52587, clone NAT105, 1:200 dilution, Abcam), PD‐L1 (13684S, clone E1L3N, 1:200 dilution, Cell Signaling Technology), or CD3 (IR503, polyclonal, RTU, DAKO, Agilent) in a DAKO autostainer Link 48 instrument using an Envision Flex detection kit (DAKO) according to the manufacturer's instructions. Primary antibodies specific for PD‐1 (ab214421, 1:100 dilution, Abcam) and granzyme B (ab4059, 1:100 dilution, Abcam) were used for murine DLBCL specimens. Image acquisition was performed on a NanoZoomer S60 (Hamamatsu Photonics K.K. Tokyo, Japan). The expression level was scored semiquantitatively based on the staining intensity and distribution using the Allred score as described elsewhere.^30^ Briefly, the Allred score = SI (staining intensity) + PP (percentage of positive cells). SI was assigned as follows: 0 = negative; 1 = weak; 2 = moderate; and 3 = strong. PP was defined as 0 = 0%; 1 = 0%–5%; 2 = 5%–25%; 3 = 25%–50%; 4 = 50%–75%; and 5 = 75%–100%. For categorisation of the continuous CD70 values into negative and positive, commonly used cutoff points for the measurements were used (range 0–8, negative = 0–2, positive = 3–8).^20^

### 2.8 Murine lymphoma model

Female BALB/c mice were purchased from Guangdong Medical Lab Animal Center and housed in a specific pathogen‐free mouse facility. Murine A20 lymphoma cells^31^ (1×10^6^/100 μl), derived from BALB/c mice, were injected subcutaneously into the flank. When the tumour was palpable, cohorts of mice were intraperitoneally injected with IgG (BioXCell, BE0090), 300 μg of anti‐CD70 (BioXCell, BE0022), 200 μg of anti‐PD‐L1 (BioXCell, BE0101), or a combination of these antibodies every 3 days for a total of 4 times. Tumour growth was evaluated by measuring the tumour volume every 3 days. When the mice were sacrificed, the tumours were harvested for further investigation. The institutional animal care and use committee of Sun Yat‐Sen University approved the study.

### 2.9 Statistics

Survival was analysed by the Kaplan‒Meier method and compared using the log‐rank test. Other statistical analyses, like Student's *t*‐test, Mann‒Whitney *U*‐test,  χ^2^‐test and one‐way ANOVA with Tukey's test, were performed as appropriate and the details were described in the corresponding figure legends. *P*‐values less than 0.05 were considered statistically significant.

## 3 RESULTS

### 3.1 The CD70 gene is frequently targeted by somatic mutations/deletions in diffuse large B‐cell lymphoma

**FIGURE 2:**
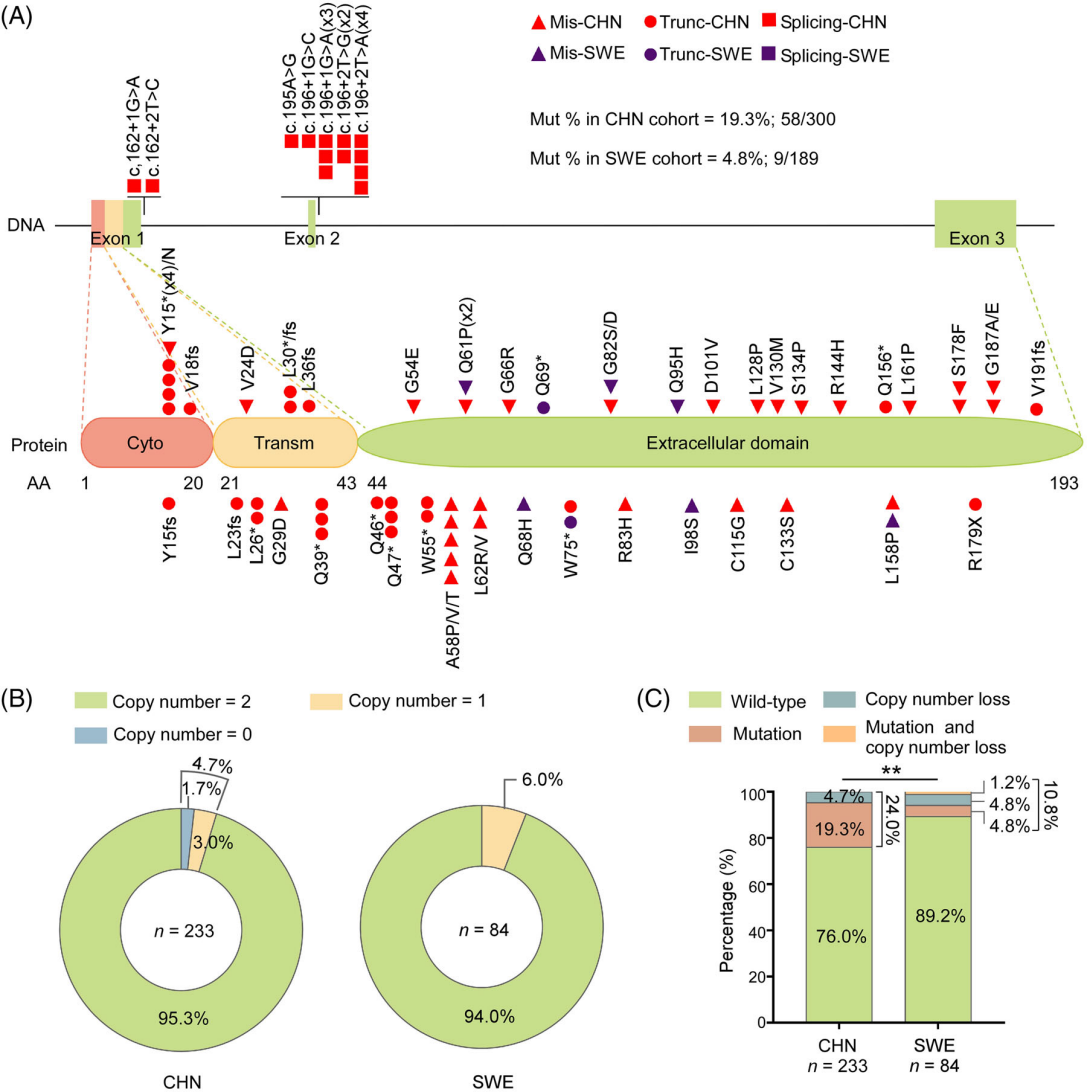
The *CD70* gene is frequently targeted by mutations and deletions in diffuse large B‐cell lymphoma (DLBCL). (A) Schematic diagram representing the distribution of mutations identified in the *CD70* gene in tumour samples from Chinese (CHN, red symbols) and Swedish DLBCL (SWE, purple symbols) patients. Mis, missense mutation; Mut, mutation; Trunc, truncation mutation. (B) Distribution of *CD70* homozygous (copy number = 0) and heterozygous (copy number = 1) deletions in CHN and SWE DLBCL tumour samples. χ^2^‐test, *P* > 0.6585. (C) Comparison of the frequency of *CD70* mutations and copy number variations in CHN and SWE DLBCL tumour samples. Mut and copy number loss: mutation and copy number loss; χ^2^‐test, **P = 0.0095.

*CD70* mutations^5^, ^7^, ^16^, ^17^, ^32^ or deletions^15^ have previously been reported in DLBCL cases; however, the functional consequence and clinical relevance of genetic aberrations (mutations and copy number loss) affecting *CD70* remain to be uncertain. We thus first integrated the data from the Chinese DLBCL cohort, which includes WGS and WES data from 96 pairs of tumour/control samples and targeted sequencing data from 204 tumour‐only samples, to identify somatically occurring (tumour‐specific) mutations in *CD70*. Altogether, 70 nonsilent variants were identified in 58 of 300 tumour biopsies obtained from Chinese DLBCL patients (19.3%; Figure 2A, Table S3). Thirty‐two of these variants were determined as somatically occurring mutations based on sequencing of both the tumour and the paired nontumour PBMC samples. Thirty‐eight remaining variants were identified in tumour‐only samples and were predicted to be most likely somatic origin after strict filtering steps for potential germline mutations. Among all nonsilent variants, 29 corresponded to nonsense mutations or inactivating frameshift insertions/deletions (indels), resulting in transcripts that may encode truncated CD70 proteins. The remaining variants either affected splicing (*n* = 13) or were missense mutations (*n* = 28). The median variant allele frequency was 40.0% (range: 4%–85.7%), suggesting that most mutations were in the major tumour clones (Table S3). *CD70* mutations were distributed at a comparable frequency in both the GCB and non‐GCB DLBCL subtypes (24.1% vs. 18.4%), and no significant difference in mutation frequency was observed between samples obtained at diagnosis and relapse (21.1% vs. 16.2%) (Figure S1A,B).

Ethnic background may influence the mutational spectrum in the DLBCL genome, and a higher rate of *CD70* mutations has been noted in Chinese DLBCL patients than in published Western cohorts.^33^ To validate this observation, we next performed mutation analysis of the *CD70* coding exons in 189 Swedish DLBCL samples (Table S2) and identified 10 nonsilent variants (Figure 2A, Table S4). The mutation rate of *CD70* in the Chinese cohort was indeed significantly higher than that in the Swedish cohort (19.3% vs. 4.8%, χ^2^‐test, *P* < 0.0001), as well as the published Western DLBCL cohorts^5^, ^32^, ^34^, ^35^, ^36^ (ranging from 1.9% to 9.6%; Figure S2).

We next analysed copy number variations (CNVs) in 233 of the 300 Chinese DLBCL biopsies and 84 of the 189 Swedish DLBCL cases by a TaqMan predesigned *CD70* copy number assay. The analysis revealed a 4.7% and 6.0% of *CD70* copy number loss in the Chinese and Swedish cohorts, respectively (Figure 2B). Consistent with a previous study which showed that *CD70* copy number gain was very rare,^15^ no copy number gain was identified in either cohort.

The combined analysis for samples with both mutation and copy number data revealed that the overall frequency of *CD70* genetic aberrations was significantly higher in the Chinese cohort (24.0%; *n* = 233) than in the Swedish DLBCL samples (10.8%; *n* = 84) (Figure 2C). Notably, except in haematopoietic and lymphoid malignancies, *CD70* genetic alterations were extremely rare in cancers, as mutations and/or CNV loss were observed in less than 1.5% of 35 993 tumour samples included in the COSMIC database (Tables S5a and S5b).

### 3.2 CD70 genetic alterations resulted in reduced/loss of protein expression or CD27 binding

**FIGURE 3:**
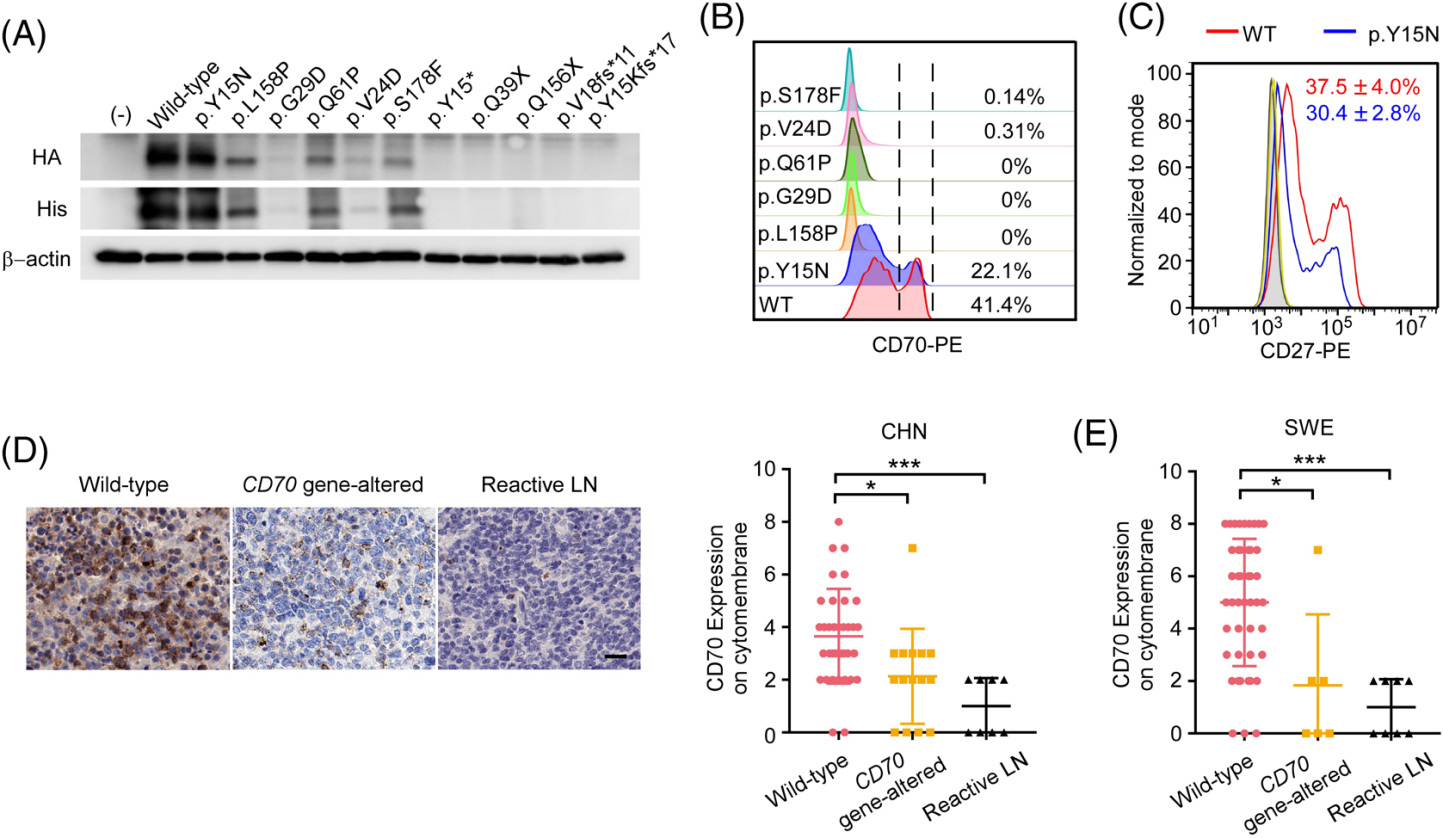
***CD70*** genetic alterations resulted in reduced/loss of protein expression or CD27 binding. (A) Immunoblotting analysis of exogenous CD70 expression in HEK293T cells transfected with equimolar amounts of vectors expressing HA‐His‐tagged wild‐type (WT) or mutant (identified from our clinical cohorts) *CD7*0 alleles. Representative images of three independent experiments are shown. (B) CD70 expression on the cytomembrane was analysed by flow cytometry using a mAb against CD70‐transfected HEK293T cells. Three independent experiments were performed. (C) The binding of overexpressed WT (red) or mutant CD70 (p.Y15N) (blue) to recombinant human CD27 was measured by flow cytometry. The shaded area represents nontransfected HEK293T cells. The binding of anti‐CD5 mAb to cells transfected with WT (yellow) is shown. Three independent experiments were performed. (D) Representative images of CD70 staining by immunohistochemical (IHC) (left panel) and quantification of CD70 expression on the cytomembrane (right panel) in Chinese diffuse large B‐cell lymphoma (DLBCL) samples and reactive lymph nodes. Scale bar, 50 μm. Mann–Whitney *U*‐test, *P = 0.0163, ***P = 0.0005. (E) Quantification of CD70 expression on the cytomembrane by IHC in Swedish DLBCL samples. The same reactive lymph node samples were used. Mann–Whitney *U*‐test, *P = 0.0285, **P = 0.0006.

Most of the *CD70* mutations identified in our cohorts were predicted to affect protein structure or function by in silico tools (Tables S3 and S4). To further evaluate the functional consequence of these mutations, we next transfected plasmids encoding WT and selected mutated *CD70* alleles (*n* = 11) into HEK293T cells. For the tested nonsense or frameshift mutations (*n* = 5), neither the full length nor the truncated form of CD70 was detected (Figure 3A). For the investigated missense mutations (*n* = 6), CD70 proteins were expressed at a normal or reduced level, likely due to increased degradation (Figure S3A–S3F). Two missense mutations (G29D and V24D) occur in the transmembrane domain and low amounts of the soluble form of CD70 were detected in the supernatant compared to the amounts from cell lysate (Figure S3G). Furthermore, with the exception of the p.Y15N mutant, all tested missense mutations resulted in a loss of or significantly reduced cell surface expression and thus binding capacity to CD27, which is a functional readout for CD70 (Figure 3B,C, S4A,B). The cells transfected with p.Y15N mutant expressed at a similar level as the cells transfected with WT plasmid when different amounts of plasmids were transfected (Figure S4A). However, the CD27 binding affinity was comparatively lower in the cells transfected with p.Y15N mutant (Figures 3C, S4B). The p.Y15N mutation was only identified in the Case‐92 sample, which also harboured another tested loss‐of‐expression frameshift mutation (p.Y15Kfs*17). We subsequently performed IHC staining and indeed observed a comparatively low level of CD70 expression in this sample (Figure S5A,B).

We further analysed CD70 protein expression in 50 Chinese DLBCL samples with a known genetic status by IHC. Nine samples from reactive lymph nodes were included as controls. Only some scattered cells stained positive for CD70 in normal lymph nodes, whereas abundant cytomembrane expression of CD70 was observed in *CD70* WT DLBCL (Figure 3D). Significantly lower protein expression was observed in tumour cells from samples harbouring *CD70* genetic alterations (*n* = 15) compared with those with WT *CD70* (*n* = 35) (Figures 3D). In one outlier (Case‐32) from the gene‐altered group, the mutant allele was not expressed based on the RNA‐seq analysis, suggesting that the detected protein originated from the WT allele in this sample (Figure S5A). In Swedish DLBCL samples with a known genetic status of *CD70* (*n* = 51), we also observed that the *CD70* gene‐altered group (*n* = 6) had lower CD70 protein expression than the WT group (*n* = 45) (*P* < 0.05; Figure 3E, S5C). Taken together, in a majority of the cases, the identified *CD70* genetic alterations from DLBCL samples resulted in a loss/reduction of CD70 protein expression in the tumour cells and/or loss of function (CD27 binding).

### 3.3 Hepatitis B virus infection contributes to the increased CD70 genetic aberrations in Chinese diffuse large B‐cell lymphoma samples

**FIGURE 4:**
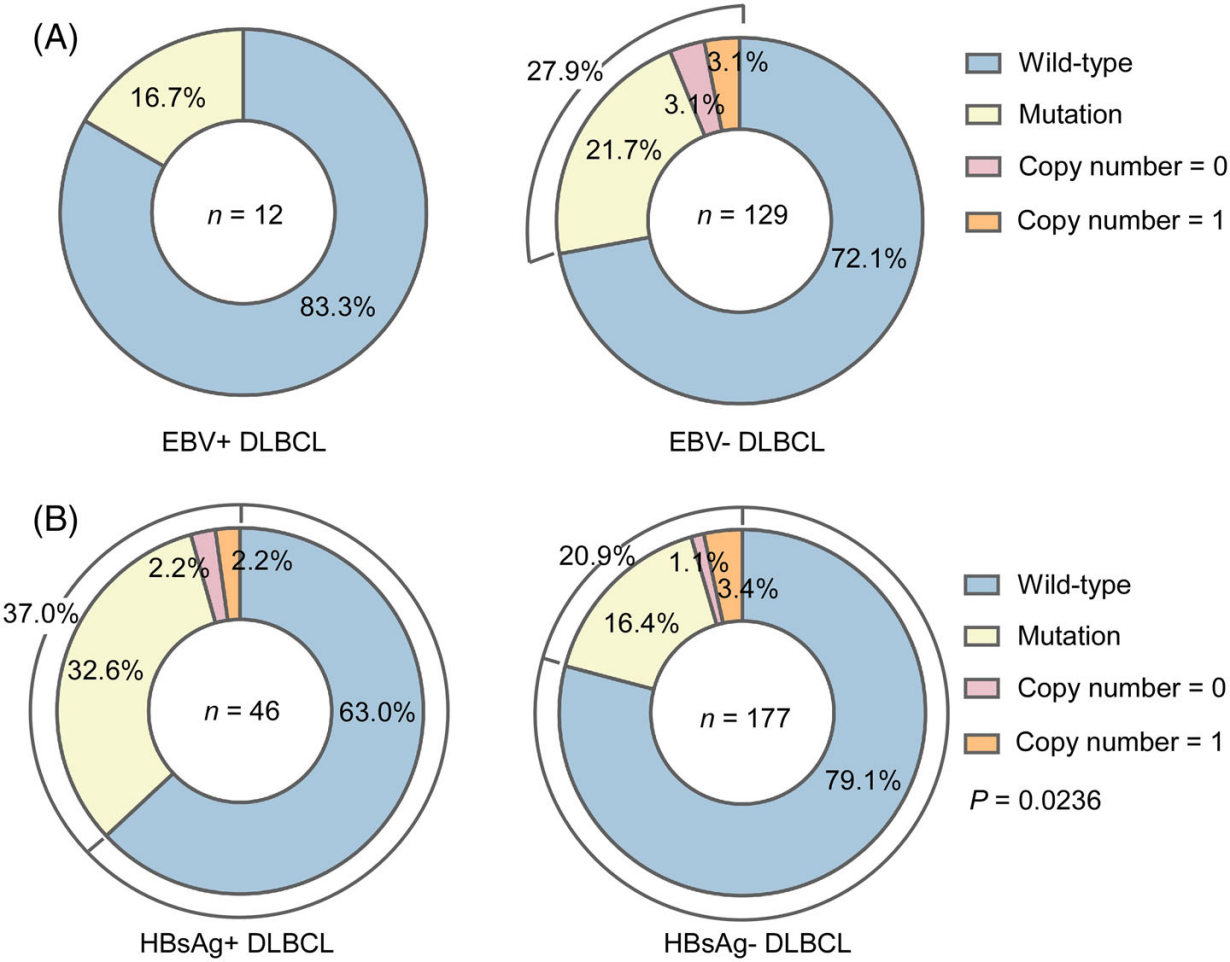
Hepatitis B virus (HBV) but not Epstein‒Barr virus (EBV) infection contributes to the increased *CD70* genetic alterations in Chinese diffuse large B‐cell lymphoma (DLBCL) patients. (A) *CD70* genetic aberration rates in tumour samples were similar in EBV‐positive and EBV‐negative DLBCL patients. (B) Compared with those from HBsAg‐negative DLBCL patients, tumour samples from HBsAg^+^ DLBCL patients were more frequently targeted by *CD70* genetic aberrations. χ^2^‐test, *P* = 0.0236.

Previous studies have suggested that infections caused by some viruses, such as HBV and EBV, are related to the development of DLBCL.^17^, ^37^ The latter has been recognised in the 2016 WHO classification as a separate entity, EBV^+^ DLBCL, not otherwise specified (NOS).^37^ CD70 is required for T‐cell‐mediated cytotoxic activity against EBV infections.^10^ In our study, a comparable EBV infection rate was noted between the Chinese (7.1%) and Swedish cohorts (9.3%; χ^2^ test, *P* = 0.43). Furthermore, no significant difference in the frequency of *CD70* genetic aberrations was observed between the EBV^+^ and EBV^−^ subgroups in the Chinese cohort (16.7% vs. 27.9%; χ^2^‐test, not significant; Figure 4A) or in the combined Chinese and Swedish cohort (13.3% vs. 36.5%, χ^2‐^test, not significant). Thus, EBV infection status was unlikely to be associated with the *CD70* mutations/CNV loss identified in DLBCL and could not explain the difference in the mutation frequency between the two populations. In contrast, approximately 20% of Chinese patients were HBsAg^+^, whereas no HBV infection was documented in the Swedish cohort. Furthermore, a significantly higher frequency of *CD70* mutation and/or deletion was observed in the HBsAg^+^ subgroup than in the HBsAg^−^ subgroup (37.0% vs. 20.9%, χ^2^‐test, *P* = 0.02; Figure 4B). Of note, HBsAg^−^ Chinese patients also displayed more *CD70* genetic aberrations than Swedish patients (20.9% vs. 10.8%; χ^2^‐test, *P <* 0.05; Figure S6). Thus, HBV infection contributed partially to the difference in the frequency of *CD70* genetic changes between Chinese and Swedish DLBCL samples and additional genetic and environmental factors might contribute to the observed difference.

### 3.4 CD70 genetic alterations are associated with a poor clinical outcome in patient subgroups

**FIGURE 5:**
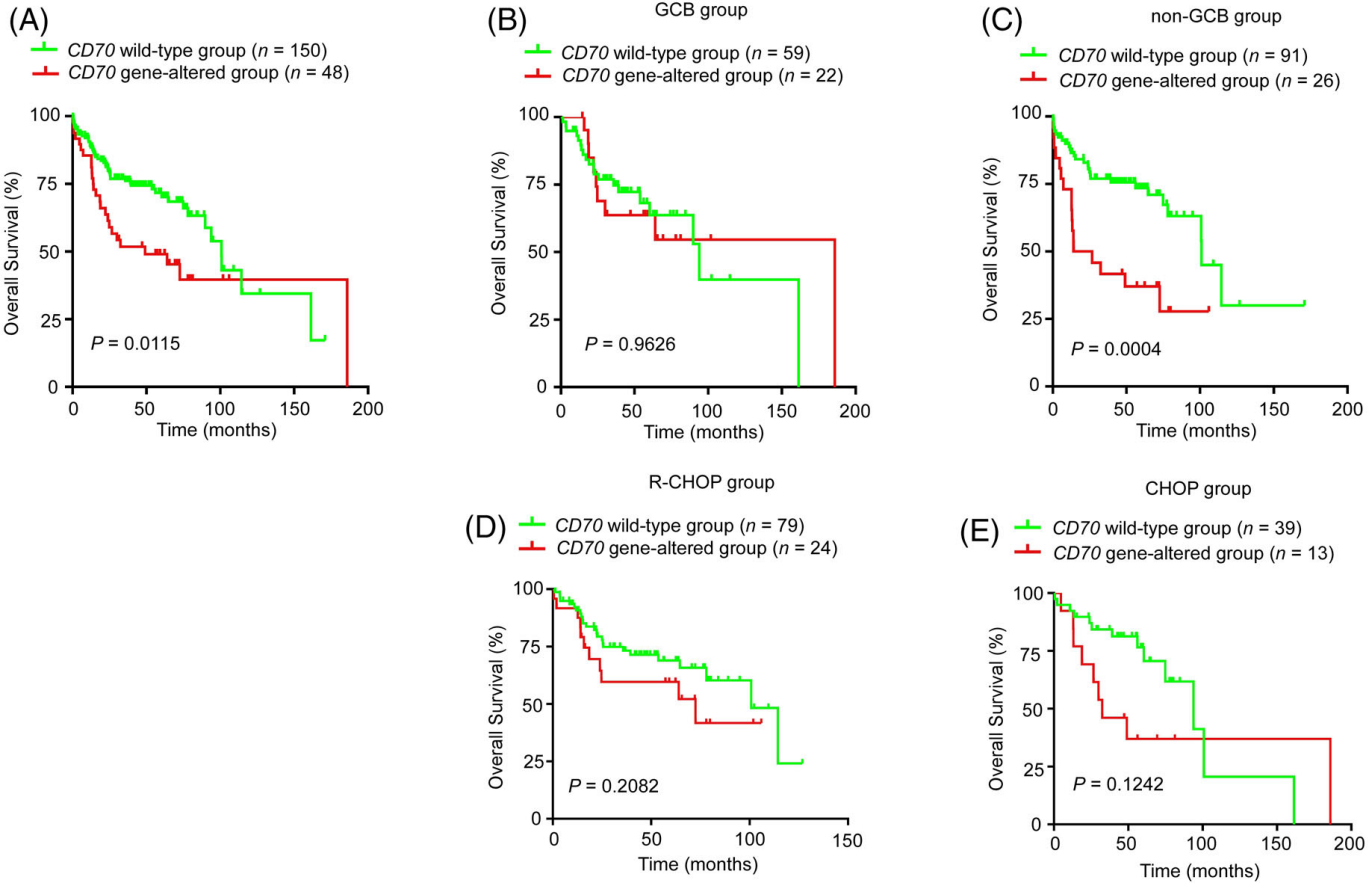
*CD70* genetic aberrations predict inferior overall survival in Chinese diffuse large B‐cell lymphoma (DLBCL) patients. (A) DLBCL patients with *CD70* genetic aberrations had a shorter OS than those with wild‐type *CD70*. The *P*‐value was calculated by the log‐rank test. HR (95% CI): 1.849 (1.139–3.002). (B–E) Kaplan–Meier survival curve of overall survival in the *CD70* wild‐type group and *CD70* gene‐altered group within the indicated cohorts. The *P*‐value was calculated by the log‐rank test. HR (95% CI): GCB: 0.980 (0.430–2.238); non‐GCB: 2.878 (1.564–5.294); R‐CHOP, 1.577 (0.771–3.224); CHOP, 1.988 (0.815–4.852).

Compared with the WT patients, the Chinese DLBCL patients with *CD70* genetic aberrations (mutations or copy number loss) displayed a significantly poorer performance status (Table S6a). Although no difference in the initial treatment response was observed between these two groups (CR+PR rate), a significantly shorter overall survival (OS) was observed in the patients in the *CD70* gene‐altered group and, more specifically, in those belonging to the non‐GCB subgroup (Figure 5A–C). Although a slightly poorer OS of the *CD70* gene‐altered group was observed both in the R‐CHOP‐ and CHOP‐treated groups, the difference was not significant due to the limited patient numbers (Figure 5D,E). Furthermore, in a multivariate analysis, CD70 genetic alteration was identified to be an independent predictor for inferior OS (HR, 1.778; 95% CI, 1.087–2.909; *P* = 0.022; Table S6b). Among Swedish DLBCL patients, no difference in clinical features was observed between the *CD70* WT and gene‐altered groups (Table S6c), although only a limited number of gene‐altered cases were available for analysis (*n* = 9).

### 3.5 The level of CD70 protein expression can also predict overall survival in diffuse large B‐cell lymphoma

**FIGURE 6:**
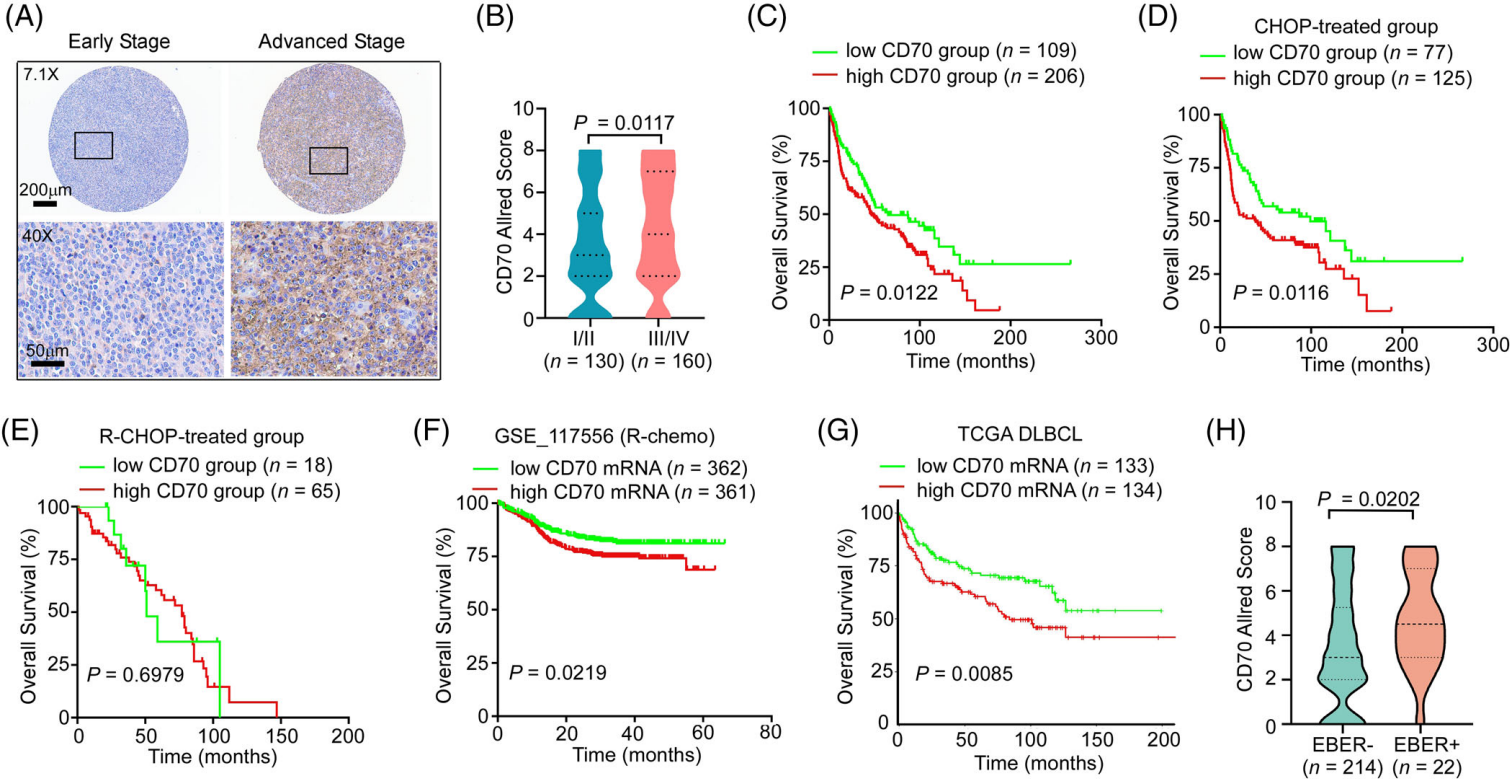
Higher CD70 expression is observed in advanced‐stage Swedish diffuse large B‐cell lymphoma (DLBCL) patients and correlates with inferior overall survival. (A) Representative images of CD70 staining in DLBCL patients with early‐ or advanced‐stage disease. (B) Higher CD70 protein expression was detected in DLBCL biopsies from patients with advanced‐stage disease. Early stage, median CD70 score = 3, range: 0–8; advanced stage, median CD70 score = 4, range: 0–8. Mann–Whitney *U*‐test, *P* = 0.0117. (C) Kaplan–Meier survival curve of overall survival in the low and high CD70 groups. The *P* value was determined by the log‐rank test. HR (95% CI): 1.472 (1.083–2.000). (D,E) The overall survival curves in the low and high CD70 groups within the CHOP‐treated (D) and R‐CHOP‐treated (E) DLBCL patients. The *P* value was determined by the log‐rank test. HR (95% CI): CHOP‐treated: 1.637 (1.121–2.392); R‐CHOP‐treated: 1.163 (0.538–2.515). (F) Kaplan–Meier survival curve of overall survival for DLBCL patients with high and low *CD70* mRNA expression in the GSE117556 dataset. The *P*‐value was determined by the log‐rank test, *P* = 0.0219. HR (95%CI): 1.453 (1.054–2.003). (G) Kaplan–Meier survival curve of overall survival for DLBCL patients with high and low *CD70* mRNA expression in the TCGA DLBCL database. The data were generated by OSdlbcl online consensus survival analysis web server. The *P*‐value was determined by the log‐rank test, *P* = 0.0085. (H) Higher CD70 protein expression was detected in the EBV^+^ DLBCL, not otherwise specified (NOS). Mann–Whitney *U*‐test, *P* = 0.0202.

As ectopic or aberrant expression of CD70 is potentially associated with clinical courses in several types of cancer,^19^ we next studied the value of CD70 protein expression for predicting survival. As higher frequency of CD70 mutations were found in the Chinese cohort (which resulted in reduced protein expression), we focused our analysis on the Swedish cohort (with fewer *CD70* genetic alterations), where we also have access to a large number of tissue samples for protein analysis (*n* = 315, preprinted on tissue arrays). Notably, higher CD70 expression on tumour cells was observed in the patients diagnosed with a more advanced stage of disease (Figure 6A,B). Furthermore, when patients were grouped based on the level of CD70 protein expression (score 0–2 vs. 3–8, referred to as low CD70 vs. high CD70 groups, Table S7), a significantly higher IPI, higher LDH level, and poorer treatment response were observed in the high CD70 group. Moreover, a significantly poorer OS was noted in patients with high CD70 protein expression treated with the CHOP regimen but not the R‐CHOP regimen (Figure 6C–E). The impact of CD70 expression on DLBCL survival was further explored in the gene expression omnibus (GEO) and TCGA databases. High *CD70* mRNA expression was also associated with shorter OS in a rituximab‐containing regimen‐treated DLBCL cohort (GSE117556)^38^ and TCGA DLBCL cohort (dbGaP Study Accession: phs000178^39^; figure generated by online OSDLBCL tool^40^) (Figure 6F,G).

Latent EBV infection has been shown to induce the expression of CD70 on host cells, including lymphoma cells.^41^ A significantly higher EBV infection rate was indeed observed in the high CD70 group (Table S7), and EBER^+^ cases had significantly higher CD70 protein expression scores (Figure 6H), regardless of age at diagnosis (data not shown). Taken together, these results indicate that higher expression of the CD70 protein is associated with a more advanced stage of disease and EBV infection status and that gene or protein expression can also predict poorer long‐term survival.

It seems paradoxical that both *CD70* genetic alteration (mutations and/deletions that result in reduced or absent CD70 protein expression) and higher levels of CD70 protein expression are associated with poorer survival. Among the Chinese patients with available genetic, protein staining and clinical data, we next directly compared the OS of three groups of patients: those with *CD70* genetic aberration (*CD70* gene‐altered group), those with *CD70* WT sequences and low CD70 or high CD70 protein expression (referred to as low or high CD70 groups). Although the sample size was very small, we observed a trend that the *CD70* gene‐altered group and high CD70 group presented with a similar shorter OS than the low CD70 group (Figure S7A). In the *CD70* gene‐altered group, a set of genes including *BTG2, KLF2, BTG1, SOCS1, BCL6, DTX1, B2M, TNFRSF14* and *CXCR4* were mutated more frequently (Figure S7B). In the high CD70 group, *SGK1, ROBO1* and *VMP1* were mutated at higher levels (Figure S7B). Furthermore, the MHC‐II‐related pathway was significantly upregulated in the *CD70* gene‐altered group (Figure S7C). Ecotypes were recently described to characterise the tumour microenvironment of DLBCL.^28^ Nine lymphoma ecotypes (LE) were detected in both groups (Figure S7D). The *CD70* gene‐altered group had a higher frequency of LE7, which was enriched with infiltrating B cells, mast cells, NK cells, endothelial cells, DCs and T follicular helper cells (Figure S7E). The high CD70 group had a higher frequency of LE4 (Figure S7E), which was characterised by immunoactive‐T‐cell states with widespread expression of coinhibitory and stimulatory molecules.^28^ Taken together, patients in the *CD70* gene‐altered group and high CD70 group had similar poor long‐term survival, but their tumours exhibited distinct molecular signatures, suggesting different underlying mechanisms.

### 3.6 Single‐cell RNA sequencing revealed T‐cell exhaustion in diffuse large B‐cell lymphoma expressing high levels of CD70

**FIGURE 7:**
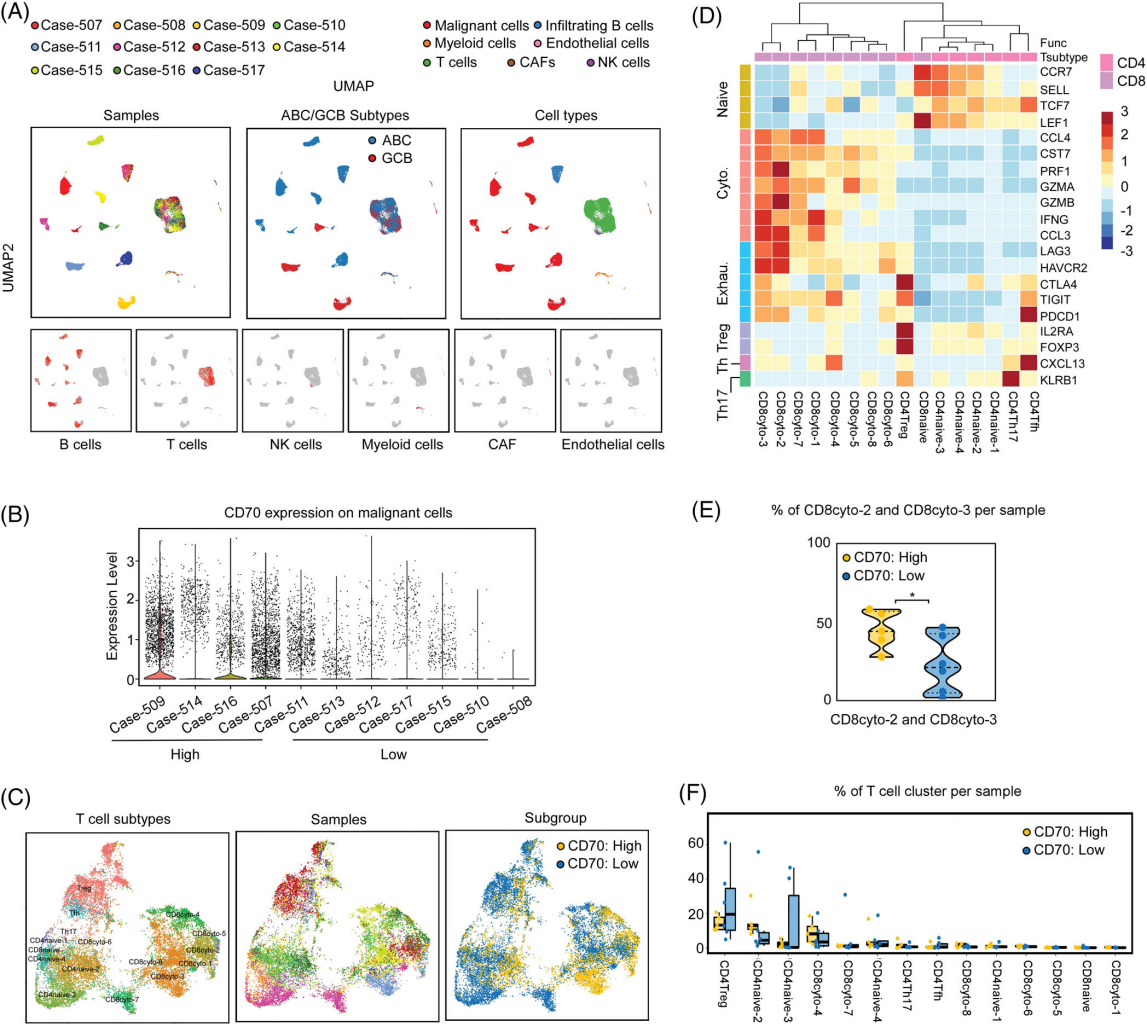
Single‐cell RNA sequencing revealed T‐cell exhaustion in diffuse large B‐cell lymphoma (DLBCL) with high levels of CD70. (A) Single‐cell RNA sequencing analyses of 11 DLBCL samples. Uniform manifold approximation and projection (UAMP) plot showing the sample origins and DLBCL subtypes of each cell cluster (left and middle panels). Clusters were assigned to the indicated cell types by canonical markers (right panel). CAFs, cancer‐associated fibroblasts. NK cells, natural killer cells. (B) Quantification of CD70 on the malignant B cells of each sample. Samples were grouped (low and high) based on the CD70 expression on malignant B‐cells. (C) UAMP plot showing T‐cell subclusters (left panel). Sample origins are indicated in the middle panel. Subclusters were assigned to CD70 high and low groups based on the CD70 expression on the corresponding malignant B cells (right panel). (D) Expression of functional status markers of T cells. (E) The percentages of CD8cyto‐2 and CD8cyto‐3 cells per sample are shown. *P = 0.0495, one‐tailed Mann–Whitney *U*‐test. (F) The percentages of T cells per sample are shown for the indicated subcluster. Not significant, one‐tailed Mann–Whitney *U*‐test.

To comprehensively explore the tumour microenvironment in DLBCLs expressing CD70, we reanalysed our recently published single‐cell RNA sequencing (scRNA‐seq) data on an independent Chinese DLBCL cohort.^42^ Altogether, 55 405 cells from 11 DLBCL, NOS, were included in the reanalysis, and no CD70 genetic aberrations were detected by WGS of the matched bulk tissue. The major cell types were determined by the expression of canonical markers and the malignant B cells were furthermore identified based on inferred CNVs.^42^ In total, 30 159 malignant B cells (54.4%), 4 588 non‐malignant B cells (8.3%), 18 244 T cells (32.9%), 1 255 myeloid cells (2.3%), 476 natural killer (NK) cells (0.9%), 355 cancer‐associated fibroblasts (CAFs) (0.6%) and 328 endothelial cells (0.6%) were identified (Figure 7A, Table S8). Based on the quantification of *CD70* mRNA expression on tumour cells, we divided patients into high‐expression and low‐expression groups (Figure 7B).

A total of 16 CD4^+^ or CD8^+^ tumour‐infiltrating T‐cell subclusters were identified in all samples. Using canonical markers, these T‐cell subclusters were further assigned as naive T cells, cytotoxic T cells, regulatory T cells, T follicular helper (Tfh) cells and T helper 17 cells (Th17) (Figure 7C,D). We then estimated the exhaustion status of cytotoxic T cells based on the expression level of the key coinhibitory receptors (*LAG3*, *HAVCR2*, *CTLA4*, *TIGIT* and *PDCD1*). These T‐cell exhaustion marker genes were expressed at much higher levels in the CD8cyto‐2 and CD8cyto‐3 subclusters (Figure 7D). Interestingly, the percentage of these two subclusters per sample in the CD70 high‐expression group was significantly higher than that in the CD70 low‐expression group (Figure 7E). No significant difference in the percentage of other T‐cell subclusters per sample was observed between the two groups (Figure 7F). Together, the scRNA‐seq data suggest that the tumour microenvironment in CD70‐high expression DLBCL seems to be more immunosuppressive, characterised with T‐cell exhaustion.

**FIGURE 8:**
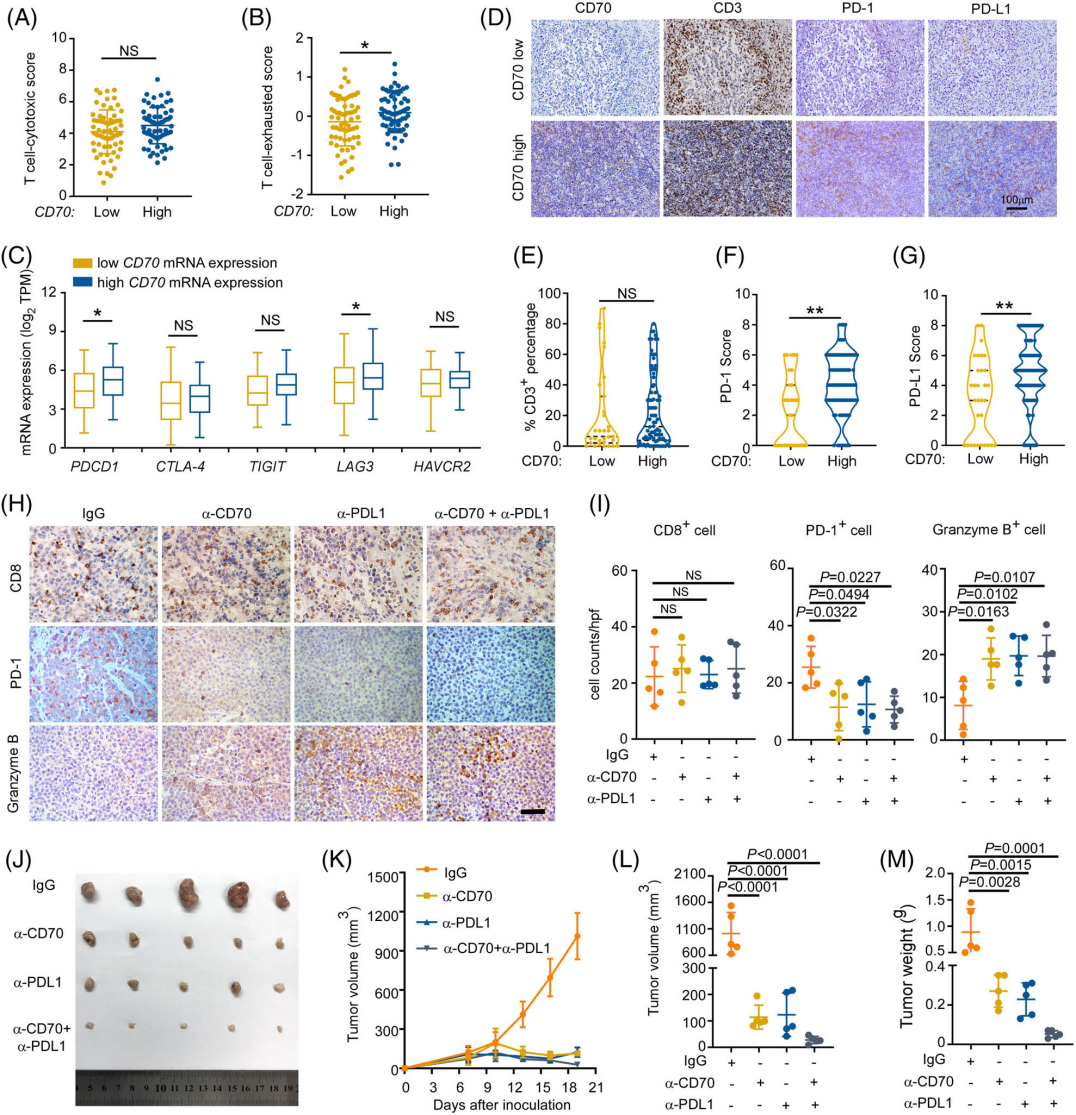
CD70/CD27 and PD‐1/PD‐L1 coinhibition rescues T‐cell exhaustion and reduces lymphoma growth in vivo. (A,B) T‐cell‐cytotoxic score and T‐cell‐exhausted score (calculated based on transcriptomic data) for diffuse large B‐cell lymphoma (DLBCL) patients with low (*n* = 64) and high (*n* = 63) *CD70* mRNA expression, Student's *t*‐test; ns, not significant. (C) The mRNA expression of *PDCD1*, *CTLA‐4*, *TIGIT*, *LAG3* and *HAVCR2* in DLBCL patients with low (*n* = 64) and high (*n* = 63) *CD70* mRNA expression; the fold changes in the mRNA expression of *PDCD1* and *LAG3* were 1.9 and 1.3, respectively. Student's *t*‐test, *PDCD1: P* = 0.0242; *LAG3*: *P* = 0.0223; ns, not significant. (D) Representative immunohistochemical (IHC) images of CD70, CD3, PD‐1 and PD‐L1 in DLBCL patients. Original magnification, 20×; scale bar, 100 μm. (E) CD3^+^ T‐cell infiltration was similar between the different CD70 expression groups. *n* = 115. Student's *t*‐test; NS, not significant. (F) PD‐1 expression was higher in the high CD70 group. *n* = 115. Mann–Whitney *U*‐test, **P = 0.0004. (G) PD‐L1 expression was higher in the high CD70 group. *n* = 114. Mann–Whitney *U*‐test, **P = 0.0030. (H–M) A20 lymphoma cell‐bearing BALB/c mice were treated with isotype control antibody or the indicated blocking antibody every three days, starting 7 days after inoculation. *n* = 5 mice/group. (H) Representative IHC staining for CD8, PD‐1, and granzyme B in tumour tissues from the indicated treated groups. Original magnification, 40×; scale bar, 50 μm. (I) CD8^+^ T‐cell counts (left panel), PD‐1^+^ (middle panel) and granzyme B^+^ cell (right panel) numbers under a 40× field for the indicated groups. One‐way ANOVA with Tukey's test; NS, not significant. (J) When the mice were sacrificed, the tumours were harvested. (K,L) Tumour volumes were monitored every 3 days and calculated as the length×width^2^×0.5. One‐way ANOVA with Tukey's test. (M) The tumour weight in the indicated groups. One‐way ANOVA with Tukey's test.

To further validate the scRNA‐seq data, we evaluated the cytotoxic and exhausted T‐cell scores based on transcriptome analysis of *CD70* WT Chinese DLBCL bulk tissue samples (*n* = 127). Although no difference in the T‐cell‐cytotoxic score was detected between the *CD70* mRNA‐high and low groups (Figure 8A), a higher T‐cell‐exhausted score was observed in the CD70 mRNA‐high group (Figure 8B), along with more *PDCD1* and *LAG3* expression (Figure 8C). We further analysed the protein expression of the T‐cell surface marker CD3 as well as PD‐1 and PD‐L1 in Swedish DLBCL samples by IHC. We observed that although the number of infiltrated CD3^+^ T cells was similar in the different groups (Figure 8D,E), higher levels of PD‐1 and PD‐L1 protein expression were detected in samples with positive CD70 protein expression (Figure 8F,G). Together, these data suggested that high CD70 mRNA/protein expression in DLBCL may lead to the exhaustion of infiltrated T cells.

### 3.7 CD70/CD27 and PD‐1/PD‐L1 coinhibition rescues T‐cell exhaustion and reduces lymphoma growth in vivo

We next tested the hypothesis that coinhibition of CD70 and PD‐L1 could rescue exhausted T cells and effectively reduce lymphoma growth in vivo. The murine DLBCL cell line A20, which highly expresses CD70,^43^ was inoculated into the inguinal lymph node region of BALB/c mice. After 7 days of engraftment, tumour‐bearing mice were randomised to receive IgG, anti‐CD70 treatment (αCD70) alone, anti‐PD‐L1 treatment (αPD‐L1) alone, or αCD70/αPD‐L1 cotreatment for 2 weeks. Although the infiltration of CD8^+^ T cells was similar among these groups (Figure 8H,I), PD‐1 expression was significantly lower in the αCD70 and/or αPD‐L1 treatment groups (Figure 8I). As a marker of T‐cell activation, granzyme B was also analysed by IHC and was increased in the αCD70 and/or αPD‐L1 treatment groups (Figure 8I). Furthermore, compared with IgG treatment, inhibition of CD70 and PD‐L1 resulted in significant growth retardation of tumours (Figure 8J–M). Significantly weaker expression of KI‐67 was also observed in the anti‐CD70‐treated groups, indicating slower cell proliferation (Figure S8). Our results thus suggested that dual blockade of the CD70/CD27 and PD‐1/PD‐L1 pathways could hamper lymphoma growth in vivo, at least partially through rescue of T‐cell exhaustion.

## 4 DISCUSSIONS

CD70 is an emerging target in cancer immunotherapy. However, the tumourigenic role of CD70 in cancer, especially in lymphoid malignancies, remains elusive. Here, after a comprehensive genomic characterisation of the tumour samples from DLBCL patients in two populations, we observed a strikingly high frequency (24.0%) of gene aberrations in *CD70* in the Chinese cohort. Furthermore, we showed for the first time that these genomic changes, including both somatic mutations and copy number loss, were loss‐of‐expression and/or loss‐of‐function and were associated with inferior long‐term survival among patients with the more aggressive non‐GCB subtype of disease. *CD70* mutations are enriched in the C1 molecular subtype of DLBCL,^5^ which is characterised by *BCL6* translocation and *NOTCH2* mutations. The majority of C1 DLBCLs were classified as ABC‐type tumours and were associated with better survival than those belonging to the other ABC‐dominant molecular subtype, C5.^5^ Notably, this predictive value was based on the gene signature of C1, which includes combined changes in many genes, including *CD70*, and the treatment regimen evaluated was R‐CHOP. *CD70* mutations were also frequently identified in the GCB‐dominant C4 subtype.^5^ Indeed, *CD70* genetic changes identified in our patient cohorts did not show any significant preference for the GCB or non‐GCB subtype; instead, they were enriched in HBV^+^ DLBCL patients. Thus, chronic viral infections, genetic backgrounds and treatment strategies may all affect the clinical relevance of *CD70* mutations.

We further demonstrated that the higher CD70 protein expression was associated with EBV infection and a late disease stage, and the gene or protein expression may predict poorer long‐term survival in a subgroup of patients. Our study thus suggests that both loss of expression/function of CD70 and constitutive expression of CD70 can play a pathological role in DLBCL disease progression, which is consistent with the tight regulation of this immune regulator under physiological conditions.

We previously reported that the CD27–CD70 interaction is responsible for the initial priming of antigen‐specific T cells and that children born with CD70 germline mutations are at increased risk for the development of EBV‐driven B‐cell lymphoma, probably due to the impaired antiviral functions of CD70‐deficient T cells.^10^ DLBCL malignant B cells possess the properties of professional antigen‐presenting cells and are involved in the priming phase of tumour‐specific T cells.^14^ Loss of expression of the costimulator CD70 on malignant B cells due to somatically occurring genetic alterations may thus result in an inability to interact with CD27‐expressing T cells during the antigen presentation and priming phase, which may explain why *CD70* genetic changes are more specific for B‐cell malignancies such as DLBCL and follicular lymphoma^44^ and are extremely rare in other types of cancer. This finding is further supported by our recent single‐cell RNA‐seq analysis in DLBCL, where a strong CD70–CD27 costimulatory interaction was observed between malignant B cells and infiltrated T cells.^42^

In the CD70‐overexpressing cases, the “immunogenic” signals provided by the CD70/CD27 interaction might have turned into “tumourigenic” signals. It has been shown that constitutive expression of CD70 on T cells can induce waning of memory CD8^+^ T cells against viruses and exhaustion of CD4^+^ effector memory T cells.^45^, ^46^ Furthermore, CD70^+^ lymphoma B cells have been shown to partially contribute to Foxp3 expression in intratumoural CD4^+^CD25^−^ T cells and thus the associated regulatory activity.^20^ The constitutive binding of CD70–CD27 not only can induce the overexpression of PD‐1 but may also upregulate the expression of Fas ligand and BCL‐XL.^47^ Our clinical data, scRNA‐seq analysis and murine lymphoma model clearly showed that high CD70 expression might be associated with an increased number of exhausted T cells and thus a more immunosuppressive TME. Furthermore, a recent study showed that T cells enriched in ABC–DLBCL exhibited overexpression of immunomodulatory molecules including CD27, but T cells associated with GCB–DLBCL were generally deficient in these molecules.^28^ This may partially explain why CD70–CD27 may play more important roles in the ABC–DLBCL rather than the GCB–DLBCL. Other co‐stimulatory molecules like inducible T cell co‐stimulator ligand (ICOSL) may also play a dual role in the tumour develpment.^48^ Although the binding of ICOS and ICOSL is essential for the activation and function of T cells,^49^ a recent study found that overexpression of ICOSL on AML cells promoted the expansion of regulatory T cells and predicted poorer survival in those patients.^50^ The aberrant expression of ICOSL was also associated with poor prognosis in invasive breast cancer.^51^

These distinct immune escape mechanisms related to CD70 dysregulation highlight the necessity of developing a precise or more targeted immunotherapy for B‐cell malignancies. When immune evasion results from *CD70* genetic alterations, restoring adequate T‐cell priming may augment tumour immunity. Preclinical studies have also suggested that agonistic mAbs targeting CD27 promote CD8^+^ T‐cell‐dependent tumour rejection and reduce the frequency of Tregs in a murine tumour model.^52^ A phase I evaluation of an agonistic anti‐CD27 human antibody (varlilumab) showed safety and tolerability in advanced solid cancers, and a reduction in Tregs was also observed in the peripheral blood of patients (NCT01460134).^53^ A phase II trial of varlilumab plus nivolumab is ongoing in relapsed/refractory aggressive B‐cell lymphomas (NCT03038672). Enhancing other costimulatory signals, such as OX‐40 agonists and 4‐1BB agonistic antibodies,^54^, ^55^ may also overcome the defect in CD70 and further studies may shed light on these novel strategies. Finally, a PD‐1–CD70 fusion protein has recently been described that can target tumour cells by blocking the PD‐1–PDL1 interaction and provide the missing CD70 activation signal to CD27^+^ T cells.^56^ When immune evasion is induced by dysregulated CD70/CD27 interactions, the blockade of CD70/CD27 signalling has the potential to rescue the exhausted TME in CD70^+^ DLBCL. Several CD70‐blocking antibodies are being tested in clinical trials (EudraCT number 2012‐005046‐38, NCT00944905, NCT02216890).^57^, ^58^, ^59^ However, only modest single‐agent activity was observed in heavily treated DLBCL (NCT02216890).^59^ Based on our results, combined therapy with PD‐L1 blockade and anti‐CD70 mAb is likely to be a promising therapeutic strategy in CD70^+^ DLBCL. In addition, aberrant expression of CD70 has also been reported in other cancers, such as renal cancer,^60^ melanoma^61^ and AML.^22^ This further broadens the utility of CD70/CD27 axis blockade, and a CD70‐specific CAR T cell strategy has also recently been tested for gliomas.^19^

Although both HBV and EBV have been reported to be involved in the development of DLBCL,^17^, ^37^ they may play distinct roles. Genetic evidence in this study demonstrated an enrichment of *CD70* alterations in HBV‐associated DLBCL. We have previously reported that the majority of genes that are highly mutated in HBV‐associated DLBCL, including *CD70*, are potential targets of activation‐induced (cytidine) deaminase (AID).^17^ We have also shown an increased number of mutations associated with the APOBEC signature in this DLBCL subtype.^17^ Thus, the increased targeting of CD70 in HBV‐associated DLBCL may be explained by the higher activities of the APOBEC enzyme and/or the B‐cell specific factor AID. Of note, a high burden of HBV infection is observed not only in China but also in South Asia, sub‐Saharan Africa, and some countries in the Western Pacific region.^62^ DLBCL patients in these areas are more likely to suffer from HBV infection and harbour a higher mutation burden, including in *CD70*, and they may benefit from therapies that restore CD70/CD27 signalling.

Unlike HBV, EBV infection was not associated with *CD70* mutations or CNV loss but with increased protein expression of CD70. It has been shown that the EBV oncoprotein latent membrane protein 1 (LMP1) upregulates CD70 expression in EBV‐infected epithelial and B cells,^41^ and LMP1 may thus also contribute to the overexpression of CD70 in other EBV‐induced malignancies, such as nasopharyngeal carcinoma, Burkitt's lymphoma, and nasal NK/T‐cell lymphoma.^23^ EBV^+^ DLBCL patients show a poorer response to the current standard R‐CHOP regimen and shorter survival.^37^ Anti‐CD70 treatment and combined therapy with immune checkpoint inhibitors may thus have potential for EBV^+^ DLBCL and possibly also other EBV‐associated, CD70‐expressing malignancies.

### 4.1 Limitations of the study

Despite performing a comprehensive analysis of CD70 in DLBCL at the genetic and protein levels as well as in single‐cell solutions, the number of patients analysed in the study is still limited. In addition, the correlation of CD70 genetic aberrations and protein overexpression with poor survival was mainly generated in two different cohorts. Further analysis of a larger cohort will be needed to validate our results. Finally, genetic inhibition of CD70 in the mouse model will help to further evaluate the tumour microenvironment in vivo.

## 5 CONCLUSIONS

Our findings suggest that dysregulation of CD70 contributes to the pathogenesis of DLBCL; both lack of expression/function due to mutations/deletions in the *CD70* gene and constitutive expression of CD70 might led to immune evasion but via distinct mechanisms. This dual role of CD70 in B‐cell lymphomagenesis, suppression, and oncogenesis also emphasises the importance of characterising the specific defects/dysfunctions in CD70 and developing therapeutic strategies accordingly.

## AUTHORSHIP CONTRIBUTIONS

M. N. performed the experiments, analyzed and interpreted the data, and wrote the manuscript; W. R. and X. Y. analyzed and interpreted the sequencing data; M. B. prepared samples, collected clinical information, and analyzed the staining data; R. A supervised the IHC analysis; L. D., Y. G. and W.S. performed the FACS assay; D. L. performed the animal experiment; K. F., W. L., J. L., X. W., and G. E. prepared samples and collected clinical information; C. J and H. B. analyzed the staining data; D. L., and X. Y. performed the bioinformatics analysis; W. J., H. H., Y. H., S. Z., G. E., M. J., H. Z., K. W., Z. L., and R.‐M. A. were involved in the supervision of the study; and Q. P.‐H. designed and supervised the study and wrote the manuscript.

## CONFLICTS OF INTEREST

The authors declare that there is no conflict of interest that could be perceived as prejudicing the impartiality of the research reported.

## Supporting information

Supporting Information

Supporting Information

Supporting Information

Supporting Information

Supporting Information

Supporting Information

Supporting Information

Supporting Information

Supporting Information
