# Supplementary material for: The dual role of CD70 in B‐cell lymphomagenesis
Source: Clin Transl Med. 2022 Dec 5;12(12):e1118. doi: 10.1002/ctm2.1118 (PMC9722974; doi:10.1002/ctm2.1118)
Supplement: Supplementary file 1 — Supporting Information [file CTM2-12-e1118-s008.docx]

**Supplementary Figures**
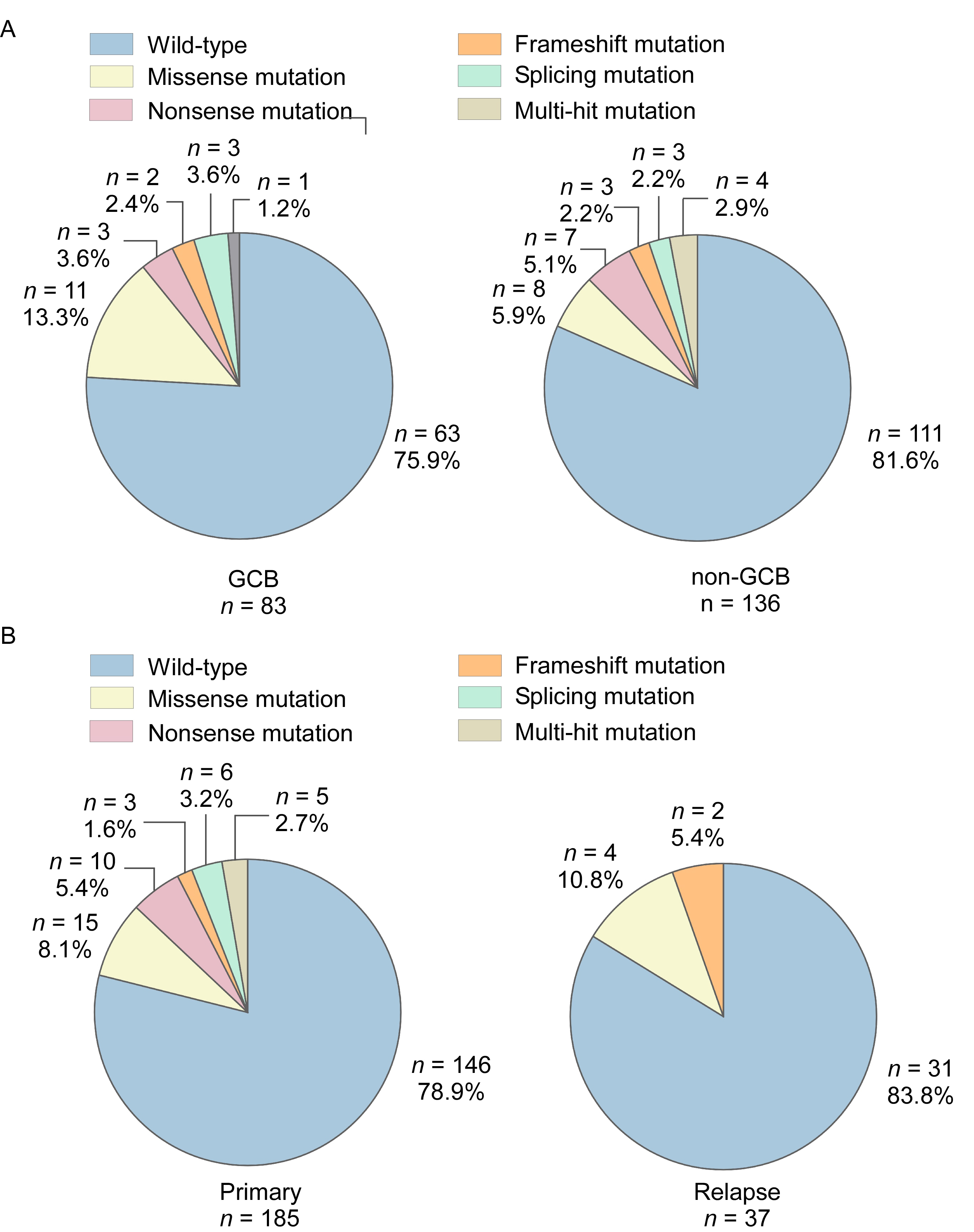


**Supplementary Figure 1.** Genetic aberrations of *CD70* in different DLBCL subtypes.

1. The proportion of *CD70*-mutated samples was similar in the GCB and non-GCB subtypes. χ^2^ test, not significant.
2. The proportion of *CD70*-mutated samples was similar in the primary and relapsed DLBCL groups. χ^2^ test, not significant.


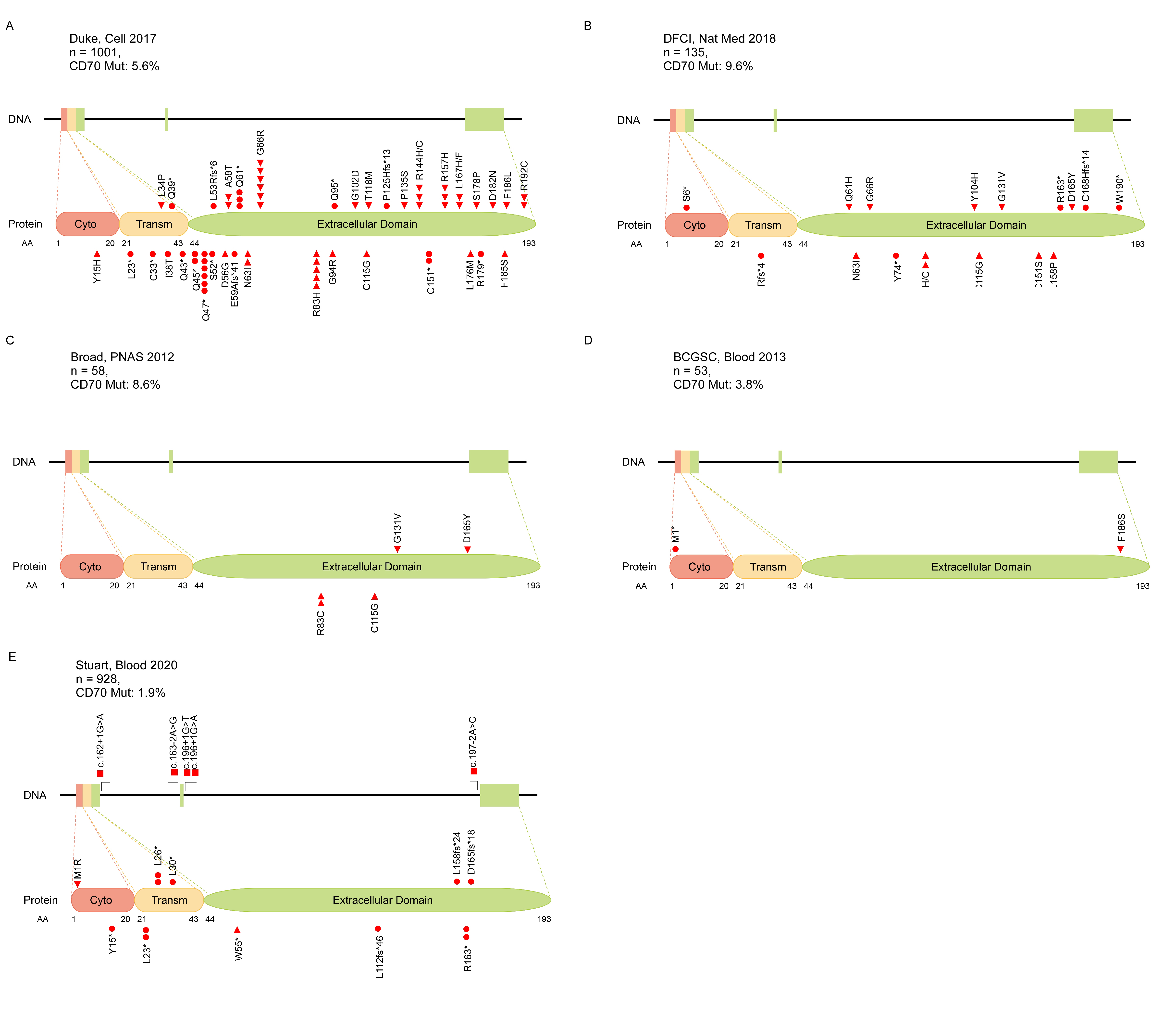


**Supplementary Figure 2.** The distribution of *CD70* mutations was summarized from different DLBCL cohorts published previously.

A-E. The position of *CD70* mutations identified from different DLBCL cohorts^1-5^ was visualized.

**
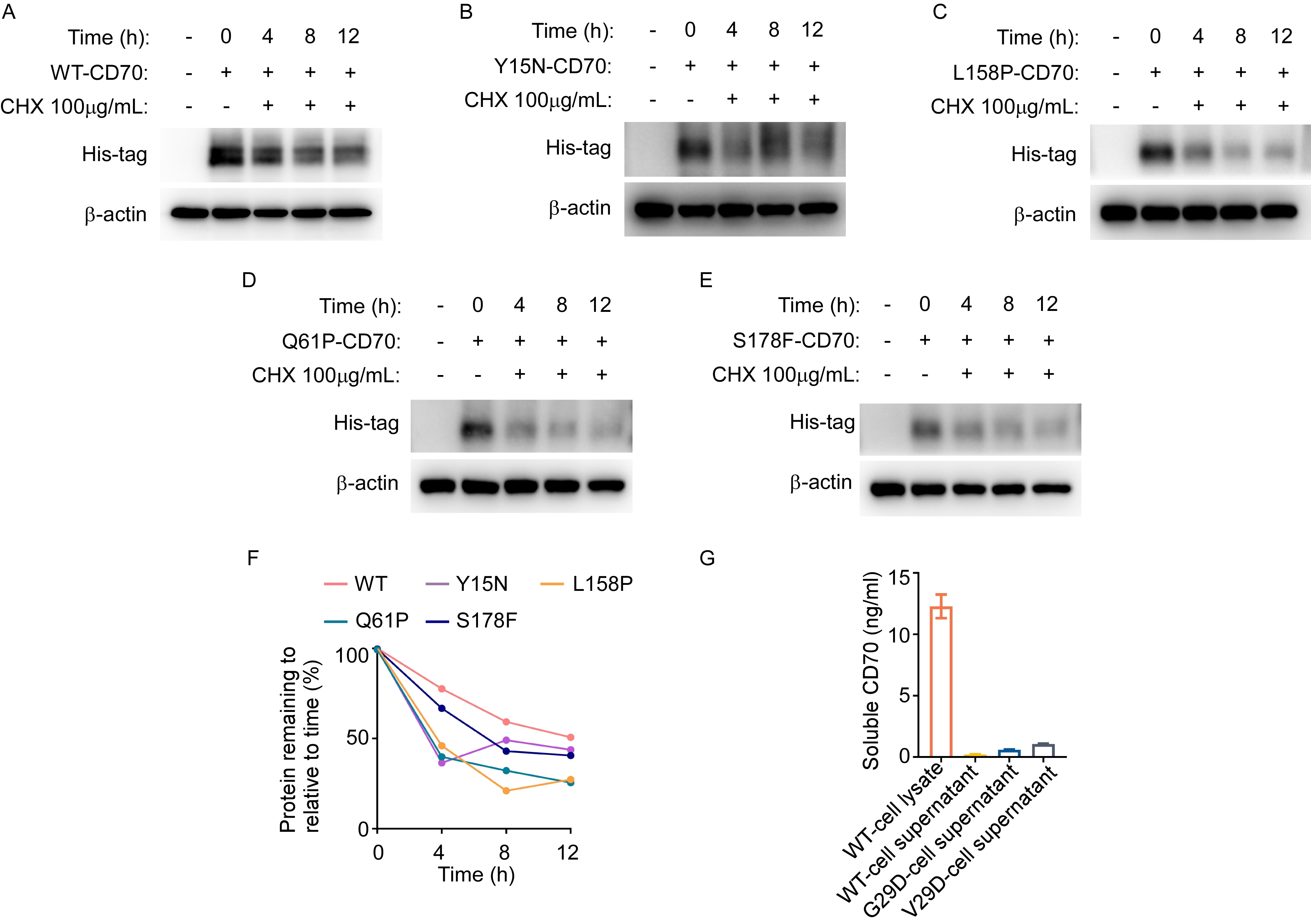
**

**Supplementary Figure 3.** *CD70* genetic alterations resulted in a reduction/loss of protein expression *in vitro*.

HEK293T cells were either not transfected (−) or transiently transfected (+) with 1 μg *CD70* WT or the indicated mutant plasmids.

(A-E). Twenty-four hours posttransfection, cells were either left untreated (−) or treated with translational inhibitor cycloheximide CHX (+) (100 μg/ml). Then cells were collected and analyzed from the indicated time points.

F. Tagged protein expression of His is quantified and shown as a relative value to the starting point (0). Band intensities were quantified using ImageJ software.

G. The cell supernatants were harvested at 24 h after transfection of indicated plasmids and filtered with a 0.45 μm syringe filter. The cell lysate of *CD70* WT transfected cells was used as a positive control. Each group contains two replicates. Soluble CD70 was detected by a commercial ELISA kit (Abcam, ab264621).

**
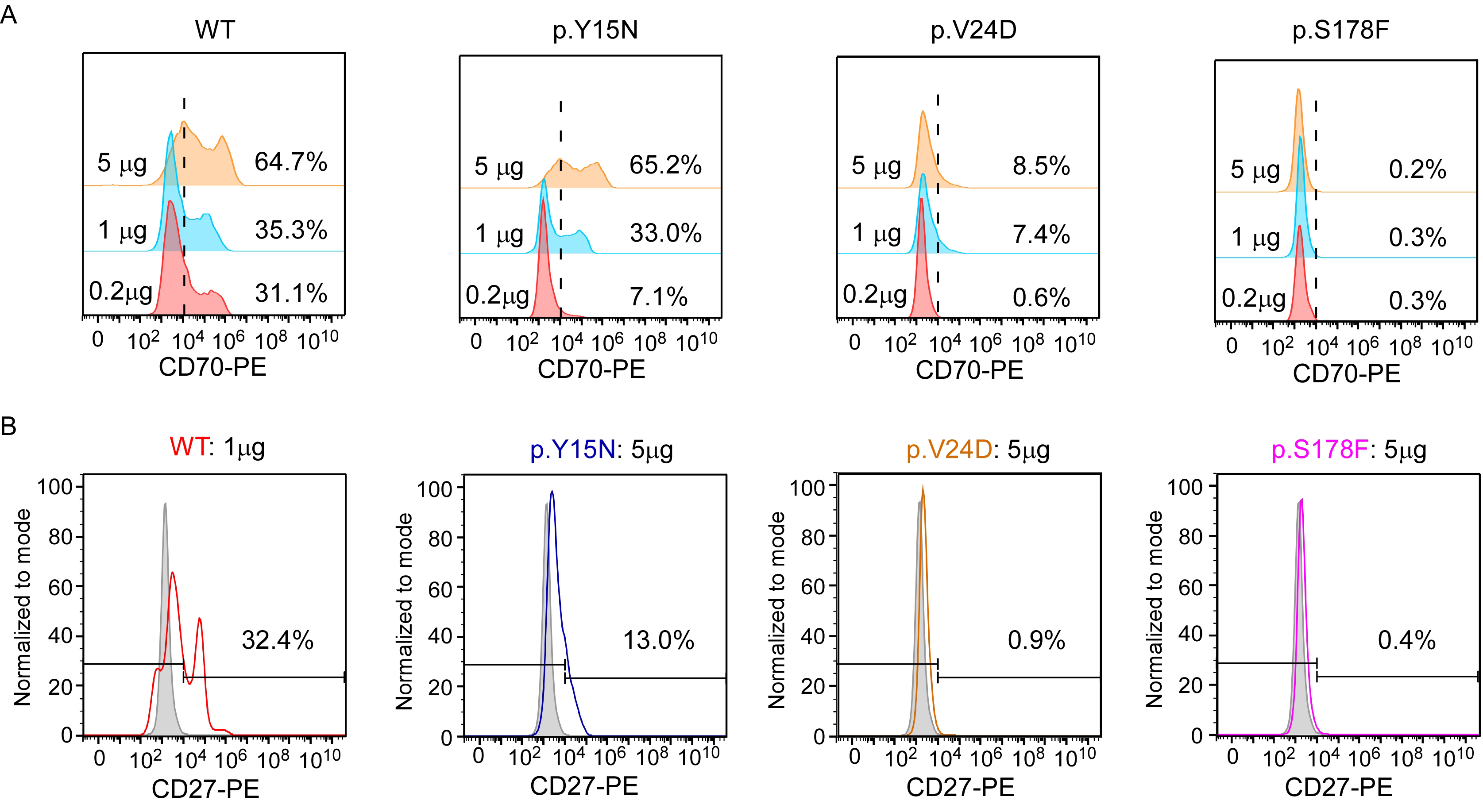
**

**Supplementary Figure 4.** *CD70* genetic alterations resulted in a reduction/loss of CD27 binding *in vitro*.

A. HEK293T cells were transfected with 0.2 μg, 1 μg and 5 μg of indicated plasmids, and then CD70 expression was detected by flow cytometry.

B. HEK293T cells were transfected with 1 μg of *CD70* WT or 5 μg (overexpressed) of the indicated mutant plasmids, and the binding activity of WT/mutant *CD70* and recombinant human CD27 was measured by flow cytometry. The shaded area represents nontransfected HEK293T cells.

**
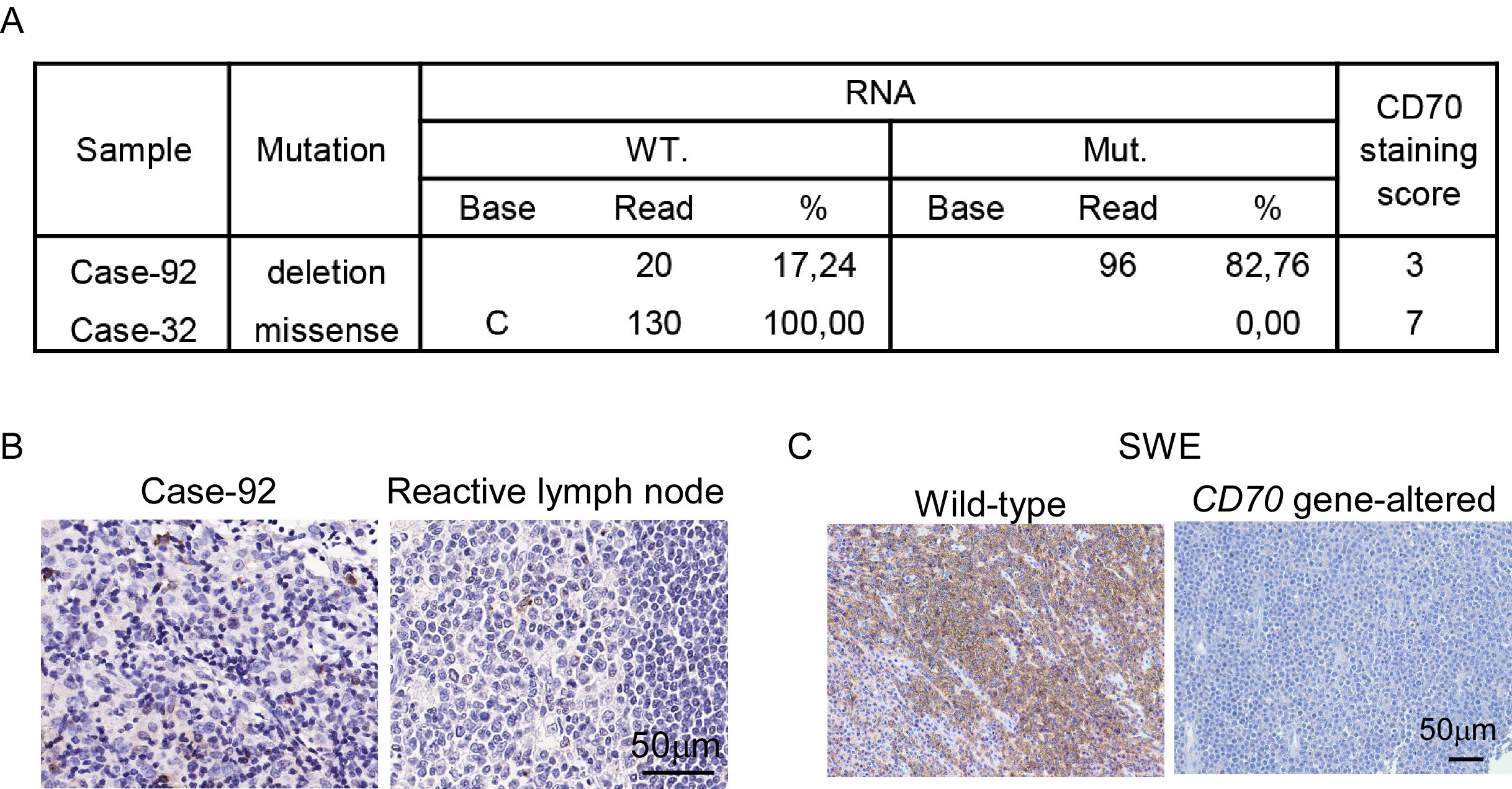
**

**Supplementary Figure 5.** CD70 protein expression is associated with *CD70* genetic status in DLBCLs.

A. The table shows the proportion of *CD70* WT/mutant alleles and CD70 IHC staining in matched tumor samples harboring *CD70* mutations.

B. IHC images shows the CD70 staining in Case-92 and the reactive lymph node.

C. Representative IHC images show the CD70 staining in Swedish DLBCL patients (genetic status as indicated). The scale bar represents 50 µm.

**
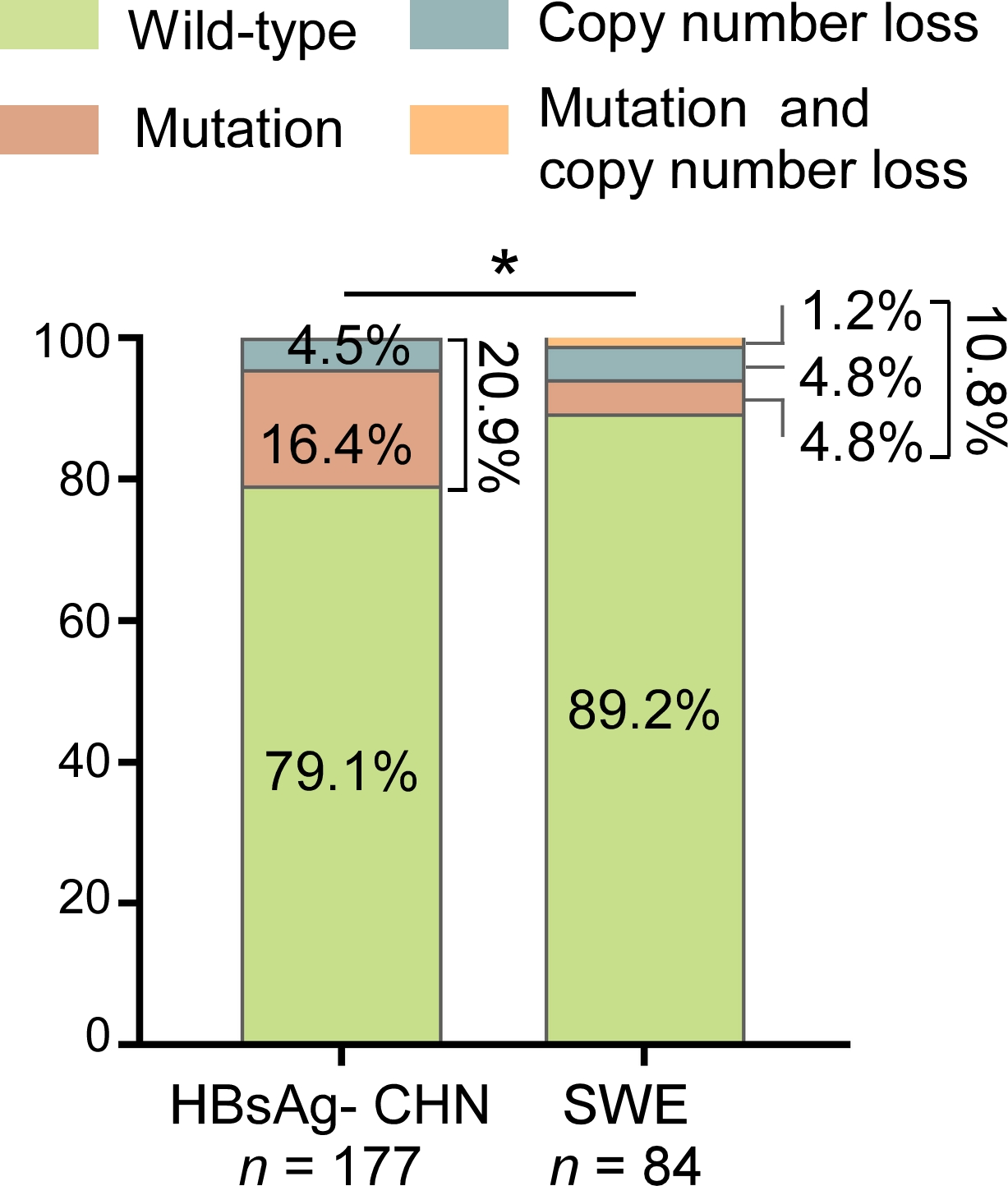
**

**Supplementary Figure 6.** Comparison of the frequency of *CD70* mutations and copy number variations in HBsAg-CHN and SWE DLBCL tumor samples. χ^2^ test was used for the comparison, **P* < 0.05.

**
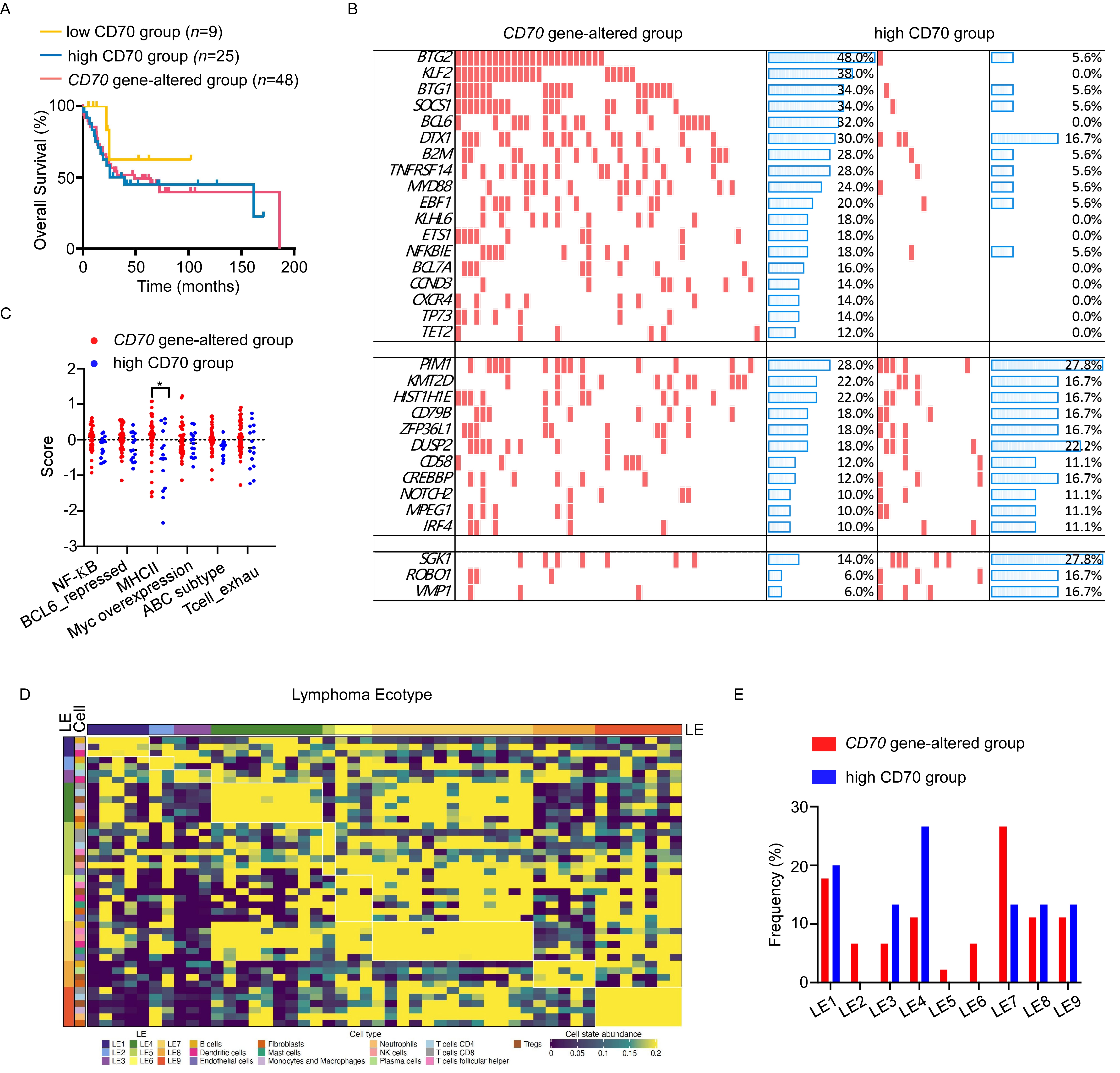
**

**Supplementary Figure 7.** The comparison of survival, mutation pattern and gene expression profiling in the indicated DLBCL groups.

A. Kaplan‒Meier survival curve of overall survival for Chinese DLBCL patients with available genetic, protein staining and clinical data. The patients were divided into three groups: 1) *CD70* gene-altered group (mutations and copy number loss); 2) *CD7*0 WT and low CD70 protein expression (low CD70 group); 3) *CD70* WT and with high CD70 protein expression (high CD70 group). The *P* value was determined by the log-rank test. *P* = 0.54.

B. The mutation frequency of genes was compared in *CD70* gene-altered group and high CD70 group.

C. The enriched pathways were compared in *CD70* gene-altered group and high CD70 group . * *P* < 0.05.

D-E. Lymphoma ecotypes in the Chinese cohort were characterized and compared in *CD70* gene-altered group and high CD70 group.

**
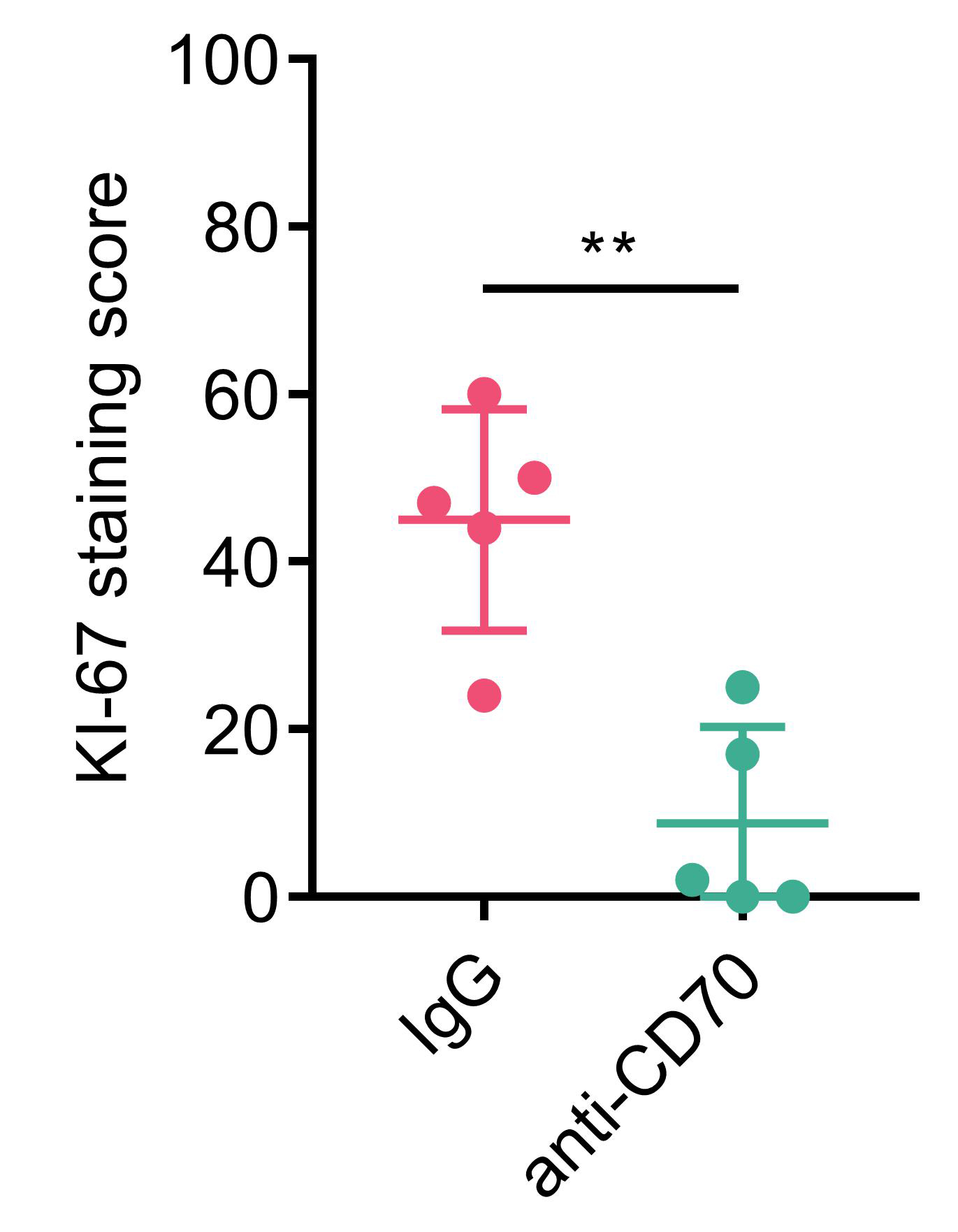
**

**Supplementary Figure 8.** The KI67 score in A20 cell-bearing BALB/c mice treated with isotype-matched IgG or anti-CD70. Student’s t test, ***P* < 0.01.

**Reference**

1. Reddy A, Zhang J, Davis NS, et al. Genetic and Functional Drivers of Diffuse Large B Cell Lymphoma. *Cell*. Oct 5 2017;171(2):481-494 e15. doi:10.1016/j.cell.2017.09.027

2. Chapuy B, Stewart C, Dunford AJ, et al. Molecular subtypes of diffuse large B cell lymphoma are associated with distinct pathogenic mechanisms and outcomes. *Nat Med*. May 2018;24(5):679-690. doi:10.1038/s41591-018-0016-8

3. Lohr JG, Stojanov P, Lawrence MS, et al. Discovery and prioritization of somatic mutations in diffuse large B-cell lymphoma (DLBCL) by whole-exome sequencing. *Proc Natl Acad Sci U S A*. Mar 6 2012;109(10):3879-84. doi:10.1073/pnas.1121343109

4. Morin RD, Mungall K, Pleasance E, et al. Mutational and structural analysis of diffuse large B-cell lymphoma using whole-genome sequencing. *Blood*. Aug 15 2013;122(7):1256-65. doi:10.1182/blood-2013-02-483727

5. Lacy SE, Barrans SL, Beer PA, et al. Targeted sequencing in DLBCL, molecular subtypes, and outcomes: a Haematological Malignancy Research Network report. *Blood*. May 14 2020;135(20):1759-1771. doi:10.1182/blood.2019003535

**Supplementary Tables**

Table S1. The experiments performed and clinical data of the Chinese DLBCL cohort.

Table S2. The experiments performed and clinical data of the Swedish DLBCL cohort.

Table S3. CD70 mutations identified in the Chinese DLBCL cohort.

Table S4. CD70 mutations identified in the Swedish DLBCL cohort.

Table S5. CD70 genetic alterations in all cancer types: Table S5a. CD70 genetic alterations in all cancer types from the COSMIC database; Table S5b. CD70 genetic alterations in hematopoietic malignancies from the COSMIC database.

Table S6. Baseline characteristics of DLBCL patients with different CD70 genetic statuses: Table S6a. Baseline characteristics of Chinese DLBCL patients with different CD70 genetic statuses; Table S6b. Univariate and multivariate analysis of OS for Chinese DLBCL patients; Table S6c. Baseline characteristics of Swedish DLBCL patients with different CD70 genetic statuses.

Table S7. Baseline characteristics of Swedish DLBCL patients with different CD70 protein expression levels.

Table S8. Sample information and numbers of different subclusters of cells for each sample by single-cell RNA sequencing.
