# Supplementary material for: The dual role of CD70 in B‐cell lymphomagenesis
Source: Clin Transl Med. 2022 Dec 5;12(12):e1118. doi: 10.1002/ctm2.1118 (PMC9722974; doi:10.1002/ctm2.1118)
Supplement: Supplementary file 2 — Supporting Information [file CTM2-12-e1118-s004.docx]

S1. The experiments performed and clinical data of the Chinese DLBCL cohort.

| **Sample ID** | **Gender** | **Age** | **Primay/Relapse**  **biopsy** | **Molecular subtype** | **Ann Arbor Stage** | **IPI score** | **HBSAg** | **EBER** | **CD70 WT/non-WT** | **CD70 Copy number** | **CD70 mutation** | **WGS** | **WES** | **Lymphochip** | **CD70 Sanger** | **CD70 CNV analysis** | **RNAseq** | **CD70 IHC Staining** |
| --- | --- | --- | --- | --- | --- | --- | --- | --- | --- | --- | --- | --- | --- | --- | --- | --- | --- | --- |
| Case-384 | Female | 17 | Primary | non-GCB | III | 2 | - | NA | WT | 2 copies | No | No | No | Yes | No | Yes | Yes | No |
| Case-37 | Female | 21 | Relapse | non-GCB | II | 0 | + | No | WT | 2 copies | No | Yes | Yes | No | Yes | Yes | Yes | Yes |
| Case-299 | Female | 22 | Relapse | non-GCB | II | 1 | - | NA | WT | 2 copies | No | No | No | Yes | Yes | Yes | Yes | No |
| Case-416 | Female | 23 | Primary | GCB | IV | 2 | - | NA | WT | 2 copies | No | No | No | Yes | No | Yes | No | No |
| Case-25 | Female | 26 | Primary | GCB | IV | 3 | + | No | WT | 2 copies | No | Yes | No | No | No | Yes | Yes | No |
| Case-291 | Female | 27 | Primary | GCB | II | 2 | - | No | WT | 2 copies | No | No | No | Yes | Yes | Yes | Yes | No |
| Case-468 | Female | 27 | Relapse | non-GCB | II | 0 | - | No | WT | 2 copies | No | No | No | Yes | Yes | Yes | No | No |
| Case-23 | Female | 28 | Relapse | non-GCB | II | 1 | - | No | NA | NA | No | Yes | No | No | No | No | Yes | Yes |
| Case-93 | Female | 30 | Primary | non-GCB | IV | 4 | + | No | NA | NA | No | No | No | Yes | Yes | No | Yes | No |
| Case-498 | Female | 30 | Primary | non-GCB | III | 2 | NA | No | WT | 2 copies | No | No | No | No | Yes | Yes | Yes | No |
| Case-39 | Female | 31 | Primary | non-GCB | IV | 4 | + | No | NA | NA | Mutation | Yes | No | No | Yes | No | Yes | Yes |
| Case-461 | Female | 31 | Primary | non-GCB | IV | 3 | - | No | WT | 2 copies | No | No | No | Yes | Yes | Yes | Yes | No |
| Case-460 | Female | 32 | Primary | GCB | IV | 3 | + | Yes | WT | 2 copies | No | No | No | Yes | Yes | Yes | Yes | Yes |
| Case-332 | Female | 34 | Primary | GCB | I | 0 | + | No | WT | 2 copies | No | No | No | Yes | No | Yes | Yes | No |
| Case-372 | Female | 34 | Primary | GCB | II | 0 | - | NA | NA | NA | No | No | No | Yes | No | No | No | No |
| Case-396 | Female | 37 | Primary | non-GCB | II | 1 | + | NA | WT | 2 copies | No | No | No | Yes | No | Yes | No | No |
| Case-103 | Female | 38 | Primary | GCB | II | 1 | - | Yes | WT | 2 copies | No | No | No | No | Yes | Yes | Yes | Yes |
| Case-112 | Female | 38 | Primary | non-GCB | II | 0 | + | No | WT | 2 copies | No | No | Yes | No | No | Yes | Yes | No |
| Case-5 | Female | 39 | Primary | GCB | IV | 3 | + | No | WT | 2 copies | No | Yes | Yes | No | Yes | Yes | Yes | Yes |
| Case-284 | Female | 39 | Primary | non-GCB | II | 1 | - | Yes | NA | NA | No | No | No | Yes | Yes | No | No | No |
| Case-287 | Female | 41 | Relapse | non-GCB | III | 1 | - | No | NA | NA | Mutation | No | No | Yes | Yes | No | Yes | Yes |
| Case-98 | Female | 43 | Primary | GCB | II | 1 | - | No | WT | 2 copies | No | No | Yes | No | Yes | Yes | Yes | Yes |
| Case-495 | Female | 43 | Primary | GCB | II | 0 | NA | NA | non-WT | 2 copies | Mutation | No | No | No | Yes | Yes | No | No |
| Case-26 | Female | 44 | Relapse | non-GCB | II | 2 | - | No | NA | NA | Mutation | Yes | No | No | Yes | No | Yes | No |
| Case-128 | Female | 45 | Relapse | GCB | III | 2 | - | NA | WT | 2 copies | No | No | No | Yes | Yes | Yes | Yes | No |
| Case-50 | Female | 46 | Primary | non-GCB | II | 1 | - | No | NA | NA | No | Yes | No | No | Yes | No | Yes | Yes |
| Case-424 | Female | 46 | Primary | non-GCB | II | 0 | - | NA | NA | NA | No | No | No | Yes | No | No | No | No |
| Case-486 | Female | 48 | Primary | non-GCB | IV | 4 | + | NA | WT | 2 copies | No | No | No | No | Yes | Yes | Yes | No |
| Case-286 | Female | 49 | Relapse | non-GCB | I | 1 | - | No | NA | NA | No | No | No | Yes | Yes | No | No | Yes |
| Case-341 | Female | 50 | Primary | GCB | II | 2 | + | No | non-WT | 2 copies | Mutation | No | No | Yes | Yes | Yes | Yes | Yes |
| Case-43 | Female | 51 | Primary | non-GCB | III | 4 | - | No | WT | 2 copies | No | Yes | No | No | Yes | Yes | Yes | Yes |
| Case-99 | Female | 52 | Primary | non-GCB | II | 0 | + | No | WT | 2 copies | No | No | No | No | Yes | Yes | Yes | No |
| Case-106 | Female | 52 | Primary | non-GCB | II | 0 | - | No | WT | 2 copies | No | No | Yes | No | No | Yes | Yes | No |
| Case-282 | Female | 52 | Primary | non-GCB | III | 2 | + | No | NA | NA | Mutation | No | No | Yes | Yes | No | Yes | No |
| Case-408 | Female | 52 | Primary | GCB | II | 1 | - | NA | WT | 2 copies | No | No | No | Yes | No | Yes | Yes | No |
| Case-490 | Female | 52 | Primary | non-GCB | III | 1 | - | No | NA | NA | No | No | No | No | Yes | No | No | No |
| Case-442 | Female | 53 | Primary | GCB | IV | 2 | - | NA | WT | 2 copies | No | No | No | Yes | No | Yes | No | No |
| Case-317 | Female | 54 | Primary | GCB | III | 1 | - | No | NA | NA | Mutation | No | No | Yes | Yes | No | Yes | No |
| Case-391 | Female | 54 | Primary | GCB | IV | 3 | - | NA | WT | 2 copies | No | No | No | Yes | No | Yes | No | No |
| Case-426 | Female | 54 | Primary | non-GCB | III | 3 | - | NA | WT | 2 copies | No | No | No | Yes | No | Yes | Yes | No |
| Case-324 | Female | 55 | Relapse | GCB | IV | 3 | - | No | WT | 2 copies | No | No | No | Yes | No | Yes | Yes | No |
| Case-367 | Female | 55 | Primary | GCB | IV | 1 | - | No | WT | 2 copies | No | No | Yes | No | No | Yes | No | No |
| Case-388 | Female | 55 | Primary | non-GCB | IV | 2 | - | NA | WT | 2 copies | No | No | No | Yes | No | Yes | Yes | No |
| Case-494 | Female | 55 | Relapse | GCB | II | 0 | NA | No | WT | 2 copies | No | No | No | No | Yes | Yes | Yes | No |
| Case-3 | Female | 56 | Relapse | non-GCB | I | 0 | - | No | WT | 2 copies | No | Yes | No | No | No | Yes | Yes | No |
| Case-432 | Female | 56 | Primary | GCB | II | 1 | - | NA | NA | NA | No | No | No | Yes | No | No | No | No |
| Case-439 | Female | 56 | Primary | non-GCB | II | 0 | - | NA | WT | 2 copies | No | No | No | Yes | No | Yes | No | No |
| Case-471 | Female | 56 | Primary | non-GCB | III | 3 | - | No | WT | 2 copies | No | No | No | Yes | Yes | Yes | No | Yes |
| Case-493 | Female | 56 | Relapse | NA | II | 1 | NA | No | WT | 2 copies | No | No | No | No | Yes | Yes | Yes | No |
| Case-273 | Female | 57 | Primary | GCB | II | 2 | - | NA | non-WT | 2 copies | Mutation | No | No | Yes | Yes | Yes | Yes | No |
| Case-386 | Female | 57 | Primary | non-GCB | III | 1 | - | NA | WT | 2 copies | No | No | No | Yes | No | Yes | No | No |
| Case-438 | Female | 57 | Primary | non-GCB | II | 1 | - | NA | WT | 2 copies | No | No | No | Yes | No | Yes | No | No |
| Case-455 | Female | 57 | Primary | non-GCB | IV | 2 | - | NA | WT | 2 copies | No | No | No | Yes | No | Yes | Yes | No |
| Case-113 | Female | 58 | Primary | non-GCB | III | 1 | - | Yes | WT | 2 copies | No | No | Yes | No | No | Yes | Yes | No |
| Case-335 | Female | 58 | Primary | non-GCB | IV | 3 | - | No | NA | NA | No | No | No | Yes | Yes | No | Yes | No |

| Case-369 | Female | 58 | Primary | non-GCB | III | 2 | - | NA | WT | 2 copies | No | No | No | Yes | No | Yes | No | No |
| --- | --- | --- | --- | --- | --- | --- | --- | --- | --- | --- | --- | --- | --- | --- | --- | --- | --- | --- |
| Case-393 | Female | 58 | Primary | non-GCB | II | 0 | - | NA | WT | 2 copies | No | No | No | Yes | No | Yes | Yes | No |
| Case-429 | Female | 58 | Primary | GCB | IV | 2 | - | NA | WT | 2 copies | No | No | No | Yes | No | Yes | No | No |
| Case-57 | Female | 59 | Primary | GCB | II | 1 | - | No | WT | 2 copies | No | Yes | No | No | No | Yes | Yes | No |
| Case-373 | Female | 59 | Primary | non-GCB | I | 0 | - | NA | WT | 2 copies | No | No | No | Yes | No | Yes | No | No |
| Case-476 | Female | 59 | Primary | GCB | III | 2 | - | NA | NA | NA | No | No | No | Yes | Yes | No | Yes | No |
| Case-12 | Female | 60 | Primary | non-GCB | II | 1 | + | No | non-WT | 2 copies | Mutation | Yes | No | No | No | Yes | Yes | Yes |
| Case-133 | Female | 60 | Relapse | non-GCB | III | 2 | - | No | WT | 2 copies | No | No | Yes | No | Yes | Yes | Yes | No |
| Case-397 | Female | 60 | Primary | GCB | I | 0 | - | NA | non-WT | 2 copies | Mutation | No | No | Yes | No | Yes | Yes | No |
| Case-368 | Female | 61 | Primary | GCB | I | 1 | - | NA | WT | 2 copies | No | No | No | Yes | No | Yes | No | No |
| Case-401 | Female | 61 | Primary | non-GCB | II | 2 | - | NA | WT | 2 copies | No | No | No | Yes | No | Yes | No | No |
| Case-405 | Female | 61 | Primary | non-GCB | II | 1 | - | NA | WT | 2 copies | No | No | No | Yes | No | Yes | Yes | No |
| Case-502 | Female | 61 | Primary | GCB | III | 1 | + | No | non-WT | 2 copies | Mutation | No | No | No | Yes | Yes | Yes | No |
| Case-278 | Female | 62 | Relapse | GCB | II | 1 | - | No | NA | NA | No | No | No | Yes | Yes | No | Yes | No |
| Case-318 | Female | 62 | Primary | non-GCB | III | 4 | - | No | WT | 2 copies | No | No | No | Yes | No | Yes | Yes | No |
| Case-333 | Female | 62 | Primary | non-GCB | NA | NA | - | No | WT | 2 copies | No | No | No | Yes | No | Yes | Yes | No |
| Case-411 | Female | 62 | Primary | non-GCB | IV | 3 | + | NA | non-WT | 2 copies | Mutation | No | No | Yes | No | Yes | No | No |
| Case-423 | Female | 62 | Primary | GCB | I | 2 | - | NA | NA | NA | No | No | No | Yes | No | No | Yes | No |
| Case-449 | Female | 62 | Primary | non-GCB | II | 2 | - | NA | WT | 2 copies | No | No | No | Yes | No | Yes | Yes | No |
| Case-58 | Female | 63 | Primary | non-GCB | III | 2 | - | Yes | WT | 2 copies | No | Yes | No | No | No | Yes | Yes | Yes |
| Case-311 | Female | 63 | Primary | non-GCB | NA | NA | + | No | WT | 2 copies | No | No | No | Yes | No | Yes | Yes | No |
| Case-323 | Female | 63 | Primary | non-GCB | NA | NA | - | No | non-WT | 2 copies | Mutation | No | No | Yes | No | Yes | Yes | No |
| Case-506 | Female | 63 | Relapse | non-GCB | II | 0 | NA | No | WT | 2 copies | No | No | No | No | Yes | Yes | No | No |
| Case-385 | Female | 64 | Primary | GCB | II | 1 | - | NA | NA | NA | No | No | No | Yes | No | No | Yes | No |
| Case-418 | Female | 64 | Primary | GCB | IV | 1 | - | NA | WT | 2 copies | No | No | No | Yes | No | Yes | Yes | No |
| Case-31 | Female | 65 | Primary | non-GCB | II | 1 | - | No | WT | 2 copies | No | Yes | No | No | No | Yes | Yes | No |
| Case-97 | Female | 65 | Primary | non-GCB | III | 2 | - | No | non-WT | 2 copies | Mutation | No | Yes | No | Yes | Yes | Yes | Yes |
| Case-268 | Female | 65 | Primary | non-GCB | III | 2 | - | No | NA | NA | Mutation | No | No | Yes | Yes | No | No | Yes |
| Case-319 | Female | 65 | Primary | non-GCB | NA | NA | - | No | WT | 2 copies | No | No | No | Yes | No | Yes | Yes | No |
| Case-321 | Female | 65 | Primary | non-GCB | II | 2 | - | No | WT | 2 copies | No | No | No | Yes | No | Yes | Yes | No |
| Case-8 | Female | 67 | Primary | non-GCB | I | 2 | - | No | non-WT | 2 copies | Mutation | Yes | Yes | No | Yes | Yes | Yes | Yes |
| Case-36 | Female | 67 | Primary | non-GCB | IV | 5 | - | NA | non-WT | 1 copy | No | Yes | Yes | No | Yes | Yes | Yes | No |
| Case-46 | Female | 67 | Relapse | non-GCB | III | 2 | - | No | WT | 2 copies | No | Yes | Yes | No | Yes | Yes | Yes | No |
| Case-120 | Female | 67 | Primary | GCB | III | 3 | + | No | WT | 2 copies | No | No | Yes | No | No | Yes | Yes | No |
| Case-452 | Female | 67 | Primary | GCB | II | 1 | - | NA | WT | 2 copies | No | No | No | Yes | No | Yes | Yes | No |
| Case-41 | Female | 68 | Primary | non-GCB | IV | 3 | - | No | WT | 2 copies | No | Yes | No | No | No | Yes | Yes | Yes |
| Case-42 | Female | 68 | Primary | non-GCB | IV | 5 | - | No | WT | 2 copies | No | Yes | Yes | No | Yes | Yes | Yes | Yes |
| Case-381 | Female | 68 | Primary | GCB | IV | 3 | + | NA | WT | 2 copies | No | No | No | Yes | No | Yes | Yes | No |
| Case-414 | Female | 68 | Primary | non-GCB | II | 1 | - | NA | WT | 2 copies | No | No | No | Yes | No | Yes | Yes | No |
| Case-503 | Female | 68 | Relapse | GCB | III | 3 | NA | No | WT | 2 copies | No | No | No | No | Yes | Yes | No | No |
| Case-1 | Female | 69 | Primary | non-GCB | II | 1 | - | Yes | non-WT | 2 copies | Mutation | Yes | Yes | No | Yes | Yes | Yes | Yes |
| Case-44 | Female | 69 | Primary | GCB | II | 4 | - | No | NA | NA | No | Yes | No | No | No | No | Yes | Yes |
| Case-304 | Female | 69 | Relapse | GCB | II | 3 | - | No | NA | NA | No | No | No | Yes | No | No | Yes | No |
| Case-40 | Female | 70 | Primary | non-GCB | IV | 5 | - | No | WT | 2 copies | No | Yes | No | No | No | Yes | Yes | Yes |
| Case-96 | Female | 70 | Primary | GCB | II | 2 | - | No | WT | 2 copies | No | No | Yes | No | Yes | Yes | Yes | Yes |
| Case-295 | Female | 70 | Primary | non-GCB | IV | 5 | - | No | non-WT | 2 copies | Mutation | No | No | Yes | Yes | Yes | Yes | No |
| Case-303 | Female | 70 | Primary | non-GCB | NA | NA | - | Yes | WT | 2 copies | No | No | No | Yes | No | Yes | No | No |
| Case-101 | Female | 71 | Primary | non-GCB | III | 3 | - | Yes | WT | 2 copies | No | No | Yes | No | Yes | Yes | Yes | Yes |
| Case-4 | Female | 72 | Primary | non-GCB | II | 2 | - | No | NA | NA | Mutation | Yes | No | No | Yes | No | Yes | Yes |
| Case-18 | Female | 73 | Primary | GCB | IV | 4 | - | no | non-WT | 2 copies | Mutation | Yes | Yes | No | Yes | Yes | Yes | Yes |
| Case-427 | Female | 73 | Primary | non-GCB | I | 1 | - | NA | WT | 2 copies | No | No | No | Yes | No | Yes | No | No |
| Case-456 | Female | 73 | Primary | non-GCB | IV | 4 | - | NA | WT | 2 copies | No | No | No | Yes | No | Yes | No | No |
| Case-7 | Female | 75 | Primary | non-GCB | IV | 5 | - | No | non-WT | no copy was detected | No | Yes | No | No | No | Yes | Yes | Yes |
| Case-114 | Female | 77 | Primary | non-GCB | III | 3 | - | No | WT | 2 copies | No | No | Yes | No | No | Yes | Yes | No |
| Case-298 | Female | 77 | Relapse | non-GCB | I | 1 | - | NA | non-WT | 2 copies | Mutation | No | No | Yes | Yes | Yes | Yes | No |
| Case-434 | Female | 77 | Primary | non-GCB | II | 2 | - | NA | WT | 2 copies | No | No | No | Yes | No | Yes | Yes | No |
| Case-374 | Female | 81 | Primary | non-GCB | NA | NA | - | NA | WT | 2 copies | No | No | No | Yes | No | Yes | No | No |
| Case-446 | Female | 85 | Primary | GCB | II | 2 | - | NA | WT | 2 copies | No | No | No | Yes | No | Yes | No | No |
| Case-488 | Female | 86 | Primary | NA | IV | 4 | NA | NA | NA | NA | No | No | No | No | Yes | No | Yes | No |

| Case-464 | Male | 12 | Relapse | GCB | III | 2 | - | No | WT | 2 copies | No | No | No | Yes | Yes | Yes | Yes | No |
| --- | --- | --- | --- | --- | --- | --- | --- | --- | --- | --- | --- | --- | --- | --- | --- | --- | --- | --- |
| Case-487 | Male | 19 | Primary | GCB | II | 0 | - | NA | NA | NA | No | No | No | No | Yes | No | Yes | No |
| Case-289 | Male | 23 | Primary | GCB | III | 2 | - | NA | NA | NA | No | No | No | Yes | Yes | No | Yes | No |
| Case-301 | Male | 23 | Relapse | non-GCB | III | 2 | - | No | NA | NA | No | No | No | Yes | Yes | No | Yes | No |
| Case-387 | Male | 23 | Primary | non-GCB | IV | 4 | - | NA | WT | 2 copies | No | No | No | Yes | No | Yes | No | No |
| Case-302 | Male | 24 | Primary | non-GCB | III | 1 | + | No | WT | 2 copies | No | No | No | Yes | No | Yes | Yes | No |
| Case-492 | Male | 24 | Primary | GCB | I | 0 | - | No | WT | 2 copies | No | No | No | No | Yes | Yes | No | No |
| Case-134 | Male | 27 | Primary | GCB | III | 2 | + | No | non-WT | 2 copies | Mutation | No | Yes | No | Yes | Yes | Yes | No |
| Case-28 | Male | 28 | Primary | non-GCB | III | 1 | + | No | WT | 2 copies | No | Yes | No | No | No | Yes | Yes | Yes |
| Case-457 | Male | 28 | Primary | GCB | II | 1 | + | NA | non-WT | 2 copies | Mutation | No | No | Yes | Yes | Yes | Yes | No |
| Case-48 | Male | 29 | Primary | non-GCB | II | 1 | - | No | WT | 2 copies | No | Yes | No | No | No | Yes | Yes | Yes |
| Case-290 | Male | 29 | Relapse | non-GCB | II | 2 | - | No | WT | 2 copies | No | No | No | Yes | Yes | Yes | Yes | No |
| Case-309 | Male | 29 | Relapse | non-GCB | III | 3 | + | No | WT | 2 copies | No | No | No | Yes | No | Yes | Yes | No |
| Case-336 | Male | 29 | Primary | non-GCB | III | 3 | + | No | non-WT | 2 copies | Mutation | No | No | Yes | Yes | Yes | Yes | No |
| Case-382 | Male | 29 | Primary | GCB | IV | 3 | + | NA | NA | NA | No | No | No | Yes | No | No | Yes | No |
| Case-406 | Male | 29 | Primary | GCB | II | 0 | - | NA | non-WT | 1 copy | No | No | No | Yes | No | Yes | No | No |
| Case-346 | Male | 30 | Primary | non-GCB | II | 1 | - | No | WT | 2 copies | No | No | Yes | No | Yes | Yes | Yes | Yes |
| Case-20 | Male | 31 | Relapse | non-GCB | I | 0 | + | No | NA | NA | No | Yes | No | No | No | No | Yes | Yes |
| Case-394 | Male | 31 | Primary | non-GCB | II | 0 | - | NA | WT | 2 copies | No | No | No | Yes | No | Yes | No | No |
| Case-475 | Male | 31 | Relapse | GCB | IV | 4 | + | Yes | WT | 2 copies | No | No | No | Yes | Yes | Yes | No | No |
| Case-281 | Male | 34 | Relapse | GCB | I | 0 | - | No | WT | 2 copies | No | No | No | Yes | Yes | Yes | Yes | No |
| Case-313 | Male | 35 | Primary | GCB | II | 1 | - | No | WT | 2 copies | No | No | No | Yes | No | Yes | Yes | No |
| Case-132 | Male | 36 | Primary | non-GCB | III | 2 | - | NA | non-WT | 2 copies | Mutation | No | No | Yes | Yes | Yes | Yes | No |
| Case-55 | Male | 37 | Primary | non-GCB | II | 0 | - | No | WT | 2 copies | No | Yes | Yes | No | Yes | Yes | No | No |
| Case-100 | Male | 37 | Primary | non-GCB | III | 1 | - | No | non-WT | 2 copies | Mutation | No | Yes | No | Yes | Yes | Yes | Yes |
| Case-316 | Male | 37 | Relapse | non-GCB | IV | 4 | + | No | NA | NA | No | No | No | Yes | No | No | Yes | No |
| Case-327 | Male | 37 | Primary | GCB | NA | NA | - | No | WT | 2 copies | No | No | No | Yes | No | Yes | No | No |
| Case-425 | Male | 37 | Primary | GCB | I | 0 | - | NA | NA | NA | No | No | No | Yes | No | No | No | No |
| Case-17 | Male | 38 | Primary | non-GCB | II | 0 | + | No | WT | 2 copies | No | Yes | Yes | No | Yes | Yes | Yes | Yes |
| Case-277 | Male | 39 | Primary | non-GCB | II | 0 | + | NA | NA | NA | No | No | No | Yes | Yes | No | Yes | No |
| Case-448 | Male | 39 | Primary | GCB | I | 1 | - | NA | NA | NA | No | No | No | Yes | No | No | Yes | No |
| Case-478 | Male | 39 | Primary | GCB | III | 1 | + | No | non-WT | 2 copies | Mutation | No | No | Yes | Yes | Yes | No | No |
| Case-56 | Male | 40 | Primary | GCB | II | 0 | - | No | WT | 2 copies | No | Yes | No | No | No | Yes | Yes | No |
| Case-45 | Male | 41 | Primary | non-GCB | IV | 4 | + | No | NA | NA | No | Yes | No | No | Yes | No | No | Yes |
| Case-83 | Male | 41 | Primary | non-GCB | III | 4 | + | No | WT | 2 copies | No | No | No | Yes | Yes | Yes | Yes | Yes |
| Case-131 | Male | 41 | Primary | GCB | IV | 4 | + | No | non-WT | no copy was detected | No | No | Yes | No | Yes | Yes | Yes | No |
| Case-296 | Male | 41 | Relapse | non-GCB | IV | 3 | + | NA | non-WT | 2 copies | Mutation | No | No | Yes | Yes | Yes | Yes | No |
| Case-297 | Male | 41 | Relapse | non-GCB | I | 0 | + | NA | non-WT | 2 copies | Mutation | No | No | Yes | Yes | Yes | Yes | No |
| Case-306 | Male | 41 | Primary | non-GCB | III | 2 | + | NA | WT | 2 copies | No | No | No | Yes | No | Yes | Yes | No |
| Case-285 | Male | 42 | Relapse | non-GCB | IV | 2 | - | No | NA | NA | No | No | No | Yes | Yes | No | No | Yes |
| Case-479 | Male | 42 | Primary | non-GCB | III | 1 | + | No | WT | 2 copies | No | No | No | Yes | Yes | Yes | Yes | No |
| Case-11 | Male | 44 | Primary | GCB | III | 1 | - | No | NA | NA | Mutation | Yes | No | No | No | No | No | No |
| Case-84 | Male | 44 | Primary | non-GCB | I | 0 | + | NA | NA | NA | No | No | No | Yes | Yes | No | Yes | No |
| Case-293 | Male | 44 | Relapse | GCB | II | 2 | - | No | WT | 2 copies | No | No | No | Yes | Yes | Yes | Yes | No |
| Case-315 | Male | 44 | Primary | GCB | III | 1 | + | NA | WT | 2 copies | No | No | No | Yes | No | Yes | Yes | No |
| Case-325 | Male | 44 | Relapse | non-GCB | III | 1 | + | No | non-WT | 1 copy | No | No | No | Yes | No | Yes | Yes | No |
| Case-60 | Male | 45 | Relapse | GCB | II | 0 | - | No | WT | 2 copies | No | Yes | No | No | No | Yes | Yes | Yes |
| Case-92 | Male | 45 | Primary | non-GCB | III | 2 | + | No | non-WT | 2 copies | Mutation | No | Yes | No | Yes | Yes | Yes | Yes |
| Case-337 | Male | 45 | Primary | non-GCB | II | 1 | - | No | NA | NA | Mutation | No | No | Yes | Yes | No | No | No |
| Case-22 | Male | 46 | Primary | GCB | II | 1 | - | No | non-WT | 2 copies | Mutation | Yes | No | No | No | Yes | Yes | Yes |
| Case-54 | Male | 46 | Primary | GCB | III | 2 | - | No | WT | 2 copies | No | Yes | No | No | No | Yes | Yes | Yes |
| Case-499 | Male | 46 | Primary | non-GCB | III | 4 | NA | NA | WT | 2 copies | No | No | No | No | Yes | Yes | Yes | No |
| Case-117 | Male | 47 | Relapse | GCB | II | 0 | - | No | WT | 2 copies | No | No | Yes | No | No | Yes | Yes | No |
| Case-16 | Male | 48 | Primary | non-GCB | IV | 4 | + | No | NA | NA | Mutation | Yes | No | No | Yes | No | Yes | Yes |
| Case-127 | Male | 48 | Relapse | non-GCB | IV | 3 | - | No | NA | NA | No | No | Yes | No | Yes | No | Yes | No |
| Case-280 | Male | 48 | Primary | GCB | IV | 2 | - | No | NA | NA | No | No | No | Yes | Yes | No | Yes | No |
| Case-383 | Male | 48 | Primary | GCB | II | 0 | - | NA | non-WT | 2 copies | Mutation | No | No | Yes | No | Yes | Yes | No |
| Case-443 | Male | 48 | Primary | non-GCB | II | 0 | - | NA | WT | 2 copies | No | No | No | Yes | No | Yes | Yes | No |
| Case-51 | Male | 49 | Primary | non-GCB | IV | 3 | - | No | WT | 2 copies | No | Yes | No | No | No | Yes | Yes | No |

| Case-421 | Male | 49 | Primary | non-GCB | III | 2 | - | NA | WT | 2 copies | No | No | No | Yes | No | Yes | No | No |
| --- | --- | --- | --- | --- | --- | --- | --- | --- | --- | --- | --- | --- | --- | --- | --- | --- | --- | --- |
| Case-300 | Male | 50 | Primary | non-GCB | III | 2 | - | NA | WT | 2 copies | No | No | No | Yes | Yes | Yes | Yes | No |
| Case-328 | Male | 50 | Primary | non-GCB | II | 0 | - | No | NA | NA | No | No | No | Yes | Yes | No | Yes | No |
| Case-376 | Male | 50 | Primary | non-GCB | NA | NA | - | NA | NA | NA | No | No | No | Yes | No | No | Yes | No |
| Case-500 | Male | 50 | Relapse | GCB | III | 1 | - | No | non-WT | 2 copies | Mutation | No | No | No | Yes | Yes | No | Yes |
| Case-102 | Male | 51 | Primary | non-GCB | III | 4 | - | NA | WT | 2 copies | No | No | No | Yes | Yes | Yes | Yes | No |
| Case-88 | Male | 52 | Primary | non-GCB | IV | 3 | + | No | non-WT | 2 copies | Mutation | No | No | Yes | Yes | Yes | Yes | Yes |
| Case-104 | Male | 52 | Primary | non-GCB | I | 1 | - | No | WT | 2 copies | No | No | Yes | No | No | Yes | Yes | No |
| Case-326 | Male | 52 | Primary | GCB | II | 0 | - | No | WT | 2 copies | No | No | No | Yes | No | Yes | Yes | No |
| Case-437 | Male | 52 | Primary | GCB | II | 1 | + | NA | WT | 2 copies | No | No | No | Yes | No | Yes | No | No |
| Case-30 | Male | 53 | Primary | non-GCB | I | 0 | - | No | non-WT | 2 copies | Mutation | Yes | No | No | No | Yes | Yes | No |
| Case-38 | Male | 53 | Primary | GCB | II | 0 | - | No | non-WT | no copy was detected | No | Yes | No | No | No | Yes | Yes | No |
| Case-53 | Male | 54 | Primary | non-GCB | II | 0 | - | No | WT | 2 copies | No | Yes | No | No | No | Yes | Yes | Yes |
| Case-484 | Male | 54 | Primary | non-GCB | IV | 4 | + | No | non-WT | 2 copies | Mutation | No | No | No | Yes | Yes | No | No |
| Case-497 | Male | 54 | Primary | non-GCB | III | 2 | NA | No | non-WT | no copy was detected | No | No | No | No | Yes | Yes | Yes | No |
| Case-32 | Male | 55 | Relapse | GCB | III | 3 | + | No | non-WT | 2 copies | Mutation | Yes | No | No | No | Yes | Yes | Yes |
| Case-49 | Male | 55 | Primary | non-GCB | IV | 3 | + | No | WT | 2 copies | No | Yes | No | No | No | Yes | Yes | Yes |
| Case-59 | Male | 55 | Primary | GCB | III | 3 | - | No | NA | NA | No | Yes | No | No | Yes | No | Yes | No |
| Case-125 | Male | 55 | Relapse | non-GCB | III | 1 | - | No | WT | 2 copies | No | No | Yes | No | Yes | Yes | Yes | Yes |
| Case-276 | Male | 55 | Primary | non-GCB | II | 0 | + | NA | NA | NA | No | No | No | Yes | Yes | No | Yes | No |
| Case-320 | Male | 55 | Primary | non-GCB | NA | NA | + | Yes | WT | 2 copies | No | No | No | Yes | No | Yes | Yes | No |
| Case-413 | Male | 55 | Primary | GCB | II | 1 | - | NA | non-WT | 2 copies | Mutation | No | No | Yes | No | Yes | No | No |
| Case-436 | Male | 55 | Primary | non-GCB | II | 0 | - | NA | WT | 2 copies | No | No | No | Yes | No | Yes | Yes | No |
| Case-441 | Male | 55 | Primary | GCB | II | 0 | - | NA | WT | 2 copies | No | No | No | Yes | No | Yes | Yes | No |
| Case-390 | Male | 56 | Primary | non-GCB | II | 0 | - | NA | WT | 2 copies | No | No | No | Yes | No | Yes | No | No |
| Case-444 | Male | 56 | Primary | non-GCB | I | 0 | - | NA | WT | 2 copies | No | No | No | Yes | No | Yes | Yes | No |
| Case-269 | Male | 57 | Primary | GCB | II | 1 | - | No | non-WT | 2 copies | Mutation | No | No | Yes | Yes | Yes | Yes | No |
| Case-272 | Male | 57 | Primary | non-GCB | III | 2 | + | No | NA | NA | No | No | No | Yes | Yes | No | Yes | No |
| Case-288 | Male | 57 | Primary | non-GCB | II | 1 | - | No | WT | 2 copies | No | No | No | Yes | Yes | Yes | Yes | No |
| Case-329 | Male | 57 | Relapse | non-GCB | II | 0 | - | No | WT | 2 copies | No | No | No | Yes | No | Yes | Yes | No |
| Case-428 | Male | 57 | Primary | non-GCB | I | 0 | - | NA | NA | NA | No | No | No | Yes | No | No | No | No |
| Case-440 | Male | 57 | Primary | GCB | II | 1 | - | NA | WT | 2 copies | No | No | No | Yes | No | Yes | No | No |
| Case-34 | Male | 58 | Primary | GCB | IV | 3 | - | No | WT | 2 copies | No | Yes | No | No | No | Yes | Yes | Yes |
| Case-52 | Male | 58 | Primary | non-GCB | II | 0 | - | No | WT | 2 copies | No | Yes | No | No | Yes | Yes | Yes | Yes |
| Case-91 | Male | 58 | Primary | GCB | II | 1 | - | No | WT | 2 copies | No | No | No | Yes | Yes | Yes | Yes | No |
| Case-330 | Male | 58 | Primary | non-GCB | NA | NA | - | No | non-WT | 2 copies | Mutation | No | No | Yes | No | Yes | Yes | No |
| Case-378 | Male | 58 | Primary | GCB | II | 1 | - | NA | WT | 2 copies | No | No | No | Yes | No | Yes | Yes | No |
| Case-489 | Male | 58 | Primary | GCB | II | 1 | - | No | NA | NA | No | No | No | No | Yes | No | Yes | No |
| Case-504 | Male | 58 | Primary | non-GCB | II | 0 | NA | No | WT | 2 copies | No | No | No | No | Yes | Yes | Yes | No |
| Case-116 | Male | 59 | Primary | non-GCB | IV | 3 | - | No | non-WT | 2 copies | Mutation | No | Yes | No | No | Yes | Yes | No |
| Case-135 | Male | 59 | Primary | non-GCB | III | 0 | + | Yes | WT | 2 copies | No | No | No | Yes | Yes | Yes | Yes | Yes |
| Case-314 | Male | 59 | Primary | non-GCB | NA | NA | - | No | NA | NA | No | No | No | Yes | No | No | Yes | No |
| Case-392 | Male | 59 | Primary | non-GCB | II | 2 | - | NA | non-WT | 2 copies | Mutation | No | No | Yes | No | Yes | No | No |
| Case-412 | Male | 59 | Primary | GCB | I | 1 | - | NA | NA | NA | No | No | No | Yes | No | No | Yes | No |
| Case-422 | Male | 59 | Primary | GCB | II | 2 | - | NA | NA | NA | No | No | No | Yes | No | No | No | No |
| Case-453 | Male | 59 | Primary | GCB | IV | 2 | - | NA | WT | 2 copies | No | No | No | Yes | No | Yes | No | No |
| Case-469 | Male | 59 | Relapse | GCB | IV | 3 | + | Yes | WT | 2 copies | No | No | Yes | No | Yes | Yes | No | Yes |
| Case-470 | Male | 59 | Primary | non-GCB | II | 0 | - | No | WT | 2 copies | No | No | Yes | No | Yes | Yes | No | Yes |
| Case-109 | Male | 60 | Primary | GCB | IV | 4 | - | No | WT | 2 copies | No | No | Yes | No | No | Yes | Yes | No |
| Case-340 | Male | 60 | Primary | non-GCB | II | 3 | - | No | non-WT | 2 copies | Mutation | No | No | Yes | Yes | Yes | Yes | No |
| Case-450 | Male | 60 | Primary | GCB | I | 1 | - | NA | WT | 2 copies | No | No | No | Yes | No | Yes | Yes | No |
| Case-496 | Male | 60 | Primary | NA | IV | 5 | NA | No | NA | NA | No | No | No | No | Yes | No | Yes | No |
| Case-9 | Male | 61 | Primary | GCB | IV | 4 | + | No | non-WT | 2 copies | Mutation | Yes | No | No | Yes | Yes | Yes | No |
| Case-19 | Male | 61 | Primary | GCB | III | 2 | - | No | non-WT | 1 copy | No | Yes | No | No | No | Yes | Yes | No |
| Case-29 | Male | 61 | Primary | non-GCB | III | 3 | - | NA | NA | NA | Mutation | Yes | No | No | Yes | No | Yes | Yes |
| Case-126 | Male | 61 | Relapse | GCB | III | 4 | - | No | WT | 2 copies | No | No | Yes | No | Yes | Yes | Yes | No |
| Case-130 | Male | 61 | Primary | non-GCB | IV | 5 | - | No | WT | 2 copies | No | No | Yes | No | Yes | Yes | Yes | No |
| Case-292 | Male | 61 | Primary | non-GCB | IV | 4 | - | No | WT | 2 copies | No | No | No | Yes | Yes | Yes | Yes | No |
| Case-307 | Male | 61 | Primary | GCB | III | 0 | + | NA | WT | 2 copies | No | No | No | Yes | No | Yes | Yes | No |

| Case-312 | Male | 61 | Primary | non-GCB | III | 4 | + | NA | NA | NA | No | No | No | Yes | No | No | Yes | No |
| --- | --- | --- | --- | --- | --- | --- | --- | --- | --- | --- | --- | --- | --- | --- | --- | --- | --- | --- |
| Case-331 | Male | 61 | Primary | GCB | NA | NA | - | No | NA | NA | No | No | No | Yes | No | No | Yes | No |
| Case-399 | Male | 61 | Primary | non-GCB | II | 2 | - | NA | WT | 2 copies | No | No | No | Yes | No | Yes | Yes | No |
| Case-415 | Male | 61 | Primary | non-GCB | II | 2 | - | NA | WT | 2 copies | No | No | No | Yes | No | Yes | Yes | No |
| Case-505 | Male | 61 | Primary | NA | NA | NA | NA | NA | WT | 2 copies | No | No | No | No | Yes | Yes | Yes | No |
| Case-14 | Male | 62 | Primary | non-GCB | IV | 2 | - | No | WT | 2 copies | No | Yes | No | No | No | Yes | Yes | No |
| Case-115 | Male | 62 | Primary | non-GCB | IV | 4 | - | No | WT | 2 copies | No | No | Yes | No | No | Yes | Yes | No |
| Case-129 | Male | 62 | Primary | non-GCB | IV | 4 | - | NA | WT | 2 copies | No | No | No | Yes | Yes | Yes | Yes | No |
| Case-271 | Male | 62 | Relapse | non-GCB | II | 0 | - | No | non-WT | 1 copy | No | No | No | Yes | Yes | Yes | Yes | No |
| Case-305 | Male | 62 | Primary | non-GCB | III | 3 | + | NA | WT | 2 copies | No | No | No | Yes | No | Yes | Yes | No |
| Case-400 | Male | 62 | Primary | GCB | IV | 3 | - | NA | WT | 2 copies | No | No | No | Yes | No | Yes | No | No |
| Case-404 | Male | 62 | Primary | non-GCB | II | 2 | - | NA | WT | 2 copies | No | No | No | Yes | No | Yes | Yes | No |
| Case-451 | Male | 62 | Relapse | non-GCB | I | 2 | - | NA | WT | 2 copies | No | No | No | Yes | No | Yes | No | No |
| Case-467 | Male | 62 | Relapse | non-GCB | II | 1 | - | No | WT | 2 copies | No | No | No | Yes | Yes | Yes | No | Yes |
| Case-6 | Male | 63 | Primary | GCB | III | 3 | + | No | NA | NA | No | Yes | No | No | No | No | No | Yes |
| Case-417 | Male | 63 | Primary | GCB | IV | 2 | - | NA | WT | 2 copies | No | No | No | Yes | No | Yes | Yes | No |
| Case-10 | Male | 64 | Primary | non-GCB | II | 2 | - | No | WT | 2 copies | No | Yes | No | No | No | Yes | Yes | Yes |
| Case-108 | Male | 64 | Primary | GCB | IV | 3 | - | No | WT | 2 copies | No | No | Yes | No | No | Yes | Yes | No |
| Case-111 | Male | 64 | Primary | GCB | IV | 5 | - | No | WT | 2 copies | No | No | Yes | No | No | Yes | Yes | No |
| Case-407 | Male | 64 | Primary | non-GCB | I | 1 | - | NA | WT | 2 copies | No | No | No | Yes | No | Yes | No | No |
| Case-463 | Male | 64 | Relapse | GCB | II | 1 | - | No | non-WT | 2 copies | Mutation | No | No | Yes | Yes | Yes | Yes | No |
| Case-477 | Male | 64 | Primary | non-GCB | II | 1 | - | No | WT | 2 copies | No | No | No | Yes | Yes | Yes | No | No |
| Case-13 | Male | 65 | Primary | non-GCB | IV | 5 | - | No | NA | NA | Mutation | Yes | No | No | Yes | No | Yes | Yes |
| Case-15 | Male | 65 | Primary | GCB | II | 3 | - | Yes | non-WT | 2 copies | Mutation | Yes | Yes | No | Yes | Yes | No | Yes |
| Case-21 | Male | 65 | Primary | non-GCB | II | 2 | - | No | WT | 2 copies | No | Yes | No | No | No | Yes | Yes | No |
| Case-27 | Male | 65 | Primary | GCB | IV | 4 | - | No | NA | NA | No | Yes | No | No | No | No | No | Yes |
| Case-334 | Male | 65 | Primary | non-GCB | IV | 5 | - | No | non-WT | 2 copies | Mutation | No | No | Yes | Yes | Yes | Yes | No |
| Case-395 | Male | 65 | Primary | non-GCB | IV | 3 | - | NA | non-WT | 2 copies | Mutation | No | No | Yes | No | Yes | Yes | No |
| Case-410 | Male | 65 | Primary | non-GCB | I | 1 | - | NA | NA | NA | No | No | No | Yes | No | No | No | No |
| Case-86 | Male | 66 | Primary | GCB | IV | 2 | + | NA | NA | NA | No | No | No | Yes | Yes | No | Yes | No |
| Case-95 | Male | 66 | Primary | non-GCB | IV | 4 | - | No | WT | 2 copies | No | No | Yes | No | Yes | Yes | Yes | Yes |
| Case-322 | Male | 66 | Primary | non-GCB | NA | NA | - | No | WT | 2 copies | No | No | No | Yes | No | Yes | Yes | No |
| Case-380 | Male | 66 | Primary | non-GCB | NA | NA | - | NA | WT | 2 copies | No | No | No | Yes | No | Yes | No | No |
| Case-398 | Male | 66 | Primary | GCB | I | 1 | - | NA | WT | 2 copies | No | No | No | Yes | No | Yes | Yes | No |
| Case-402 | Male | 66 | Primary | GCB | IV | 3 | - | NA | WT | 2 copies | No | No | No | Yes | No | Yes | Yes | No |
| Case-462 | Male | 66 | Relapse | non-GCB | III | 3 | - | No | WT | 2 copies | No | No | No | Yes | Yes | Yes | Yes | No |
| Case-308 | Male | 67 | Relapse | non-GCB | III | 3 | - | NA | NA | NA | No | No | No | Yes | No | No | Yes | No |
| Case-274 | Male | 68 | Primary | non-GCB | III | 1 | - | No | NA | NA | Mutation | No | No | Yes | Yes | No | Yes | No |
| Case-379 | Male | 68 | Primary | non-GCB | II | 0 | - | NA | WT | 2 copies | No | No | No | Yes | No | Yes | No | No |
| Case-430 | Male | 68 | Primary | GCB | I | 1 | - | NA | non-WT | 2 copies | Mutation | No | No | Yes | No | Yes | No | No |
| Case-433 | Male | 68 | Primary | non-GCB | II | 1 | - | NA | WT | 2 copies | No | No | No | Yes | No | Yes | No | No |
| Case-35 | Male | 69 | Relapse | non-GCB | IV | 3 | - | No | non-WT | 1 copy | No | Yes | No | No | No | Yes | Yes | No |
| Case-105 | Male | 69 | Primary | GCB | III | 3 | - | No | WT | 2 copies | No | No | Yes | No | No | Yes | Yes | No |
| Case-121 | Male | 69 | Primary | non-GCB | II | 1 | - | No | NA | NA | No | No | Yes | No | No | No | Yes | No |
| Case-375 | Male | 69 | Primary | non-GCB | II | 2 | - | NA | WT | 2 copies | No | No | No | Yes | No | Yes | No | No |
| Case-370 | Male | 70 | Primary | non-GCB | III | 3 | - | NA | WT | 2 copies | No | No | No | Yes | No | Yes | No | No |
| Case-403 | Male | 70 | Primary | non-GCB | IV | 5 | - | NA | non-WT | 2 copies | Mutation | No | No | Yes | No | Yes | No | No |
| Case-454 | Male | 70 | Primary | non-GCB | III | 2 | - | NA | WT | 2 copies | No | No | No | Yes | No | Yes | No | No |
| Case-466 | Male | 70 | Relapse | non-GCB | IV | 5 | - | No | WT | 2 copies | No | No | Yes | No | Yes | Yes | No | Yes |
| Case-501 | Male | 70 | Relapse | non-GCB | IV | 5 | + | No | WT | 2 copies | No | No | No | No | Yes | Yes | No | No |
| Case-110 | Male | 71 | Primary | non-GCB | II | 1 | - | No | WT | 2 copies | No | No | Yes | No | No | Yes | Yes | No |
| Case-420 | Male | 71 | Relapse | NA | I | 1 | - | No | WT | 2 copies | No | No | No | Yes | Yes | Yes | Yes | No |
| Case-24 | Male | 72 | Primary | GCB | III | 2 | - | No | NA | NA | No | Yes | No | No | No | No | Yes | Yes |
| Case-310 | Male | 73 | Primary | non-GCB | NA | NA | - | No | WT | 2 copies | No | No | No | Yes | No | Yes | Yes | No |
| Case-491 | Male | 73 | Primary | GCB | I | 2 | - | No | NA | NA | No | No | No | No | Yes | No | Yes | No |
| Case-294 | Male | 74 | Primary | non-GCB | II | 3 | - | No | WT | 2 copies | No | No | No | Yes | Yes | Yes | Yes | No |
| Case-447 | Male | 74 | Primary | GCB | IV | 4 | - | NA | WT | 2 copies | No | No | No | Yes | No | Yes | No | No |
| Case-33 | Male | 75 | Primary | GCB | IV | 4 | - | No | WT | 2 copies | No | Yes | No | No | No | Yes | Yes | Yes |
| Case-435 | Male | 75 | Primary | GCB | II | 2 | - | NA | WT | 2 copies | No | No | No | Yes | No | Yes | Yes | No |

| Case-445 | Male | 75 | Primary | GCB | IV | 5 | - | NA | WT | 2 copies | No | No | No | Yes | No | Yes | Yes | No |
| --- | --- | --- | --- | --- | --- | --- | --- | --- | --- | --- | --- | --- | --- | --- | --- | --- | --- | --- |
| Case-47 | Male | 76 | Primary | non-GCB | II | 1 | - | No | WT | 2 copies | No | Yes | No | No | No | Yes | Yes | Yes |
| Case-458 | Male | 76 | Relapse | GCB | III | 3 | - | NA | WT | 2 copies | No | No | Yes | No | Yes | Yes | No | Yes |
| Case-94 | Male | 78 | Primary | non-GCB | II | 2 | - | No | non-WT | 2 copies | Mutation | No | Yes | No | Yes | Yes | Yes | No |
| Case-275 | Male | 81 | Primary | non-GCB | II | 3 | - | NA | NA | NA | No | No | No | Yes | Yes | No | Yes | No |
| Case-419 | Male | 81 | Primary | GCB | II | 1 | - | NA | NA | NA | No | No | No | Yes | No | No | No | No |
| Case-485 | Male | 81 | Primary | GCB | III | 4 | - | NA | non-WT | 1 copy | No | No | No | No | Yes | Yes | Yes | No |
| Case-2 | Male | 83 | Primary | GCB | III | 3 | - | No | non-WT | 2 copies | Mutation | Yes | No | No | No | Yes | Yes | Yes |
| Case-377 | Male | 91 | Primary | GCB | NA | NA | - | NA | WT | 2 copies | No | No | No | Yes | No | Yes | Yes | No |
