# Supplementary material for: The dual role of CD70 in B‐cell lymphomagenesis
Source: Clin Transl Med. 2022 Dec 5;12(12):e1118. doi: 10.1002/ctm2.1118 (PMC9722974; doi:10.1002/ctm2.1118)
Supplement: Supplementary file 3 — Supporting Information [file CTM2-12-e1118-s003.docx]

S2. The experiments performed and clinical data of the Swedish DLBCL cohort.

| **Sample ID.** | **Gender** | **Age** | **Primay/Relapse biopsy** | **Molecular subtype** | **Ann Arbor Stage** | **IPI score** | **EBER** | **CD70 WT/non-WT** | **CD70 Mutation** | **CD70 Copy number** | **WES** | **Lymphochip** | **CD70 Sanger** | **CD70 CNV analysis** | **CD70 IHC staining** |
| --- | --- | --- | --- | --- | --- | --- | --- | --- | --- | --- | --- | --- | --- | --- | --- |
| UL70 | Female | 29 | Primary | non-GCB | 2 | 0 | NA | NA | No | NA | No | No | Yes | No | Yes |
| UL111 | Female | 30 | Primary | non-GCB | 4 | 2 | NA | NA | No | NA | No | No | Yes | No | No |
| UL139 | Female | 32 | Primary | GCB | 1 | 0 | NA | WT | No | 2 copies | No | No | Yes | Yes | Yes |
| LN15 | Female | 33 | Primary | non-GCB | 1 | 0 | NA | NA | No | NA | No | No | Yes | No | No |
| UL52 | Female | 36 | Relapse | non-GCB | 4 | 2 | NA | WT | No | 2 copies | No | No | Yes | Yes | Yes |
| IHC28 | Female | 37 | Primary | GCB | 2 | 0 | No | NA | NA | NA | No | No | No | No | Yes |
| IHC39 | Female | 37 | Primary | GCB | 3 | 1 | No | NA | NA | NA | No | No | No | No | Yes |
| UL123 | Female | 38 | Primary | non-GCB | 4 | 3 | NA | WT | No | 2 copies | No | No | Yes | Yes | No |
| IHC4 | Female | 44 | Primary | GCB | 3 | 1 | No | NA | NA | NA | No | No | No | No | Yes |
| UL92 | Female | 44 | Primary | GCB | 3 | 1 | NA | WT | No | 2 copies | No | No | Yes | Yes | No |
| UL140 | Female | 45 | Primary | GCB | 3 | NA | NA | NA | Yes | NA | No | No | Yes | No | Yes |
| IHC29 | Female | 45 | Primary | GCB | 1 | 0 | No | NA | NA | NA | No | No | No | No | Yes |
| UL106 | Female | 47 | Primary | GCB | 4 | 3 | NA | WT | No | 2 copies | No | No | Yes | Yes | No |
| UL149 | Female | 47 | Relapse | GCB | 4 | 2 | NA | NA | No | NA | No | No | Yes | No | No |
| UL34 | Female | 48 | Primary | GCB | 3 | 1 | NA | NA | No | NA | No | No | Yes | No | Yes |
| IHC79 | Female | 48 | Primary | GCB | 4 | 2 | NA | NA | NA | NA | No | No | No | No | Yes |
| IHC90 | Female | 49 | Primary | non-GCB | 1 | NA | NA | NA | NA | NA | No | No | No | No | Yes |
| IHC6 | Female | 50 | Primary | GCB | 4 | 3 | No | NA | NA | NA | No | No | No | No | Yes |
| UL113 | Female | 50 | Primary | NA | 2 | 1 | NA | non-WT | No | 1 copy | No | No | Yes | Yes | No |
| IHC45 | Female | 51 | Primary | GCB | 4 | 3 | No | NA | NA | NA | No | No | No | No | Yes |
| UL12 | Female | 52 | Primary | NA | 3 | 1 | NA | NA | No | NA | No | Yes | Yes | No | No |
| UL101 | Female | 52 | Primary | non-GCB | 4 | 3 | NA | NA | No | NA | No | No | Yes | No | No |
| UL120 | Female | 52 | Primary | NA | 4 | 2 | NA | WT | No | 2 copies | No | No | Yes | Yes | No |
| UL170 | Female | 52 | Primary | NA | 1 | NA | NA | WT | No | 2 copies | No | No | Yes | Yes | No |
| DLS28 | Female | 52 | Primary | GCB | 2 | 1 | NA | NA | No | NA | Yes | No | No | No | No |
| UL5 | Female | 53 | Primary | GCB | 1 | 0 | No | WT | No | 2 copies | No | Yes | Yes | Yes | Yes |
| IHC47 | Female | 53 | Primary | GCB | 4 | NA | No | NA | NA | NA | No | No | No | No | Yes |
| UL156 | Female | 53 | Primary | NA | 1 | 1 | NA | WT | No | 2 copies | No | No | Yes | Yes | No |
| IHC89 | Female | 56 | Primary | non-GCB | 2 | 0 | NA | NA | NA | NA | No | No | No | No | Yes |
| UL66 | Female | 56 | Primary | GCB | 3 | 1 | NA | NA | No | NA | No | No | Yes | No | No |
| UL78 | Female | 58 | Primary | GCB | 2 | 0 | NA | WT | No | 2 copies | No | No | Yes | Yes | Yes |
| UL153 | Female | 58 | Primary | non-GCB | 3 | 3 | NA | NA | No | NA | No | No | Yes | No | No |
| UL74 | Female | 59 | Relapse | GCB | 3 | 2 | NA | WT | No | 2 copies | No | No | Yes | Yes | Yes |
| DLS39 | Female | 59 | Primary | non-GCB | NA | NA | No | NA | No | NA | No | Yes | No | No | Yes |
| IHC41 | Female | 60 | Primary | GCB | 3 | 1 | No | NA | NA | NA | No | No | No | No | Yes |
| TMA-RCHOP-87 | Female | 60 | Primary | GCB | 3 | 2 | No | NA | No | NA | No | No | Yes | No | Yes |
| IHC81 | Female | 60 | Primary | GCB | 2 | 2 | NA | NA | NA | NA | No | No | No | No | Yes |
| UL20 | Female | 61 | Primary | non-GCB | 1 | 1 | NA | WT | No | 2 copies | No | Yes | Yes | Yes | Yes |
| UL24 | Female | 61 | Primary | GCB | 4 | 2 | NA | NA | No | NA | No | Yes | Yes | No | Yes |
| IHC74 | Female | 61 | Primary | non-GCB | 4 | 5 | No | NA | NA | NA | No | No | No | No | Yes |
| IHC84 | Female | 61 | Primary | non-GCB | 2 | NA | NA | NA | NA | NA | No | No | No | No | Yes |
| UL150 | Female | 62 | Primary | GCB | 2 | 1 | NA | NA | No | NA | No | No | Yes | No | No |
| UL172 | Female | 62 | Primary | NA | 4 | 2 | NA | non-WT | Yes | 2 copies | No | No | Yes | Yes | No |
| UL21 | Female | 63 | Primary | GCB | 1 | 1 | No | WT | No | 2 copies | No | Yes | Yes | Yes | Yes |
| IHC18 | Female | 63 | Primary | non-GCB | 4 | 5 | No | NA | NA | NA | No | No | No | No | Yes |
| IHC55 | Female | 63 | Primary | GCB | 4 | 4 | No | NA | NA | NA | No | No | No | No | Yes |
| IHC61 | Female | 63 | Primary | non-GCB | 2 | 1 | No | NA | NA | NA | No | No | No | No | Yes |
| IHC66 | Female | 63 | Primary | non-GCB | 3 | NA | No | NA | NA | NA | No | No | No | No | Yes |
| IHC43 | Female | 64 | Primary | non-GCB | 1 | 1 | No | NA | NA | NA | No | No | No | No | Yes |
| UL137 | Female | 66 | Primary | non-GCB | 1 | 1 | NA | NA | No | NA | No | No | Yes | No | Yes |
| UL63 | Female | 66 | Primary | GCB | 1 | 0 | NA | NA | No | NA | No | No | Yes | No | No |
| DLS21 | Female | 66 | Primary | GCB | 4 | 3 | NA | NA | No | NA | No | Yes | No | No | No |
| UL36 | Female | 67 | Primary | non-GCB | 3 | 2 | NA | NA | No | NA | No | No | Yes | No | Yes |

| UL129 | Female | 67 | Relapse | GCB | 4 | 2 | NA | NA | No | NA | No | No | Yes | No | Yes |
| --- | --- | --- | --- | --- | --- | --- | --- | --- | --- | --- | --- | --- | --- | --- | --- |
| IHC23 | Female | 68 | Primary | non-GCB | 2 | NA | Yes | NA | NA | NA | No | No | No | No | Yes |
| IHC86 | Female | 68 | Primary | GCB | 3 | 3 | NA | NA | NA | NA | No | No | No | No | Yes |
| UL82 | Female | 68 | Primary | NA | 4 | 1 | NA | WT | No | 2 copies | No | No | Yes | Yes | No |
| DLS9 | Female | 68 | Primary | NA | 1 | 2 | NA | NA | No | NA | Yes | No | No | No | No |
| UL23 | Female | 69 | Primary | GCB | 4 | 2 | NA | WT | No | 2 copies | No | Yes | Yes | Yes | Yes |
| UL128 | Female | 69 | Primary | non-GCB | 3 | 5 | Yes | WT | No | 2 copies | No | No | Yes | Yes | Yes |
| UL169 | Female | 69 | Primary | non-GCB | 1 | 1 | No | NA | No | NA | No | No | Yes | No | Yes |
| IHC24 | Female | 69 | Primary | GCB | 1 | 2 | No | NA | NA | NA | No | No | No | No | Yes |
| UL49 | Female | 69 | Primary | NA | 1 | 1 | NA | NA | No | NA | No | No | Yes | No | No |
| DLS10 | Female | 69 | Primary | NA | 4 | 3 | NA | NA | No | NA | Yes | No | No | No | No |
| UL43 | Female | 71 | Primary | non-GCB | 1 | 1 | NA | WT | No | 2 copies | No | No | Yes | Yes | Yes |
| UL104 | Female | 71 | Primary | GCB | 1 | 0 | NA | WT | No | 2 copies | No | No | Yes | Yes | Yes |
| IHC96 | Female | 71 | Primary | non-GCB | 3 | 3 | NA | NA | NA | NA | No | No | No | No | Yes |
| UL80 | Female | 71 | Primary | NA | 1 | 0 | NA | WT | No | 2 copies | No | No | Yes | Yes | No |
| UL114 | Female | 72 | Primary | non-GCB | 4 | 3 | NA | WT | No | 2 copies | No | No | Yes | Yes | Yes |
| IHC13 | Female | 72 | Primary | GCB | 2 | 2 | No | NA | NA | NA | No | No | No | No | Yes |
| UL161 | Female | 72 | Primary | NA | NA | NA | NA | WT | No | 2 copies | No | No | Yes | Yes | No |
| UL9 | Female | 73 | Primary | GCB | 2 | 1 | No | WT | No | 2 copies | No | Yes | Yes | Yes | Yes |
| UL96 | Female | 73 | Primary | GCB | 3 | 2 | NA | NA | Yes | NA | No | No | Yes | No | Yes |
| IHC102 | Female | 73 | Primary | GCB | 3 | 3 | No | NA | NA | NA | No | No | No | No | Yes |
| UL61 | Female | 73 | Primary | GCB | 1 | 1 | NA | NA | No | NA | No | No | Yes | No | No |
| UL79 | Female | 73 | Primary | GCB | 1 | 0 | NA | NA | No | NA | No | No | Yes | No | No |
| IHC44 | Female | 74 | Primary | non-GCB | 3 | 3 | No | NA | NA | NA | No | No | No | No | Yes |
| IHC64 | Female | 74 | Primary | non-GCB | 4 | 3 | No | NA | NA | NA | No | No | No | No | Yes |
| UL122 | Female | 74 | Primary | GCB | 4 | 2 | NA | WT | No | 2 copies | No | No | Yes | Yes | No |
| UL159 | Female | 74 | Primary | NA | 4 | 2 | NA | WT | No | 2 copies | No | No | Yes | Yes | No |
| IHC70 | Female | 75 | Primary | GCB | 3 | 2 | No | NA | NA | NA | No | No | No | No | Yes |
| UL62 | Female | 75 | Primary | non-GCB | 4 | 3 | NA | WT | No | 2 copies | No | No | Yes | Yes | No |
| UL107 | Female | 75 | Primary | GCB | 2 | 1 | NA | WT | No | 2 copies | No | No | Yes | Yes | No |
| UL37 | Female | 76 | Primary | non-GCB | 2 | 0 | NA | non-WT | Yes | 1 copy | No | No | Yes | Yes | Yes |
| IHC65 | Female | 76 | Primary | non-GCB | 3 | 4 | No | NA | NA | NA | No | No | No | No | Yes |
| UL86 | Female | 76 | Primary | GCB | 3 | 3 | NA | NA | No | NA | No | No | Yes | No | No |
| UL10 | Female | 77 | Primary | non-GCB | 3 | 4 | Yes | WT | No | 2 copies | No | Yes | Yes | Yes | Yes |
| UL26 | Female | 77 | Primary | non-GCB | 1 | 0 | NA | non-WT | Yes | 2 copies | No | Yes | Yes | Yes | Yes |
| IHC16 | Female | 77 | Primary | GCB | 4 | 2 | No | NA | NA | NA | No | No | No | No | Yes |
| UL151 | Female | 77 | Primary | NA | 2 | NA | NA | non-WT | No | 1 copy | No | No | Yes | Yes | No |
| UL6 | Female | 79 | Primary | GCB | 2 | 1 | No | non-WT | No | 1 copy | No | Yes | Yes | Yes | Yes |
| UL56 | Female | 79 | Primary | GCB | 3 | 2 | NA | NA | No | NA | No | No | Yes | No | Yes |
| IHC71 | Female | 79 | Primary | non-GCB | 1 | 1 | Yes | NA | NA | NA | No | No | No | No | Yes |
| IHC92 | Female | 79 | Primary | non-GCB | 3 | 2 | NA | NA | NA | NA | No | No | No | No | Yes |
| UL134 | Female | 79 | Primary | GCB | 2 | 0 | NA | WT | No | 2 copies | No | No | Yes | Yes | No |
| UL167 | Female | 79 | Primary | NA | 4 | 3 | NA | WT | No | 2 copies | No | No | Yes | Yes | No |
| UL22 | Female | 80 | Primary | GCB | 4 | 2 | No | NA | No | NA | No | Yes | Yes | No | Yes |
| UL60 | Female | 80 | Primary | non-GCB | 2 | 1 | NA | WT | No | 2 copies | No | No | Yes | Yes | Yes |
| UL152 | Female | 80 | Relapse | non-GCB | 2 | 0 | NA | NA | No | NA | No | No | Yes | No | Yes |
| IHC78 | Female | 80 | Primary | non-GCB | 3 | 3 | NA | NA | NA | NA | No | No | No | No | Yes |
| IHC85 | Female | 80 | Primary | GCB | 1 | 2 | NA | NA | NA | NA | No | No | No | No | Yes |
| UL41 | Female | 80 | Primary | NA | 2 | 1 | NA | NA | No | NA | No | No | Yes | No | No |
| UL45 | Female | 80 | Primary | NA | 2 | 1 | NA | NA | No | NA | No | No | Yes | No | No |
| UL97 | Female | 81 | Primary | non-GCB | 2 | 1 | NA | WT | No | 2 copies | No | No | Yes | Yes | Yes |
| IHC1 | Female | 81 | Primary | GCB | 1 | 3 | No | NA | NA | NA | No | No | No | No | Yes |
| UL89 | Female | 82 | Primary | non-GCB | 4 | 3 | NA | WT | No | 2 copies | No | No | Yes | Yes | Yes |
| UL88 | Female | 82 | Primary | NA | 2 | 2 | NA | WT | No | 2 copies | No | No | Yes | Yes | No |
| UL2 | Female | 83 | Primary | GCB | 1 | 1 | No | NA | No | NA | No | Yes | Yes | No | Yes |
| DLS25 | Female | 84 | Primary | GCB | 3 | 3 | No | NA | No | NA | No | Yes | No | No | Yes |
| UL174 | Female | 85 | Primary | non-GCB | NA | NA | NA | WT | No | 2 copies | No | No | Yes | Yes | Yes |

| IHC93 | Female | 85 | Primary | non-GCB | 2 | 3 | NA | NA | NA | NA | No | No | No | No | Yes |
| --- | --- | --- | --- | --- | --- | --- | --- | --- | --- | --- | --- | --- | --- | --- | --- |
| LN16 | Female | 87 | Primary | non-GCB | 2 | 2 | NA | NA | No | NA | No | No | Yes | No | No |
| UL168 | Female | 90 | Primary | non-GCB | 1 | 1 | NA | WT | No | 2 copies | No | No | Yes | Yes | Yes |
| UL3 | Male | 48 | Primary | non-GCB | 2 | 1 | No | NA | No | NA | No | Yes | Yes | No | Yes |
| UL32 | Male | 17 | Primary | GCB | 1 | 1 | No | WT | No | 2 copies | No | Yes | Yes | Yes | Yes |
| UL76 | Male | 17 | Primary | non-GCB | 1 | 0 | NA | WT | No | 2 copies | No | No | Yes | Yes | Yes |
| UL55 | Male | 18 | Primary | NA | 1 | 1 | NA | NA | No | NA | No | No | Yes | No | No |
| UL118 | Male | 31 | Primary | non-GCB | 2 | 2 | NA | NA | No | NA | No | No | Yes | No | No |
| UL38 | Male | 36 | Primary | non-GCB | 1 | 0 | NA | WT | No | 2 copies | No | No | Yes | Yes | Yes |
| UL160 | Male | 36 | Primary | NA | 3 | 2 | NA | WT | No | 2 copies | No | No | Yes | Yes | No |
| DLS33 | Male | 36 | Primary | non-GCB | 1 | 0 | NA | NA | No | NA | No | Yes | No | No | No |
| UL15 | Male | 38 | Primary | non-GCB | 4 | 3 | No | WT | No | 2 copies | No | Yes | Yes | Yes | Yes |
| IHC54 | Male | 39 | Primary | GCB | 3 | 2 | No | NA | NA | NA | No | No | No | No | Yes |
| UL165 | Male | 39 | Primary | NA | 1 | 0 | NA | WT | No | 2 copies | No | No | Yes | Yes | No |
| DLS38 | Male | 39 | Primary | GCB | 3 | 2 | NA | NA | No | NA | No | Yes | No | No | No |
| UL105 | Male | 42 | Primary | non-GCB | 1 | 1 | NA | NA | No | NA | No | No | Yes | No | Yes |
| IHC52 | Male | 42 | Primary | non-GCB | 4 | 2 | Yes | NA | NA | NA | No | No | No | No | Yes |
| UL154 | Male | 43 | Relapse | non-GCB | 4 | 2 | NA | NA | No | NA | No | No | Yes | No | Yes |
| IHC53 | Male | 44 | Primary | GCB | 2 | 0 | No | NA | NA | NA | No | No | No | No | Yes |
| UL116 | Male | 44 | Primary | GCB | 2 | 0 | NA | NA | No | NA | No | No | Yes | No | No |
| IHC62 | Male | 45 | Primary | GCB | 1 | 1 | No | NA | NA | NA | No | No | No | No | Yes |
| DLS36 | Male | 45 | Primary | GCB | 4 | 2 | NA | NA | No | NA | No | Yes | No | No | No |
| UL33 | Male | 47 | Primary | GCB | 4 | 2 | NA | WT | No | 2 copies | No | No | Yes | Yes | Yes |
| IHC101 | Male | 47 | Primary | GCB | 2 | 1 | NA | NA | NA | NA | No | No | No | No | Yes |
| UL31 | Male | 48 | Primary | non-GCB | 1 | 1 | NA | NA | No | NA | No | No | Yes | No | Yes |
| DLS37 | Male | 48 | Primary | GCB | 3 | 2 | No | NA | No | NA | No | Yes | No | No | Yes |
| UL28 | Male | 49 | Primary | GCB | 1 | 0 | No | WT | No | 2 copies | No | No | Yes | Yes | Yes |
| IHC76 | Male | 49 | Primary | GCB | 3 | 1 | NA | NA | NA | NA | No | No | No | No | Yes |
| IHC91 | Male | 49 | Primary | GCB | 4 | 4 | NA | NA | NA | NA | No | No | No | No | Yes |
| UL84 | Male | 49 | Relapse | NA | 4 | 1 | NA | WT | No | 2 copies | No | No | Yes | Yes | No |
| TMA-22-11 | Male | 50 | Primary | non-GCB | 1 | 1 | No | NA | No | NA | No | No | Yes | No | Yes |
| IHC36 | Male | 50 | Primary | GCB | 1 | 0 | No | NA | NA | NA | No | No | No | No | Yes |
| IHC82 | Male | 50 | Primary | non-GCB | 3 | 2 | NA | NA | NA | NA | No | No | No | No | Yes |
| UL29 | Male | 52 | Primary | non-GCB | 2 | 0 | No | WT | No | 2 copies | No | Yes | Yes | Yes | Yes |
| UL143 | Male | 52 | Primary | GCB | 2 | 0 | NA | WT | No | 2 copies | No | No | Yes | Yes | Yes |
| UL197 | Male | 52 | Primary | NA | 4 | 3 | NA | NA | No | NA | No | No | Yes | No | Yes |
| UL108 | Male | 52 | Primary | GCB | 3 | 2 | NA | NA | No | NA | No | No | Yes | No | No |
| UL124 | Male | 52 | Relapse | NA | 2 | NA | NA | NA | No | NA | No | No | Yes | No | No |
| UL125 | Male | 52 | Primary | NA | 2 | NA | NA | WT | No | 2 copies | No | No | Yes | Yes | No |
| DLS35 | Male | 52 | Primary | GCB | 4 | 3 | NA | NA | No | NA | No | Yes | No | No | No |
| UL51 | Male | 53 | Primary | GCB | 4 | 3 | NA | WT | No | 2 copies | No | No | Yes | Yes | Yes |
| IHC48 | Male | 53 | Primary | non-GCB | 3 | 2 | Yes | NA | NA | NA | No | No | No | No | Yes |
| DLS34 | Male | 54 | Primary | non-GCB | NA | NA | No | NA | No | NA | No | Yes | No | No | Yes |
| IHC58 | Male | 54 | Primary | non-GCB | 1 | 1 | No | NA | NA | NA | No | No | No | No | Yes |
| IHC14 | Male | 55 | Primary | GCB | 3 | 2 | No | NA | NA | NA | No | No | No | No | Yes |
| UL100 | Male | 56 | Primary | GCB | 3 | 2 | NA | NA | No | NA | No | No | Yes | No | Yes |
| TMA-RCHOP-21 | Male | 57 | Primary | non-GCB | 2 | 0 | No | NA | No | NA | No | No | Yes | No | Yes |
| UL93 | Male | 57 | Primary | GCB | 1 | 0 | NA | WT | No | 2 copies | No | No | Yes | Yes | No |
| DLS32 | Male | 57 | Primary | GCB | 4 | 2 | NA | NA | No | NA | Yes | No | No | No | No |
| IHC10 | Male | 59 | Primary | GCB | 1 | 2 | No | NA | NA | NA | No | No | No | No | Yes |
| IHC32 | Male | 59 | Primary | non-GCB | 4 | NA | No | NA | NA | NA | No | No | No | No | Yes |
| DLS16 | Male | 60 | Primary | non-GCB | 1 | 1 | No | NA | No | NA | No | Yes | No | No | Yes |
| UL71 | Male | 60 | Primary | non-GCB | 2 | 0 | NA | NA | No | NA | No | No | Yes | No | No |
| IHC37 | Male | 61 | Primary | non-GCB | 4 | 3 | Yes | NA | NA | NA | No | No | No | No | Yes |
| UL27 | Male | 62 | Primary | non-GCB | 1 | 2 | No | WT | No | 2 copies | Yes | No | Yes | Yes | Yes |
| UL77 | Male | 62 | Primary | GCB | 3 | 2 | NA | WT | No | 2 copies | No | No | Yes | Yes | Yes |
| UL69 | Male | 62 | Primary | non-GCB | 1 | 0 | NA | NA | No | NA | No | No | Yes | No | No |

| UL16 | Male | 63 | Primary | NA | 1 | 1 | NA | WT | No | 2 copies | No | Yes | Yes | Yes | Yes |
| --- | --- | --- | --- | --- | --- | --- | --- | --- | --- | --- | --- | --- | --- | --- | --- |
| DLS13 | Male | 63 | Primary | GCB | 4 | 5 | Yes | NA | No | NA | No | Yes | No | No | Yes |
| IHC9 | Male | 63 | Primary | non-GCB | 4 | 3 | No | NA | NA | NA | No | No | No | No | Yes |
| IHC51 | Male | 63 | Primary | GCB | 3 | 3 | No | NA | NA | NA | No | No | No | No | Yes |
| UL25 | Male | 64 | Primary | non-GCB | NA | NA | No | NA | No | NA | No | Yes | Yes | No | Yes |
| IHC17 | Male | 64 | Primary | non-GCB | 3 | 3 | No | NA | NA | NA | No | No | No | No | Yes |
| IHC40 | Male | 64 | Primary | non-GCB | 1 | NA | No | NA | NA | NA | No | No | No | No | Yes |
| IHC50 | Male | 64 | Primary | non-GCB | 1 | 2 | No | NA | NA | NA | No | No | No | No | Yes |
| IHC63 | Male | 64 | Primary | GCB | 3 | 3 | No | NA | NA | NA | No | No | No | No | Yes |
| UL102 | Male | 64 | Primary | GCB | 2 | 0 | NA | WT | No | 2 copies | No | No | Yes | Yes | No |
| DLS15 | Male | 64 | Primary | GCB | 1 | NA | NA | NA | No | NA | No | Yes | No | No | No |
| UL4 | Male | 65 | Relapse | non-GCB | NA | NA | No | WT | No | 2 copies | No | Yes | Yes | Yes | Yes |
| UL19 | Male | 65 | Primary | NA | 2 | 1 | NA | WT | No | 2 copies | No | Yes | Yes | Yes | Yes |
| IHC2 | Male | 65 | Primary | non-GCB | 4 | 4 | No | NA | NA | NA | No | No | No | No | Yes |
| IHC33 | Male | 65 | Primary | non-GCB | 1 | 1 | No | NA | NA | NA | No | No | No | No | Yes |
| TMA-RCHOP-81 | Male | 65 | Primary | GCB | NA | NA | No | NA | No | NA | No | No | Yes | No | Yes |
| IHC68 | Male | 65 | Primary | GCB | 4 | 3 | No | NA | NA | NA | No | No | No | No | Yes |
| UL145 | Male | 65 | Primary | NA | 4 | 2 | NA | WT | No | 2 copies | No | No | Yes | Yes | No |
| UL7 | Male | 66 | Primary | non-GCB | NA | NA | No | NA | Yes | NA | No | Yes | Yes | No | Yes |
| IHC22 | Male | 66 | Primary | non-GCB | 3 | 3 | No | NA | NA | NA | No | No | No | No | Yes |
| IHC34 | Male | 66 | Primary | non-GCB | 3 | 3 | No | NA | NA | NA | No | No | No | No | Yes |
| IHC60 | Male | 66 | Primary | GCB | 1 | 1 | No | NA | NA | NA | No | No | No | No | Yes |
| UL112 | Male | 66 | Primary | NA | 1 | 1 | NA | WT | No | 2 copies | No | No | Yes | Yes | No |
| UL158 | Male | 66 | Primary | non-GCB | 1 | 1 | NA | NA | No | NA | No | No | Yes | No | No |
| DLS7 | Male | 67 | Primary | non-GCB | 1 | 1 | No | NA | No | NA | Yes | No | No | No | Yes |
| IHC25 | Male | 67 | Primary | non-GCB | 4 | 3 | No | NA | NA | NA | No | No | No | No | Yes |
| IHC72 | Male | 67 | Primary | non-GCB | 4 | 5 | No | NA | NA | NA | No | No | No | No | Yes |
| UL94 | Male | 68 | Primary | GCB | 4 | 3 | NA | WT | No | 2 copies | No | No | Yes | Yes | Yes |
| UL141 | Male | 68 | Primary | GCB | 4 | 2 | NA | WT | No | 2 copies | No | No | Yes | Yes | Yes |
| IHC11 | Male | 68 | Primary | GCB | 4 | 2 | No | NA | NA | NA | No | No | No | No | Yes |
| IHC15 | Male | 68 | Primary | GCB | 1 | 1 | No | NA | NA | NA | No | No | No | No | Yes |
| UL147 | Male | 68 | Primary | GCB | 1 | 1 | NA | WT | No | 2 copies | No | No | Yes | Yes | No |
| UL173 | Male | 68 | Primary | NA | 3 | 2 | NA | WT | No | 2 copies | No | No | Yes | Yes | No |
| UL17 | Male | 69 | Primary | non-GCB | 4 | 4 | No | WT | No | 2 copies | Yes | No | Yes | Yes | Yes |
| UL148 | Male | 69 | Primary | non-GCB | 3 | 1 | NA | non-WT | No | 1 copy | No | No | Yes | Yes | Yes |
| IHC30 | Male | 69 | Primary | GCB | 4 | 3 | No | NA | NA | NA | No | No | No | No | Yes |
| IHC73 | Male | 69 | Primary | GCB | 1 | 1 | No | NA | NA | NA | No | No | No | No | Yes |
| UL11 | Male | 70 | Primary | non-GCB | 2 | 0 | NA | NA | No | NA | No | Yes | Yes | No | Yes |
| UL103 | Male | 70 | Primary | GCB | 1 | 0 | NA | WT | No | 2 copies | No | No | Yes | Yes | Yes |
| IHC49 | Male | 70 | Primary | GCB | 1 | 2 | Yes | NA | NA | NA | No | No | No | No | Yes |
| IHC77 | Male | 70 | Primary | non-GCB | 2 | NA | NA | NA | NA | NA | No | No | No | No | Yes |
| UL164 | Male | 70 | Primary | NA | 4 | 2 | NA | NA | No | NA | No | No | Yes | No | No |
| UL196 | Male | 70 | Primary | NA | 3 | NA | NA | NA | No | NA | No | No | Yes | No | No |
| UL115 | Male | 71 | Primary | GCB | 3 | 2 | NA | WT | No | 2 copies | No | No | Yes | Yes | Yes |
| UL130 | Male | 71 | Primary | non-GCB | 4 | 2 | NA | NA | No | NA | No | No | Yes | No | Yes |
| UL144 | Male | 71 | Primary | NA | NA | NA | NA | WT | No | 2 copies | No | No | Yes | Yes | Yes |
| UL121 | Male | 71 | Primary | non-GCB | 4 | 2 | NA | NA | No | NA | No | No | Yes | No | No |
| DLS24 | Male | 71 | Primary | NA | 3 | 3 | NA | NA | No | NA | Yes | No | No | No | No |
| UL13 | Male | 72 | Primary | GCB | 3 | 4 | Yes | WT | No | 2 copies | No | No | Yes | Yes | Yes |
| UL171 | Male | 72 | Primary | non-GCB | 4 | 2 | NA | WT | No | 2 copies | No | No | Yes | Yes | Yes |
| IHC46 | Male | 72 | Primary | GCB | 1 | 1 | No | NA | NA | NA | No | No | No | No | Yes |
| UL53 | Male | 72 | Primary | NA | 3 | 2 | NA | NA | No | NA | No | No | Yes | No | No |
| UL95 | Male | 72 | Primary | non-GCB | 4 | 2 | NA | NA | No | NA | No | No | Yes | No | No |
| UL98 | Male | 72 | Primary | non-GCB | 4 | 2 | NA | WT | No | 2 copies | No | No | Yes | Yes | No |
| UL155 | Male | 72 | Primary | NA | 4 | NA | NA | WT | No | 2 copies | No | No | Yes | Yes | No |
| UL157 | Male | 72 | Primary | NA | 4 | NA | NA | NA | No | NA | No | No | Yes | No | No |
| IHC75 | Male | 73 | Primary | non-GCB | 1 | 1 | No | NA | NA | NA | No | No | No | No | Yes |

| UL131 | Male | 74 | Primary | non-GCB | 4 | 3 | NA | NA | NA | NA | No | No | No | No | Yes |
| --- | --- | --- | --- | --- | --- | --- | --- | --- | --- | --- | --- | --- | --- | --- | --- |
| IHC31 | Male | 74 | Primary | non-GCB | 4 | 4 | No | NA | NA | NA | No | No | No | No | Yes |
| UL39 | Male | 74 | Relapse | NA | 4 | 2 | NA | NA | No | NA | No | No | Yes | No | No |
| UL110 | Male | 74 | Primary | NA | 4 | 2 | NA | NA | No | NA | No | No | Yes | No | No |
| UL166 | Male | 74 | Primary | NA | NA | NA | NA | WT | No | 2 copies | No | No | Yes | Yes | No |
| UL8 | Male | 75 | Primary | non-GCB | 4 | 2 | No | non-WT | Yes | 2 copies | No | Yes | Yes | Yes | Yes |
| IHC38 | Male | 75 | Primary | GCB | 3 | 3 | No | NA | NA | NA | No | No | No | No | Yes |
| IHC56 | Male | 75 | Primary | GCB | 3 | 2 | No | NA | NA | NA | No | No | No | No | Yes |
| IHC97 | Male | 75 | Primary | non-GCB | 4 | 3 | NA | NA | NA | NA | No | No | No | No | Yes |
| DLS20 | Male | 75 | Primary | GCB | 2 | 2 | NA | NA | No | NA | No | Yes | No | No | No |
| IHC59 | Male | 76 | Primary | GCB | 4 | 3 | No | NA | NA | NA | No | No | No | No | Yes |
| IHC80 | Male | 76 | Primary | GCB | 1 | 1 | NA | NA | NA | NA | No | No | No | No | Yes |
| IHC83 | Male | 76 | Primary | GCB | 4 | 3 | NA | NA | NA | NA | No | No | No | No | Yes |
| UL81 | Male | 76 | Primary | non-GCB | 1 | 0 | NA | NA | No | NA | No | No | Yes | No | No |
| UL136 | Male | 76 | Primary | GCB | 2 | 1 | NA | NA | No | NA | No | No | Yes | No | No |
| UL14 | Male | 77 | Primary | non-GCB | 4 | 3 | NA | non-WT | Yes | 2 copies | No | Yes | Yes | Yes | Yes |
| UL18 | Male | 77 | Primary | GCB | 1 | 0 | NA | WT | No | 2 copies | No | Yes | Yes | Yes | Yes |
| UL138 | Male | 77 | Primary | non-GCB | NA | NA | No | NA | No | NA | No | No | Yes | No | Yes |
| IHC35 | Male | 77 | Primary | GCB | 4 | 3 | No | NA | NA | NA | No | No | No | No | Yes |
| IHC98 | Male | 77 | Primary | GCB | 2 | 1 | NA | NA | NA | NA | No | No | No | No | Yes |
| IHC99 | Male | 77 | Primary | non-GCB | 1 | 2 | NA | NA | NA | NA | No | No | No | No | Yes |
| UL132 | Male | 78 | Primary | non-GCB | 4 | 4 | No | WT | No | 2 copies | No | No | Yes | Yes | Yes |
| UL162 | Male | 79 | Primary | non-GCB | 3 | 1 | NA | NA | NA | NA | No | No | No | No | Yes |
| IHC20 | Male | 79 | Primary | non-GCB | 4 | 4 | No | NA | NA | NA | No | No | No | No | Yes |
| IHC100 | Male | 79 | Primary | non-GCB | 4 | 3 | NA | NA | NA | NA | No | No | No | No | Yes |
| IHC87 | Male | 80 | Primary | GCB | 1 | NA | NA | NA | NA | NA | No | No | No | No | Yes |
| IHC88 | Male | 80 | Primary | non-GCB | 3 | 2 | NA | NA | NA | NA | No | No | No | No | Yes |
| UL68 | Male | 81 | Primary | non-GCB | 3 | 3 | NA | WT | No | 2 copies | No | No | Yes | Yes | Yes |
| UL91 | Male | 81 | Primary | non-GCB | 4 | 2 | NA | WT | No | 2 copies | No | No | Yes | Yes | Yes |
| IHC95 | Male | 81 | Primary | non-GCB | 4 | 3 | NA | NA | NA | NA | No | No | No | No | Yes |
| DLS26 | Male | 81 | Primary | GCB | 4 | 4 | NA | NA | No | NA | Yes | No | No | No | No |
| IHC5 | Male | 82 | Primary | GCB | 3 | 3 | Yes | NA | NA | NA | No | No | No | No | Yes |
| IHC57 | Male | 82 | Primary | GCB | 1 | 2 | No | NA | NA | NA | No | No | No | No | Yes |
| DLS23 | Male | 83 | Primary | GCB | 1 | 1 | No | NA | No | NA | No | Yes | No | No | Yes |
| IHC8 | Male | 84 | Primary | non-GCB | 1 | 3 | No | NA | NA | NA | No | No | No | No | Yes |
| IHC19 | Male | 84 | Primary | non-GCB | 3 | 4 | No | NA | NA | NA | No | No | No | No | Yes |
| UL109 | Male | 84 | Primary | NA | 2 | 1 | NA | NA | No | NA | No | No | Yes | No | No |
| IHC3 | Male | 85 | Primary | GCB | 4 | 3 | No | NA | NA | NA | No | No | No | No | Yes |
| IHC7 | Male | 88 | Primary | GCB | 4 | 3 | No | NA | NA | NA | No | No | No | No | Yes |
| IHC21 | Male | 88 | Primary | non-GCB | 3 | 5 | No | NA | NA | NA | No | No | No | No | Yes |
| IHC12 | Male | 90 | Primary | GCB | 1 | 2 | No | NA | NA | NA | No | No | No | No | Yes |
| UL30 | Male | NA | Primary | NA | NA | NA | NA | NA | No | NA | No | Yes | Yes | No | No |
| IHC103 | NA | NA | Primary | NA | 4 | 4 | No | NA | NA | NA | No | No | No | No | Yes |
| IHC104 | NA | NA | Primary | NA | 1 | 2 | No | NA | NA | NA | No | No | No | No | Yes |
| IHC105 | NA | NA | Primary | NA | 3 | 2 | No | NA | NA | NA | No | No | No | No | Yes |
| IHC106 | NA | NA | Primary | NA | 1 | 2 | No | NA | NA | NA | No | No | No | No | Yes |
| IHC107 | NA | NA | Primary | NA | 1 | 1 | Yes | NA | NA | NA | No | No | No | No | Yes |
| IHC108 | NA | NA | Primary | NA | 1 | 1 | Yes | NA | NA | NA | No | No | No | No | Yes |
| IHC109 | NA | NA | Primary | NA | 4 | 4 | No | NA | NA | NA | No | No | No | No | Yes |
| IHC110 | NA | NA | Primary | NA | 3 | 4 | No | NA | NA | NA | No | No | No | No | Yes |
| IHC111 | NA | NA | Primary | NA | 3 | 4 | No | NA | NA | NA | No | No | No | No | Yes |
| IHC112 | NA | NA | Primary | NA | 2 | 1 | No | NA | NA | NA | No | No | No | No | Yes |
| IHC113 | NA | NA | Primary | NA | 1 | 1 | No | NA | NA | NA | No | No | No | No | Yes |
| IHC114 | NA | NA | Primary | NA | 4 | 2 | No | NA | NA | NA | No | No | No | No | Yes |
| IHC115 | NA | NA | Primary | NA | 1 | 1 | No | NA | NA | NA | No | No | No | No | Yes |
| IHC116 | NA | NA | Primary | NA | 1 | 1 | No | NA | NA | NA | No | No | No | No | Yes |
| IHC117 | NA | NA | Primary | NA | 1 | 1 | No | NA | NA | NA | No | No | No | No | Yes |

| IHC118 | NA | NA | Primary | NA | 1 | 0 | No | NA | NA | NA | No | No | No | No | Yes |
| --- | --- | --- | --- | --- | --- | --- | --- | --- | --- | --- | --- | --- | --- | --- | --- |
| IHC119 | NA | NA | Primary | NA | 4 | 2 | No | NA | NA | NA | No | No | No | No | Yes |
| IHC120 | NA | NA | Primary | NA | 1 | 1 | No | NA | NA | NA | No | No | No | No | Yes |
| IHC121 | NA | NA | Primary | NA | NA | 0 | No | NA | NA | NA | No | No | No | No | Yes |
| IHC122 | NA | NA | Primary | NA | 4 | 4 | No | NA | NA | NA | No | No | No | No | Yes |
| IHC123 | NA | NA | Primary | NA | 3 | 2 | No | NA | NA | NA | No | No | No | No | Yes |
| IHC124 | NA | NA | Primary | NA | 4 | 5 | No | NA | NA | NA | No | No | No | No | Yes |
| IHC125 | NA | NA | Primary | NA | 1 | NA | No | NA | NA | NA | No | No | No | No | Yes |
| IHC126 | NA | NA | Primary | NA | 1 | 0 | No | NA | NA | NA | No | No | No | No | Yes |
| IHC127 | NA | NA | Primary | NA | 2 | 0 | No | NA | NA | NA | No | No | No | No | Yes |
| IHC128 | NA | NA | Primary | NA | 3 | 3 | No | NA | NA | NA | No | No | No | No | Yes |
| IHC129 | NA | NA | Primary | NA | 4 | 3 | No | NA | NA | NA | No | No | No | No | Yes |
| IHC130 | NA | NA | Primary | NA | 3 | 1 | No | NA | NA | NA | No | No | No | No | Yes |
| IHC131 | NA | NA | Primary | NA | 3 | 1 | No | NA | NA | NA | No | No | No | No | Yes |
| IHC132 | NA | NA | Primary | NA | 3 | 2 | No | NA | NA | NA | No | No | No | No | Yes |
| IHC133 | NA | NA | Primary | NA | 4 | 2 | No | NA | NA | NA | No | No | No | No | Yes |
| IHC134 | NA | NA | Primary | NA | 3 | 3 | Yes | NA | NA | NA | No | No | No | No | Yes |
| IHC135 | NA | NA | Primary | NA | 3 | 2 | Yes | NA | NA | NA | No | No | No | No | Yes |
| IHC136 | NA | NA | Primary | NA | 2 | 2 | No | NA | NA | NA | No | No | No | No | Yes |
| IHC137 | NA | NA | Primary | NA | 4 | 4 | No | NA | NA | NA | No | No | No | No | Yes |
| IHC138 | NA | NA | Primary | NA | 1 | 1 | No | NA | NA | NA | No | No | No | No | Yes |
| IHC139 | NA | NA | Primary | NA | 3 | 3 | No | NA | NA | NA | No | No | No | No | Yes |
| IHC140 | NA | NA | Primary | NA | 1 | 1 | No | NA | NA | NA | No | No | No | No | Yes |
| IHC141 | NA | NA | Primary | NA | 3 | 2 | No | NA | NA | NA | No | No | No | No | Yes |
| IHC142 | NA | NA | Primary | NA | 1 | 0 | No | NA | NA | NA | No | No | No | No | Yes |
| IHC143 | NA | NA | Primary | NA | 2 | 0 | No | NA | NA | NA | No | No | No | No | Yes |
| IHC144 | NA | NA | Primary | NA | 3 | 1 | No | NA | NA | NA | No | No | No | No | Yes |
| IHC145 | NA | NA | Primary | NA | 4 | 2 | No | NA | NA | NA | No | No | No | No | Yes |
| IHC146 | NA | NA | Primary | NA | 4 | 3 | No | NA | NA | NA | No | No | No | No | Yes |
| IHC147 | NA | NA | Primary | NA | 3 | 3 | No | NA | NA | NA | No | No | No | No | Yes |
| IHC148 | NA | NA | Primary | NA | 3 | 2 | No | NA | NA | NA | No | No | No | No | Yes |
| IHC149 | NA | NA | Primary | NA | 4 | 5 | Yes | NA | NA | NA | No | No | No | No | Yes |
| IHC150 | NA | NA | Primary | NA | 4 | 4 | No | NA | NA | NA | No | No | No | No | Yes |
| IHC151 | NA | NA | Primary | NA | 2 | 1 | No | NA | NA | NA | No | No | No | No | Yes |
| IHC152 | NA | NA | Primary | NA | NA | 2 | No | NA | NA | NA | No | No | No | No | Yes |
| IHC153 | NA | NA | Primary | NA | 4 | 4 | Yes | NA | NA | NA | No | No | No | No | Yes |
| IHC154 | NA | NA | Primary | NA | 4 | 3 | No | NA | NA | NA | No | No | No | No | Yes |
| IHC155 | NA | NA | Primary | NA | 3 | 2 | No | NA | NA | NA | No | No | No | No | Yes |
| IHC156 | NA | NA | Primary | NA | 1 | 0 | No | NA | NA | NA | No | No | No | No | Yes |
| IHC157 | NA | NA | Primary | NA | 4 | 2 | Yes | NA | NA | NA | No | No | No | No | Yes |
| IHC158 | NA | NA | Primary | NA | 4 | 3 | No | NA | NA | NA | No | No | No | No | Yes |
| IHC159 | NA | NA | Primary | NA | 2 | 1 | No | NA | NA | NA | No | No | No | No | Yes |
| IHC160 | NA | NA | Primary | NA | 1 | 1 | Yes | NA | NA | NA | No | No | No | No | Yes |
| IHC161 | NA | NA | Primary | NA | 1 | 1 | No | NA | NA | NA | No | No | No | No | Yes |
| IHC162 | NA | NA | Primary | NA | 2 | 0 | No | NA | NA | NA | No | No | No | No | Yes |
| IHC163 | NA | NA | Primary | NA | 3 | 3 | No | NA | NA | NA | No | No | No | No | Yes |
| IHC164 | NA | NA | Primary | NA | NA | 1 | No | NA | NA | NA | No | No | No | No | Yes |
| IHC165 | NA | NA | Primary | NA | 1 | 0 | No | NA | NA | NA | No | No | No | No | Yes |
| IHC166 | NA | NA | Primary | NA | 1 | 1 | No | NA | NA | NA | No | No | No | No | Yes |
| IHC167 | NA | NA | Primary | NA | 1 | 0 | No | NA | NA | NA | No | No | No | No | Yes |
| IHC168 | NA | NA | Primary | NA | 1 | 2 | No | NA | NA | NA | No | No | No | No | Yes |
| IHC169 | NA | NA | Primary | NA | 2 | 0 | No | NA | NA | NA | No | No | No | No | Yes |
| IHC170 | NA | NA | Primary | NA | 1 | 1 | No | NA | NA | NA | No | No | No | No | Yes |
| IHC171 | NA | NA | Primary | NA | 4 | 2 | No | NA | NA | NA | No | No | No | No | Yes |
| IHC172 | NA | NA | Primary | NA | NA | 1 | No | NA | NA | NA | No | No | No | No | Yes |
| IHC173 | NA | NA | Primary | NA | 3 | 2 | No | NA | NA | NA | No | No | No | No | Yes |
| IHC174 | NA | NA | Primary | NA | NA | 0 | No | NA | NA | NA | No | No | No | No | Yes |

| IHC175 | NA | NA | Primary | NA | 3 | 2 | No | NA | NA | NA | No | No | No | No | Yes |
| --- | --- | --- | --- | --- | --- | --- | --- | --- | --- | --- | --- | --- | --- | --- | --- |
| IHC176 | NA | NA | Primary | NA | 2 | 1 | NA | NA | NA | NA | No | No | No | No | Yes |
| IHC177 | NA | NA | Primary | NA | 1 | 0 | No | NA | NA | NA | No | No | No | No | Yes |
| IHC178 | NA | NA | Primary | NA | 3 | 2 | No | NA | NA | NA | No | No | No | No | Yes |
| IHC179 | NA | NA | Primary | NA | 2 | 3 | No | NA | NA | NA | No | No | No | No | Yes |
| IHC180 | NA | NA | Primary | NA | 2 | 0 | No | NA | NA | NA | No | No | No | No | Yes |
| IHC181 | NA | NA | Primary | NA | 4 | 2 | No | NA | NA | NA | No | No | No | No | Yes |
| IHC182 | NA | NA | Primary | NA | 1 | 0 | NA | NA | NA | NA | No | No | No | No | Yes |
| IHC183 | NA | NA | Primary | NA | NA | 0 | Yes | NA | NA | NA | No | No | No | No | Yes |
| IHC184 | NA | NA | Primary | NA | 3 | 1 | No | NA | NA | NA | No | No | No | No | Yes |
| IHC185 | NA | NA | Primary | NA | 3 | 4 | No | NA | NA | NA | No | No | No | No | Yes |
| IHC186 | NA | NA | Primary | NA | NA | 2 | No | NA | NA | NA | No | No | No | No | Yes |
| IHC187 | NA | NA | Primary | NA | 2 | 0 | No | NA | NA | NA | No | No | No | No | Yes |
| IHC188 | NA | NA | Primary | NA | 3 | 2 | No | NA | NA | NA | No | No | No | No | Yes |
| IHC189 | NA | NA | Primary | NA | 1 | 1 | No | NA | NA | NA | No | No | No | No | Yes |
| IHC190 | NA | NA | Primary | NA | NA | 1 | No | NA | NA | NA | No | No | No | No | Yes |
| IHC191 | NA | NA | Primary | NA | 1 | 1 | No | NA | NA | NA | No | No | No | No | Yes |
| IHC192 | NA | NA | Primary | NA | 3 | 2 | Yes | NA | NA | NA | No | No | No | No | Yes |
| IHC193 | NA | NA | Primary | NA | 4 | 2 | No | NA | NA | NA | No | No | No | No | Yes |
| IHC194 | NA | NA | Primary | NA | 2 | 2 | No | NA | NA | NA | No | No | No | No | Yes |
| IHC195 | NA | NA | Primary | NA | 2 | 2 | No | NA | NA | NA | No | No | No | No | Yes |
| IHC196 | NA | NA | Primary | NA | 4 | 2 | No | NA | NA | NA | No | No | No | No | Yes |
| IHC197 | NA | NA | Primary | NA | 3 | 3 | No | NA | NA | NA | No | No | No | No | Yes |
| IHC198 | NA | NA | Primary | NA | 2 | 0 | No | NA | NA | NA | No | No | No | No | Yes |
| IHC199 | NA | NA | Primary | NA | 2 | 1 | No | NA | NA | NA | No | No | No | No | Yes |
| IHC200 | NA | NA | Primary | NA | 3 | 2 | No | NA | NA | NA | No | No | No | No | Yes |
| IHC201 | NA | NA | Primary | NA | 4 | NA | No | NA | NA | NA | No | No | No | No | Yes |
| IHC202 | NA | NA | Primary | NA | 3 | 2 | Yes | NA | NA | NA | No | No | No | No | Yes |
| IHC203 | NA | NA | Primary | NA | NA | 1 | No | NA | NA | NA | No | No | No | No | Yes |
| IHC204 | NA | NA | Primary | NA | 4 | 3 | No | NA | NA | NA | No | No | No | No | Yes |
| IHC205 | NA | NA | Primary | NA | 3 | 3 | No | NA | NA | NA | No | No | No | No | Yes |
| IHC206 | NA | NA | Primary | NA | 2 | 1 | No | NA | NA | NA | No | No | No | No | Yes |
| IHC207 | NA | NA | Primary | NA | NA | 1 | No | NA | NA | NA | No | No | No | No | Yes |
| IHC208 | NA | NA | Primary | NA | 1 | 1 | No | NA | NA | NA | No | No | No | No | Yes |
| IHC209 | NA | NA | Primary | NA | NA | 1 | No | NA | NA | NA | No | No | No | No | Yes |
| IHC210 | NA | NA | Primary | NA | 4 | 2 | No | NA | NA | NA | No | No | No | No | Yes |
| IHC211 | NA | NA | Primary | NA | 3 | 3 | No | NA | NA | NA | No | No | No | No | Yes |
| IHC212 | NA | NA | Primary | NA | NA | 1 | No | NA | NA | NA | No | No | No | No | Yes |
| IHC213 | NA | NA | Primary | NA | 4 | 5 | No | NA | NA | NA | No | No | No | No | Yes |
| IHC214 | NA | NA | Primary | NA | NA | 3 | No | NA | NA | NA | No | No | No | No | Yes |
| IHC215 | NA | NA | Primary | NA | 3 | 3 | No | NA | NA | NA | No | No | No | No | Yes |
| IHC216 | NA | NA | Primary | NA | 3 | 3 | No | NA | NA | NA | No | No | No | No | Yes |
| IHC217 | NA | NA | Primary | NA | 2 | 1 | No | NA | NA | NA | No | No | No | No | Yes |
| IHC218 | NA | NA | Primary | NA | 1 | 1 | No | NA | NA | NA | No | No | No | No | Yes |
| IHC219 | NA | NA | Primary | NA | NA | 1 | No | NA | NA | NA | No | No | No | No | Yes |
| IHC220 | NA | NA | Primary | NA | NA | 4 | No | NA | NA | NA | No | No | No | No | Yes |
| IHC221 | NA | NA | Primary | NA | NA | 1 | No | NA | NA | NA | No | No | No | No | Yes |
| IHC222 | NA | NA | Primary | NA | 4 | 2 | No | NA | NA | NA | No | No | No | No | Yes |
| IHC223 | NA | NA | Primary | NA | NA | 1 | No | NA | NA | NA | No | No | No | No | Yes |
| IHC224 | NA | NA | Primary | NA | 2 | 2 | No | NA | NA | NA | No | No | No | No | Yes |
| IHC225 | NA | NA | Primary | NA | 4 | 1 | No | NA | NA | NA | No | No | No | No | Yes |
| IHC226 | NA | NA | Primary | NA | 2 | 1 | No | NA | NA | NA | No | No | No | No | Yes |
| IHC227 | NA | NA | Primary | NA | NA | 0 | No | NA | NA | NA | No | No | No | No | Yes |
| IHC228 | NA | NA | Primary | NA | 1 | 1 | No | NA | NA | NA | No | No | No | No | Yes |
| IHC229 | NA | NA | Primary | NA | 1 | 0 | No | NA | NA | NA | No | No | No | No | Yes |
| IHC230 | NA | NA | Primary | NA | 4 | 3 | No | NA | NA | NA | No | No | No | No | Yes |
| IHC231 | NA | NA | Primary | NA | 4 | 2 | No | NA | NA | NA | No | No | No | No | Yes |

| IHC232 | NA | NA | Primary | NA | 2 | 0 | No | NA | NA | NA | No | No | No | No | Yes |
| --- | --- | --- | --- | --- | --- | --- | --- | --- | --- | --- | --- | --- | --- | --- | --- |
| IHC233 | NA | NA | Primary | NA | 1 | 1 | No | NA | NA | NA | No | No | No | No | Yes |
| IHC234 | NA | NA | Primary | NA | 4 | 3 | No | NA | NA | NA | No | No | No | No | Yes |
| IHC235 | NA | NA | Primary | NA | NA | NA | NA | NA | NA | NA | No | No | No | No | Yes |
| IHC237 | NA | NA | Primary | NA | NA | NA | NA | NA | NA | NA | No | No | No | No | Yes |
| IHC240 | NA | NA | Primary | NA | NA | NA | NA | NA | NA | NA | No | No | No | No | Yes |
| IHC244 | NA | NA | Primary | NA | NA | NA | NA | NA | NA | NA | No | No | No | No | Yes |
| IHC245 | NA | NA | Primary | NA | NA | NA | NA | NA | NA | NA | No | No | No | No | Yes |
| IHC246 | NA | NA | Primary | NA | NA | NA | NA | NA | NA | NA | No | No | No | No | Yes |
| IHC251 | NA | NA | Primary | NA | NA | NA | NA | NA | NA | NA | No | No | No | No | Yes |
| IHC256 | NA | NA | Primary | NA | NA | NA | NA | NA | NA | NA | No | No | No | No | Yes |
| UL35 | NA | NA | Primary | NA | NA | NA | NA | NA | No | NA | No | No | Yes | No | No |
| UL40 | NA | NA | Primary | NA | NA | NA | NA | NA | No | NA | No | No | Yes | No | No |
| UL42 | NA | NA | Primary | NA | NA | NA | NA | NA | No | NA | No | No | Yes | No | No |
| UL44 | NA | NA | Primary | NA | NA | NA | NA | NA | No | NA | No | No | Yes | No | No |
| UL48 | NA | NA | Primary | NA | NA | NA | NA | NA | No | NA | No | No | Yes | No | No |
| UL50 | NA | NA | Primary | NA | NA | NA | NA | NA | No | NA | No | No | Yes | No | No |
| UL57 | NA | NA | Primary | NA | NA | NA | NA | NA | No | NA | No | No | Yes | No | No |
| UL58 | NA | NA | Primary | NA | NA | NA | NA | NA | No | NA | No | No | Yes | No | No |
| UL59 | NA | NA | Primary | NA | NA | NA | NA | NA | No | NA | No | No | Yes | No | No |
| UL64 | NA | NA | Primary | NA | NA | NA | NA | NA | No | NA | No | No | Yes | No | No |
| UL65 | NA | NA | Primary | NA | NA | NA | NA | NA | No | NA | No | No | Yes | No | No |
| UL67 | NA | NA | Primary | NA | NA | NA | NA | NA | Yes | NA | No | No | Yes | No | No |
| UL72 | NA | NA | Primary | NA | NA | NA | NA | NA | No | NA | No | No | Yes | No | No |
| UL73 | NA | NA | Primary | NA | NA | NA | NA | NA | No | NA | No | No | Yes | No | No |
| UL75 | NA | NA | Primary | NA | NA | NA | NA | NA | No | NA | No | No | Yes | No | No |
| UL83 | NA | NA | Primary | NA | NA | NA | NA | NA | No | NA | No | No | Yes | No | No |
| UL85 | NA | NA | Primary | NA | NA | NA | NA | NA | No | NA | No | No | Yes | No | No |
| UL117 | NA | NA | Primary | NA | NA | NA | NA | NA | No | NA | No | No | Yes | No | No |
| UL127 | NA | NA | Primary | NA | NA | NA | NA | WT | No | 2 copies | No | No | Yes | Yes | No |
| LN10 | NA | NA | Primary | NA | 3 | 4 | NA | NA | No | NA | No | No | Yes | No | No |
| LN20 | NA | NA | Primary | NA | 1 | 0 | NA | NA | No | NA | No | No | Yes | No | No |
