# Supplementary material for: The dual role of CD70 in B‐cell lymphomagenesis
Source: Clin Transl Med. 2022 Dec 5;12(12):e1118. doi: 10.1002/ctm2.1118 (PMC9722974; doi:10.1002/ctm2.1118)
Supplement: Supplementary file 4 — Supporting Information [file CTM2-12-e1118-s002.docx]

| **S3. *CD70* mutations identified in the Chinese DLBCL cohorts.**  AA: amino acid  MSC-CADD_Impact_Pred: Itan Y, et al. Nat Methods 2016;13:109-10. | | | | | | | | | |
| --- | --- | --- | --- | --- | --- | --- | --- | --- | --- |
| **Chinese cohort, *n* = 300 (70 variants in 58 patients)** |  |  |  |  |  |  |  |  |  |
| **Sample ID** | **Subtype** | **Exon** | **Nucleotide change** | **AA change** | **CADD_score** | **MSC-CADD_Impact_Pred** | **Seqencing method** | **Germline/Somatic** | **VAF** |
| **Nonsynonymous missense mutations**  **(*n* = 28)** | | | | | | | | | |
| Case-317 | GCB | exon 2 | c.C173T | p.A58V | 23.3 | high | Lymphochip | Putatively somatic | 64.2% |
| Case-334 | non-GCB | exon 3 | c.T482TC | p.L161P | 23.7 | high | Lymphochip | Putatively somatic | NA |
| Case-336 | non-GCB | exon 2 | c.T185G | p.L62R | 24.5 | high | Lymphochip | Putatively somatic | 77.9% |
| Case-92 | non-GCB | exon 1 | c.T43A | p.Y15N | 0.532 | low | Illumina WES | Somatic | 4.0% |
| Case-341 | GCB | exon 3 | c.T473C | p.L158P | 24.6 | high | Lymphochip | Putatively somatic | 60.1% |
| Case-94 | non-GCB | exon 1 | c.G86A | p.G29D | 5 747 | low | Illumina WES | Somatic | 6.0% |
| Case-97 | non-GCB | exon 3 | c.C533T | p.S178F | 24.4 | high | Illumina WES | Putatively somatic | 32.0% |
| Case-15 | GCB | exon 2 | c.A182C | p.Q61P | 21.8 | high | Illumina WGS, WES | Somatic | 14.2% |
| Case-4 | non-GCB | exon 3 | c.G560A | p.G187E | 25.2 | high | Complete Genomics WGS | Somatic | NA |
| Case-495 | GCB | exon 2 | c.G172A | p.A58T | 23.4 | high | Sanger sequencing | Putatively somatic | NA |
| Case-500 | GCB | exon 2 | c.G196A | p.G66R | 24.5 | high | Sanger sequencing | Somatic | NA |
| Case-457 | GCB | exon 1 | c.T71A | p.V24D | 1 504 | low | Lymphochip | Putatively somatic | 39.6% |
| Case-502 | GCB | exon 3 | c.G398C | p.C133S | 23.6 | high | Sanger sequencing | Somatic | NA |
| Case-134 | GCB | exon 3 | c.C533T | p.S178F | 23.9 | high | Illumina WES | Somatic | 82.4% |
| Case-268 | non-GCB | exon 1 | c.G161A | p.G54E | 12.59 | high | Lymphochip | Putatively somatic | NA |
| Case-9 | GCB | exon 2 | c.C173T | p.A58V | 23.3 | high | Complete Genomics WGS | Putatively somatic | 77.8% |
| Case-26 | non-GCB | exon 2 | c.G172C | p.A58P | 23.2 | high | Complete Genomics WGS | Putatively somatic | NA |
| Case-274 | non-GCB | exon 2 | c.C184G | p.L62V | 19.1 | high | Lymphochip | Putatively somatic | 22.8% |
| Case-282 | non-GCB | exon 3 | c.T400C | p.S134P | 21.9 | high | Lymphochip | Putatively somatic | 44.0% |
| Case-297 | non-GCB | exon 3 | c.T383C | p.L128P | 23.6 | high | Lymphochip | Putatively somatic | 20.0% |
| Case-298 | non-GCB | exon 3 | c.T343G | p.C115G | 23.3 | high | Lymphochip | Putatively somatic | 36.0% |
| Case-32 | GCB | exon 3 | c.G248A | p.R83H | 29 | high | Illumina WGS | Somatic | 37.4% |
| Case-323 | non-GCB | exon 3 | c.A302T | p.D101V | 22.6 | high | Lymphochip | Putatively somatic | 63.5% |
| Case-330 | non-GCB | exon 2 | c.G172A | p.A58T | 23.4 | high | Lymphochip | Putatively somatic | 40.0% |
| Case-395 | non-GCB | exon 3 | c.G388A | p.V130M | 24.2 | high | Lymphochip | Putatively somatic | 51.4% |
| Case-397 | GCB | exon 3 | c.G431A | p.R144H | 26.7 | high | Lymphochip | Putatively somatic | 53.5% |
| Case-411 | non-GCB | exon 3 | c.G560C | p.G187A | 23.9 | high | Lymphochip | Putatively somatic | 32.8% |
| Case-413 | GCB | exon 3 | c.G244A | p.G82S | 26.3 | high | Lymphochip | Putatively somatic | 33.3% |
| **Stop-gain/loss**  **(*n* = 20)** |  |  |  |  |  |  |  |  |  |
| Case-484 | non-GCB | exon 1 | c.C139T | p.Q47* | 32 | high | Sanger sequencing | Putatively somatic | NA |
| Case-132 | non-GCB | exon 1 | c.T45G | p.Y15X | 24.4 | high | Lymphochip | Putatively somatic | 15.9% |
| Case-97 | non-GCB | exon 3 | c.G224A | p.W75X | 36 | high | Illumina WES | Somatic | 28.6% |
| Case-100 | non-GCB | exon 3 | c.C466T | p.Q156X | 36 | high | Illumina WES | Somatic | 27.3% |
| Case-1 | non-GCB | exon 1 | c.T45G | p.Y15X | 24.4 | high | Illumina WGS, WES | Somatic | 48.3% |
| Case-478 | GCB | exon 1 | c.T89A | p.L30X | 33 | high | Lymphochip | Putatively somatic | 40.7% |
| Case-16 | non-GCB | exon 1 | c.C115T | p.Q39X | 24.7 | high | Complete Genomics WGS | Somatic | 64.7% |
| Case-16 | non-GCB | exon 1 | c.T77A | p.L26X | 34 | high | Complete Genomics WGS | Somatic | 35.6% |
| Case-39 | non-GCB | exon 1 | c.T77A | p.L26X | 34 | high | Complete Genomics WGS | Somatic | 35.6% |
| Case-39 | non-GCB | exon 1 | c.C115T | p.Q39X | 24.7 | high | Complete Genomics WGS | Somatic | 64.7% |

| Case-273 | GCB | exon 1 | c.G30A | p.(=) | 11.8 | high | Lymphochip | Putatively somatic | NA |
| --- | --- | --- | --- | --- | --- | --- | --- | --- | --- |
| Case-287 | non-GCB | exon 1 | c.G138A | p.(=) | 11.7 | high | Lymphochip | Putatively somatic | NA |
| Case-12 | non-GCB | exon 1 | c.C139T | p.Q47X | 32 | high | Illumina WGS | Somatic | 85.7% |
| Case-30 | non-GCB | exon 3 | c.C535T | p.R179X | 26.4 | high | Illumina WGS | Somatic | 40.6% |
| Case-116 | non-GCB | exon 2 | c.G165A | p.W55X | 35 | high | Illumina WES | Somatic | 40.6% |
| Case-383 | GCB | exon 1 | c.C136T | p.Q46X | 34 | high | Lymphochip | Putatively somatic | 29.4% |
| Case-383 | GCB | exon 1 | c.C115T | p.Q39X | 24.7 | high | Lymphochip | Putatively somatic | 28.0% |
| Case-392 | non-GCB | exon 1 | c.C139T | p.Q47X | 32 | high | Lymphochip | Putatively somatic | 37.6% |
| Case-411 | non-GCB | exon 1 | c.T45G | p.Y15X | 24.4 | high | Lymphochip | Putatively somatic | 27.3% |
| Case-430 | GCB | exon 1 | c.T45G | p.Y15X | 24.4 | high | Lymphochip | Putatively somatic | 46.6% |
| **Frameshift deletion**  **(*n* = 6)** |  |  |  |  |  |  |  |  |  |
| Case-29 | non-GCB | exon 1 | c.68delTGGTCC CATT | p.L23fs*5 | 22.1 | high | Complete Genomics WGS | Somatic | NA |
| Case-92 | non-GCB | exon 1 | c.45delT | p.Y15Kfs*17 | - | high | Illumina WES | Somatic | 4.0% |
| Case-4 | non-GCB | exon 1 | c.53_59delTCCT GCG | p.V18fs*11 | 23.6 | high | Complete Genomics WGS | Somatic | NA |
| Case-463 | GCB | exon 3 | c.571dupG | p.V191fs* | NA | NA | Lymphochip | Putatively somatic | NA |
| Case-22 | GCB | exon 1 | c.90_96del | p.L30fs | NA | NA | Illumina WGS | Somatic | 62.5% |
| Case-116 | non-GCB | exon 1 | c.107_114del | p.V36fs | NA | NA | Illumina WES | Somatic | 60.9% |
| **Frameshift substitution**  **(*n* = 3)** |  |  |  |  |  |  |  |  |  |
| Case-340 | non-GCB | exon 1 | c.-4_13G | - | NA | NA | Lymphochip | Putatively somatic | 17.2% |
| Case-295 | non-GCB | exon 1 | c.59_66T | - | NA | NA | Lymphochip | Putatively somatic | 48.5% |
| Case-296 | non-GCB | exon 3 | c.234_234delinsGC | - | NA | NA | Lymphochip | Putatively somatic | 26.5% |
| **Splicing site mutations**  **(*n* = 13)** |  |  |  |  |  |  |  |  |  |
| Case-269 | GCB | exon 2 | c.196+1G>C | - | 21.3 | high | Lymphochip | Putatively somatic | 63.8% |
| Case-29 | non-GCB | exon 2 | c.162+1G>A | - | 21.8 | high | Complete Genomics WGS | Somatic | 19.6% |
| Case-337 | non-GCB | exon 2 | c.196+2T>G | Splice site | 17.38 | high | Lymphochip | Putatively somatic | NA |
| Case-8 | non-GCB | exon 2 | c.196+1G>A | - | 22.3 | high | Illumina WGS, WES | Somatic | 55.2% |
| Case-97 | non-GCB | exon 2 | c.196+2T>G | - | 17.38 | high | Illumina WES | Somatic | 37.7% |
| Case-18 | GCB | exon 2 | c.196+1G>A | - | 22.3 | high | Illumina WGS, WES | Somatic | 42.0% |
| Case-13 | non-GCB | exon 2 | c.196+2T>A | Splice site | 18.08 | high | Complete Genomics WGS | Somatic | NA |
| Case-26 | non-GCB | exon 2 | c.196+2T>A | Splice site | 18.08 | high | Complete Genomics WGS | Somatic | NA |
| Case-274 | non-GCB | exon 2 | c.196+2T>A | - | 18.08 | high | Lymphochip | Putatively somatic | 48.9% |
| Case-88 | non-GCB | exon 2 | c.162+2T>C | - | 21 | high | Lymphochip | Putatively somatic | 38.9% |
| Case-2 | GCB | exon 2 | c.196+2T>A | - | 18.08 | high | Illumina WGS | Somatic | 66.7% |
| Case-11 | GCB | exon 2 | c.195A>G | Splice site | 22.6 | high | Illumina WGS | Somatic | 75.0% |
| Case-403 | non-GCB | exon 2 | c.196+1G>A | - | 22.3 | high | Lymphochip | Putatively somatic | 63.2% |
