# Supplementary material for: The dual role of CD70 in B‐cell lymphomagenesis
Source: Clin Transl Med. 2022 Dec 5;12(12):e1118. doi: 10.1002/ctm2.1118 (PMC9722974; doi:10.1002/ctm2.1118)
Supplement: Supplementary file 5 — Supporting Information [file CTM2-12-e1118-s007.docx]

| **S4. *CD70* mutations identified in the Swedish DLBCL cohorts.** | | | | | | | | |
| --- | --- | --- | --- | --- | --- | --- | --- | --- |
| **Swedish cohort, *n* = 189(10 variants in 189 patients)** | | | | | | | | |
| **Sample ID** | **Subtype** | **Exon** | **Nucleotide change** | **AA change** | **CADD_score** | **MSC-CADD_Impact_Pred** | **Seqencing method** | **Germline/Somatic** |
| **Nonsynonymous missense mutatoins (*n* = 6 )** | | | | | | | | |
| UL7 |  | exon 3 | c.T293G | p.I98S | 10.15 | high | Lymphochip + Sanger | Putatively somatic |
| UL8 |  | exon 3 | c.T473C | p.L158P | 24.1 | high | Lymphochip + Sanger | Putatively somatic |
| UL26 |  | exon 3 | c.G245A | p.G82D | 26.4 | high | Lymphochip + Sanger | Putatively somatic |
| UL37 |  | exon 2 | c.A182C | p.Q61P | 17.52 | high | Sanger sequencing | Putatively somatic |
| UL96 |  | exon 3 | c.G285T | p.Q95H | 7 866 | high | Sanger sequencing | Putatively somatic |
| UL140 |  | exon 3 | c.G204T | p.Q68H |  | high | Sanger sequencing | Putatively somatic |
| **Stop-gain/loss**  **(*n* = 2)** |  |  |  |  |  |  |  |  |
| UL8 |  | exon 3 | c.G224A | p.W75X | 27.6 | high | Sanger sequencing | Putatively somatic |
| UL172 |  | exon 3 | c.C205T | p.Q69X | 35 | high | Sanger sequencing | Putatively somatic |
| **Frameshift substitution (*n* = 1 )** |  |  |  |  |  |  |  |  |
| UL14 |  | exon 1 | c.11_13C | - | NA | NA | Lymphochip + Sanger | Putatively somatic |
| **Non-frameshift substitution (*n* = 1)** | | | | | | | | |
| UL67 |  | exon 2 | c.183_186G | - | NA | NA | Sanger sequencing | Putatively somatic |
