# Supplementary material for: The dual role of CD70 in B‐cell lymphomagenesis
Source: Clin Transl Med. 2022 Dec 5;12(12):e1118. doi: 10.1002/ctm2.1118 (PMC9722974; doi:10.1002/ctm2.1118)
Supplement: Supplementary file 6 — Supporting Information [file CTM2-12-e1118-s005.docx]

| **S5. *CD70* genetic alterations in all cancer types** |
| --- |
| S5a. *CD70* genetic alterations in all cancer types from the COSMIC database |
| S5b*.CD70* genetic alterations in hematopoietic malignancies from the COSMIC database |

| **S5a. *CD70* genetic alterations in all cancer types from the COSMIC database** | | | | |
| --- | --- | --- | --- | --- |
| **Type of cancers** | **Point Mutations** | | **Copy Number Variation** | |
|  | **% Mutated** | **Tested** | **Variant %** | **Tested** |
| Adrenal gland | 0.00 | 627 |  | - |
| Autonomic ganglia | 0.00 | 1137 |  | - |
| Biliary tract | 0.56 | 532 |  | - |
| Bone | 0.18 | 557 |  | - |
| Breast | 0.66 | 2575 | 0.07 | 1492 |
| Central nervous system | 0.16 | 2446 |  | - |
| Cervix | 0.61 | 326 |  | - |
| Endometrium | 0.57 | 706 | 0.17 | 586 |
| Eye | 0.00 | 119 |  | - |
| Fallopian tube | 0.00 | 2 |  | - |
| Gastrointestinal tract (site indeterminate) | 0.00 | 1 |  | - |
| Genital tract | 0.00 | 68 |  | - |
| Haematopoietic and lymphoid | 0.33 | 4253 |  | - |
| Kidney | 0.14 | 2256 | 0.1 | 995 |
| Large intestine | 1.12 | 2314 | 0.14 | 717 |
| Liver | 0.7 | 2157 | 0.15 | 663 |
| Lung | 0.39 | 2548 | 0.1 | 1006 |
| Meninges | 0.00 | 65 |  | - |
| Malignant melanoma | 1.98 | 129 |  | - |
| Oesophagus | 0.53 | 1501 | 0.39 | 510 |
| Ovary | 0.34 | 872 | 0.73 | 684 |
| Pancreas | 0.56 | 1801 | 0.11 | 898 |
| Parathyroid | 0.00 | 35 |  | - |
| Peritoneum | 0.00 | 11 |  | - |
| Pituitary | 0.00 | 57 |  | - |
| Placenta | 0.00 | 2 |  | - |
| Pleura | 0.00 | 251 |  | - |
| Prostate | 0.36 | 1970 |  | - |
| Salivary gland | 0.00 | 98 |  | - |
| Skin | 0.47 | 1274 | 0.34 | 587 |
| Small intestine | 0.00 | 55 |  | - |
| Soft tissue | 0.00 | 561 | 0.76 | 264 |
| Stomach | 0.00 | 846 | 0.21 | 472 |
| Testis | 0.00 | 170 |  | - |
| Thymus | 0.00 | 152 |  | - |
| Thyroid | 0.26 | 1564 |  | - |
| Upper aerodigestive tract | 0.32 | 1257 | 0.77 | 520 |
| Urinary tract | 0.14 | 695 | 0.25 | 399 |

| Vulva | 0.00 | 3 | - |
| --- | --- | --- | --- |

| **S5b*.CD70* genetic alterations in hematopoietic malignancies from the COSMIC database** | | | | | | | |
| --- | --- | --- | --- | --- | --- | --- | --- |
| **Tissue** | **Sub Tissue** | **Histology** | **Sub Histology** | **Point Mutations** | | **Copy Number Variation** | |
|  |  |  |  | **% Mutated** | **Tested** | **Variant %** | **Tested** |
| Haematopoietic and lymphoid | Lymph node | Lymphoid neoplasm | Follicular lymphoma | 0.00 | 11 |  | - |
| Haematopoietic and lymphoid | NS | Lymphoid neoplasm | NK-T cell lymphoma | 0.00 | 135 |  | - |
| Haematopoietic and lymphoid | NS | Haematopoietic neoplasm | Chronic eosinophilic leukaemia-hypereosinophilic syndrome | 0.00 | 1 |  | - |
| Haematopoietic and lymphoid | Soft tissue | Lymphoid neoplasm | Diffuse large B cell lymphoma | 0.00 | 1 |  | - |
| Haematopoietic and lymphoid | NS | Haematopoietic neoplasm | Myelofibrosis | 0.00 | 45 |  | - |
| Haematopoietic and lymphoid | Central nervous system | Lymphoid neoplasm | Primary central nervous system lymphoma | 0.00 | 9 |  | - |
| Haematopoietic and lymphoid | Skin | Lymphoid neoplasm | Mycosis fungoides-Sezary syndrome | 0.00 | 7 |  | - |
| Haematopoietic and lymphoid | NS | Lymphoid neoplasm | Chronic lymphocytic leukaemia-small lymphocytic lymphoma | 0.11 | 915 |  | - |
| Haematopoietic and lymphoid | Mediastinum | Lymphoid neoplasm | Diffuse large B cell lymphoma | 0.00 | 10 |  | - |
| Haematopoietic and lymphoid | Lymph node | Lymphoid neoplasm | Acute lymphoblastic leukaemia | 0.00 | 1 |  | - |
| Haematopoietic and lymphoid | NS | Lymphoid neoplasm | Angioimmunoblastic T cell lymphoma | 0.00 | 88 |  | - |
| Haematopoietic and lymphoid | Breast | Lymphoid neoplasm | Diffuse large B cell lymphoma | 0.00 | 1 |  | - |
| Haematopoietic and lymphoid | NS | Lymphoid neoplasm | Burkitt lymphoma | 0.00 | 60 |  | - |
| Haematopoietic and lymphoid | NS | Haematopoietic neoplasm | Blastic plasmacytoid dendritic cell neoplasm | 0.00 | 3 |  | - |
| Haematopoietic and lymphoid | NS | Haematopoietic neoplasm | Myelodysplastic syndrome | 0.00 | 76 |  | - |
| Haematopoietic and lymphoid | Spleen | Lymphoid neoplasm | Marginal zone lymphoma | 0.00 | 15 |  | - |
| Haematopoietic and lymphoid | NS | Lymphoid neoplasm | Peripheral T cell lymphoma unspecified | 0.00 | 34 |  | - |
| Haematopoietic and lymphoid | Abdomen | Lymphoid neoplasm | Diffuse large B cell lymphoma | 0.00 | 2 |  | - |
| Haematopoietic and lymphoid | Lymph node | Lymphoid neoplasm | Chronic lymphocytic leukaemia-small lymphocytic lymphoma | 0.00 | 2 |  | - |
| Haematopoietic and lymphoid | NS | Lymphoid neoplasm | Plasma cell myeloma | 0.00 | 44 |  | - |
| Haematopoietic and lymphoid | Liver | Lymphoid neoplasm | Burkitt lymphoma | 0.00 | 1 |  | - |
| Haematopoietic and lymphoid | NS | Lymphoid neoplasm | Mantle cell lymphoma | 0.00 | 33 |  | - |
| Haematopoietic and lymphoid | Lymph node | Lymphoid neoplasm | Angioimmunoblastic T cell lymphoma | 0.00 | 5 |  | - |
| Haematopoietic and lymphoid | NS | Haematopoietic neoplasm | Chronic myelomonocytic leukaemia | 0.00 | 27 |  | - |
| Haematopoietic and lymphoid | Lymph node | Lymphoid neoplasm | Mantle cell lymphoma | 0.00 | 8 |  | - |
| Haematopoietic and lymphoid | NS | Haematopoietic neoplasm | Acute lymphoblastic leukaemia | 0.00 | 147 |  | - |
| Haematopoietic and lymphoid | NS | Lymphoid neoplasm | Hairy cell leukaemia | 0.00 | 21 |  | - |
| Haematopoietic and lymphoid | NS | Lymphoid neoplasm | Mixed Langerhans cell histiocytosis-juvenile xanthogranuloma | 0.00 | 5 |  | - |
| Haematopoietic and lymphoid | NS | Haematopoietic neoplasm | Acute myeloid leukaemia therapy related | 0.00 | 43 |  | - |
| Haematopoietic and lymphoid | Small intestine | Lymphoid neoplasm | Enteropathy type T cell lymphoma | 0.00 | 1 |  | - |
| Haematopoietic and lymphoid | Lymph node | Lymphoid neoplasm | NK-T cell lymphoma | 0.00 | 1 |  | - |
| Haematopoietic and lymphoid | NS | Haematopoietic neoplasm | Acute leukaemia | 0.00 | 1 |  | - |
| Haematopoietic and lymphoid | Abdomen | Lymphoid neoplasm | Follicular lymphoma | 0.00 | 1 |  | - |
| Haematopoietic and lymphoid | NS | Haematopoietic neoplasm | Myeloproliferative neoplasm | 0.00 | 4 |  | - |
| Haematopoietic and lymphoid | NS | Haematopoietic neoplasm | Acute leukaemia of ambiguous lineage | 0.00 | 83 |  | - |
| Haematopoietic and lymphoid | Lymph node | Lymphoid neoplasm | NS | 0.00 | 1 |  | - |
| Haematopoietic and lymphoid | NS | Lymphoid neoplasm | Acute lymphoblastic B cell leukaemia | 0.00 | 105 |  | - |
| Haematopoietic and lymphoid | NS | Lymphoid neoplasm | Diffuse large B cell lymphoma | 0.72 | 276 |  | - |
| Haematopoietic and lymphoid | NS | Haematopoietic neoplasm | Chronic myeloid leukaemia | 0.00 | 27 |  | - |
| Haematopoietic and lymphoid | NS | Lymphoid neoplasm | Langerhans cell histiocytosis | 0.00 | 30 |  | - |

| Haematopoietic and lymphoid | NS | Haematopoietic neoplasm | Mast cell neoplasm | 0.00 | 3 | - |
| --- | --- | --- | --- | --- | --- | --- |
| Haematopoietic and lymphoid | NS | Haematopoietic neoplasm | Myelodysplastic syndrome therapy related | 0.00 | 12 | - |
| Haematopoietic and lymphoid | NS | Lymphoid neoplasm | B cell lymphoma unspecified | 0.00 | 9 | - |
| Haematopoietic and lymphoid | NS | Lymphoid neoplasm | Acute lymphoblastic T cell leukaemia | 0.00 | 380 | - |
| Haematopoietic and lymphoid | NS | Lymphoid neoplasm | Acute lymphoblastic leukaemia | 0.00 | 174 | - |
| Haematopoietic and lymphoid | NS | Lymphoid neoplasm | Adult T cell lymphoma-leukaemia | 1.2 | 83 | - |
| Haematopoietic and lymphoid | Spleen | Lymphoid neoplasm | Diffuse large B cell lymphoma | 0.00 | 2 | - |
| Haematopoietic and lymphoid | NS | Lymphoid neoplasm | Follicular lymphoma | 0.00 | 22 | - |
| Haematopoietic and lymphoid | Lymph node | Lymphoid neoplasm | Hodgkin lymphoma | 0.00 | 6 | - |
| Haematopoietic and lymphoid | Tonsil | Lymphoid neoplasm | Diffuse large B cell lymphoma | 0.00 | 1 | - |
| Haematopoietic and lymphoid | Lymph node | Lymphoid neoplasm | Diffuse large B cell lymphoma | 8.7 | 23 | - |
| Haematopoietic and lymphoid | NS | Haematopoietic neoplasm | Myeloproliferative neoplasm unclassifiable | 0.00 | 2 | - |
| Haematopoietic and lymphoid | NS | Haematopoietic neoplasm | Acute myeloid leukaemia | 0.32 | 933 | - |
| Haematopoietic and lymphoid | Lymph node | Lymphoid neoplasm | Acute lymphoblastic B cell leukaemia | 0.00 | 1 | - |
| Haematopoietic and lymphoid | NS | Haematopoietic neoplasm | Acute myeloid leukaemia associated with MDS | 0.00 | 7 | - |
| Haematopoietic and lymphoid | NS | Lymphoid neoplasm | Juvenile xanthogranuloma | 0.00 | 3 | - |
| Haematopoietic and lymphoid | Skin | Lymphoid neoplasm | Anaplastic large cell lymphoma | 0.00 | 2 | - |
| Haematopoietic and lymphoid | NS | Haematopoietic neoplasm | Blast phase chronic myeloid leukaemia | 0.00 | 9 | - |
| Haematopoietic and lymphoid | NS | Haematopoietic neoplasm | Essential thrombocythaemia | 0.00 | 61 | - |
| Haematopoietic and lymphoid | NS | Lymphoid neoplasm | NS | 2.66 | 188 | - |
| Haematopoietic and lymphoid | NS | Haematopoietic neoplasm | Chronic neutrophilic leukaemia | 0.00 | 1 | - |
| Haematopoietic and lymphoid | NS | Haematopoietic neoplasm | Polycythaemia vera | 0.00 | 48 | - |
| Haematopoietic and lymphoid | Skin | Lymphoid neoplasm | Follicular lymphoma | 0.00 | 1 | - |
| Haematopoietic and lymphoid | NS | Lymphoid neoplasm | T cell large granular lymphocytic leukaemia | 0.00 | 2 | - |
