# Supplementary material for: The dual role of CD70 in B‐cell lymphomagenesis
Source: Clin Transl Med. 2022 Dec 5;12(12):e1118. doi: 10.1002/ctm2.1118 (PMC9722974; doi:10.1002/ctm2.1118)
Supplement: Supplementary file 7 — Supporting Information [file CTM2-12-e1118-s009.docx]

**S6. Baseline characteristics of DLBCL patients with different *CD70* genetic statuses.** S6a. Baseline characteristics of Chinese DLBCL patients with different CD70 genetic statuses. S6b. Univariate and multivariate analysis of OS for Chinese DLBCL patients.

S6c. Baseline characteristics of Swedish DLBCL patients with different CD70 genetic statuses.

| **S6a.Baseline characteristics of Chinese DLBCL patients with different *CD70* genetic statuses.** | | | | | |
| --- | --- | --- | --- | --- | --- |
| Chinese cohort (*n* = 198) | | | | | |
|  | *CD70* genetic altered group | | Wide-type *CD70* group | | *P* |
|  | Number | % | Number | % |  |
| Overall | 48 | 24.24% | 150 | 75.76% |  |
| Gender |  |  |  |  |  |
| Male | 35 | 72.92 | 90 | 60.00 | 0.11 |
| Female | 13 | 27.08 | 60 | 40.00 |  |
| Age |  |  |  |  |  |
| > 60 | 22 | 45.83 | 62 | 41.33 | 0.58 |
| ≤ 60 | 26 | 54.17 | 88 | 58.67 |  |
| **ECOG score*** |  |  |  |  |  |
| 0-1 | 30 | 62.50 | 123 | 82.00 | **0.01** |
| ≥ 2 | 18 | 37.50 | 27 | 18.00 |  |
| Ann Arbor stage |  |  |  |  |  |
| I/II | 21 | 43.75 | 73 | 48.67 | 0.55 |
| III/IV | 27 | 56.25 | 77 | 51.33 |  |
| **Extranodal involvement*** |  |  |  |  |  |
| Yes | 30 | 62.50 | 59 | 39.33 | **0.01** |
| No | 18 | 37.50 | 91 | 60.67 |  |
| IPI |  |  |  |  |  |
| 0-1 | 20 | 41.67 | 66 | 44.00 | 0.78 |
| 2-5 | 28 | 58.33 | 84 | 56.00 |  |
| B symptoms |  |  |  |  |  |
| Yes | 14 | 31.82 | 35 | 25.93 | 0.35 |
| No | 28 | 63.64 | 100 | 74.07 |  |

Elevated LDH

Subtype

Yes 25 33.33 75 50.00 0.80

No 23 66.67 75 50.00

GCB 22 45.83 59 39.33 0.43

non-GCB 26 54.17 91 60.67

| **HBV infection*** |  | | | | |
| --- | --- | --- | --- | --- | --- |
| HBsAg + | 17 | 35.42 | 27 | 18.00 | **0.01** |
| HBsAg - | 31 | 64.58 | 123 | 82.00 |  |
| Therapeutic regimen |  |  |  |  |  |
| R-CHOP | 24 | 64.86 | 79 | 65.83 | 0.81 |
| CHOP | 13 | 35.14 | 39 | 32.50 |  |
| Treatment response |  |  |  |  |  |
| CR + PR | 34 | 82.93 | 95 | 77.24 | 0.44 |
| SD + PD | 7 | 17.07 | 28 | 22.76 |  |

IPI, international prognostic index; LDH, lactate dehydrogenase.

*χ^2^ test was used for comparison. Significant values (*P* < 0.05) are highlighted in bold.

| **S6b. Univariate and multivariate analysis of OS for Chinese DLBCL patients.** | | | | |
| --- | --- | --- | --- | --- |
| Variable | Univariate |  | Multivariate | |
|  | HR(95%) | P value | HR(95%) | P value |
| CD70 genetic aberration (no/yes) | 1.849 (1.139-3.002) | 0.013 | 1.778 (1.087-2.909) | 0.022 |
| Elevated LDH (no/yes) | 2.705 (1.642-4.457) | 0.000 | 1.712 (0.915-3.203) | 0.093 |
| Extranodal involvement (no/yes) | 1.937 (1.222-3.070) | 0.005 | 1.102 (0.654-1.856) | 0.716 |
| Ann Arbor stage (I/II vs III/IV) | 3.135 (1.871-5.251) | 0.000 | 2.217 (1.189-4.135) | 0.012 |
| Age, years (≤60/≥60) | 2.377 (1.485-3.803) | 0.000 | 1.834 (1.107-3.038) | 0.019 |
| IPI (0-1/2-5) | 3.169 (1.845-5.443) | 0.000 | 1.091 (0.491-2.425) | 0.831 |

| **S6c. Baseline characteristics of Swedish DLBCL patients with different *CD70* genetic statuses.** | | | | |
| --- | --- | --- | --- | --- |
| Swedish cohort (*n* = 84) | | | | |
| *CD70* genetic altered group | | Wide-type *CD70* group | | *P* |
| Number | % | Number | % |  |

| Overall | 9 | 11.11 | 75 | 83.33 |  |
| --- | --- | --- | --- | --- | --- |
| Gender |  |  |  |  |  |
| Male | 3 | 33.3% | 38 | 51.4% | 0.4827 |
| Female | 6 | 66.7% | 36 | 48.6% |  |
| Age |  |  |  |  |  |
| > 60 | 8 | 88.9% | 48 | 64.9% | 0.2595 |
| ≤ 60 | 1 | 11.1% | 26 | 35.1% |  |
| Ann Arbor stage |  |  |  |  |  |
| I/II | 5 | 55.6% | 34 | 49.3% | >0,9999 |
| III/IV | 4 | 44.4% | 35 | 50.7% |  |
| B symptoms |  |  |  |  |  |
| Yes | 3 | 33.3% | 24 | 35.8% | >0,9999 |
| No | 6 | 66.7% | 43 | 64.2% |  |
| Subtype |  |  |  |  |  |
| GCB | 1 | 16.7% | 26 | 50.0% | 0.2007 |
| non-GCB | 5 | 83.3% | 26 | 50.0% |  |
| Therapeutic regimen |  |  |  |  |  |
| R-CHOP like | 1 | 16.7% | 12 | 24.0% | >0,9999 |
| CHOP like | 5 | 83.3% | 38 | 76.0% |  |
| Treatment response |  |  |  |  |  |
| CR | 5 | 62.5% | 49 | 71.0% | 0.6895 |
| no CR | 3 | 37.5% | 20 | 29.0% |  |
| Fisher's exact test was used for comparison. | |  |  |  |  |
