# Supplementary material for: The dual role of CD70 in B‐cell lymphomagenesis
Source: Clin Transl Med. 2022 Dec 5;12(12):e1118. doi: 10.1002/ctm2.1118 (PMC9722974; doi:10.1002/ctm2.1118)
Supplement: Supplementary file 8 — Supporting Information [file CTM2-12-e1118-s001.docx]

| **S7. Baseline characteristics of Swedish DLBCL patients with different CD70 protein expression levels** | | | | | |
| --- | --- | --- | --- | --- | --- |
| Swedish cohort (*n* = 315) | | | | | |
|  | CD70 score (0-2) | | CD70 score (3-8) | | *P* |
|  | Number | % | Number | % |  |
| Overall | 109 | 100 | 206 | 100 |  |
| Gender† |  |  |  |  |  |
| Male | 28 | 60.87 | 81 | 59.12 | 0.8347 |
| Female | 18 | 39.13 | 56 | 40.88 |  |
| Age† |  |  |  |  |  |
| > 60 | 28 | 60.87 | 100 | 72.99 | 0.1207 |
| ≤ 60 | 18 | 39.13 | 37 | 27.01 |  |
| Extranodal involvement |  |  |  |  |  |
| Yes | 34 | 31.78 | 82 | 41.00 | 0.1122 |
| No | 73 | 68.22 | 118 | 59.00 |  |
| **IPI** |  |  |  |  |  |
| 0-1 | 56 | 53.85 | 70 | 36.65 | **0.0043** |
| 2-5 | 48 | 46.15 | 121 | 63.35 |  |
| B symptoms† |  |  |  |  |  |
| Yes | 15 | 34.88 | 50 | 38.17 | 0.6993 |
| No | 28 | 65.12 | 81 | 61.83 |  |
| **Elevated LDH** |  |  |  |  |  |
| Yes | 44 | 43.56 | 116 | 59.49 | **0.0092** |
| No | 57 | 56.44 | 79 | 40.51 |  |
| Subtype |  |  |  |  |  |
| GCB | 19 | 42.22 | 68 | 50.37 | 0.3435 |
| non-GCB | 26 | 57.78 | 67 | 49.63 |  |
| **Treatment response** |  |  |  |  |  |
| CR | 85 | 82.52 | 82 | 63.08 | **0.0011** |
| no CR | 18 | 17.48 | 48 | 36.92 |  |
| **EBV** |  |  |  |  |  |
| positive | 2 | 2.20 | 20 | 13.79 | **0.0023** |
| negative | 89 | 97.80 | 125 | 86.21 |  |

| **Treatment** |  |  |  |  |  |
| --- | --- | --- | --- | --- | --- |
| R-CHOP like | 18 | 18.95 | 65 | 34.21 | **0.0075** |
| CHOP like | 77 | 81.05 | 125 | 65.79 |  |
| CR, complete remission | | | | | |
| IPI, international prognostic index; | | | | | |
| LDH, lactate dehydrogenase. | | | | | |
| The comparison between EBV negative and positive subgroups was performed by Fisher's exact test, and other comparisons were performed by χ^2^ test. | | | | | |
| Significant values (*P* < 0.05) are highlighted in bold. | | | | | |
