# Supplementary material for: The dual role of CD70 in B‐cell lymphomagenesis
Source: Clin Transl Med. 2022 Dec 5;12(12):e1118. doi: 10.1002/ctm2.1118 (PMC9722974; doi:10.1002/ctm2.1118)
Supplement: Supplementary file 9 — Supporting Information [file CTM2-12-e1118-s006.docx]

S8. Sample information and numbers of different subclusters of cells for each sample by single-cell RNA sequencing.

| **Sample ID** | **Gender** | **Molecular subtype** | **Age** | **Ann Arbor Stage** | **Cell number** | **Malignant** | **B cell** | **T cell** | **NK cell** | **Myeloid** | **CAF*** | **Endothelial** |
| --- | --- | --- | --- | --- | --- | --- | --- | --- | --- | --- | --- | --- |
| Case-507 | Male | ABC | 73 | IIA | 5582 | 5128 | 39 | 219 | 22 | 174 | 0 | 0 |
| Case-508 | Male | ABC | 64 | IV | 3361 | 168 | 2028 | 1078 | 9 | 77 | 0 | 1 |
| Case-509 | Male | GCB | 24 | I | 6273 | 4461 | 17 | 1268 | 44 | 213 | 240 | 30 |
| Case-510 | Male | ABC | 48 | II | 3948 | 453 | 316 | 2878 | 94 | 201 | 0 | 6 |
| Case-511 | Female | GCB | 35 | IV | 6601 | 3985 | 46 | 2464 | 32 | 47 | 4 | 23 |
| Case-512 | Female | ABC | 59 | II | 6763 | 2173 | 1525 | 2640 | 110 | 301 | 3 | 11 |
| Case-513 | Female | ABC | 76 | IIIA | 4554 | 976 | 497 | 2922 | 23 | 91 | 5 | 40 |
| Case-514 | Male | ABC | 52 | III | 4219 | 2800 | 20 | 1279 | 27 | 48 | 2 | 43 |
| Case-515 | Male | ABC | 40 | II | 4992 | 3303 | 54 | 1577 | 9 | 44 | 0 | 5 |
| Case-516 | Male | GCB | 66 | IVE | 3729 | 1826 | 33 | 1608 | 96 | 34 | 56 | 76 |
| Case-517 | Male | ABC | 59 | IVE | 5383 | 4886 | 13 | 311 | 10 | 25 | 45 | 93 |
| *CAFs, cancer-associated fibroblasts | | | | | | | | | | | | |
